# 14‐3‐3γ Protects Against Postoperative Cognitive Dysfunction by Regulating Tau Thr205 Phosphorylation and Synaptic Integrity

**DOI:** 10.1002/advs.77480

**Published:** 2026-09-27

**Authors:** Shouqiang Zhu, Xue Han, Heng Zhang, Chang Ye, Tianjiao Xia, Xiaoping Gu

**Affiliations:** ^1^ Department of Anesthesiology, Nanjing Drum Tower Hospital, Affiliated Hospital of Medical School Nanjing University Nanjing China; ^2^ Medical School Nanjing University Nanjing Jiangsu China; ^3^ Jiangsu Key Laboratory of Molecular Medicine Nanjing University Nanjing Jiangsu China

**Keywords:** 14‐3‐3γ, postoperative cognitive dysfunction (POCD), protein‐protein interaction, Tau phosphorylation, Thr205, synaptic integrity

## Abstract

**Background**: 14‐3‐3γ is implicated in neurodegeneration, yet its role in postoperative cognitive dysfunction (POCD) remains unclear.

**Methods**: We performed CSF proteomic profiling using the SomaScan 7K platform in the Alzheimer's Disease Neuroimaging Initiative cohort (n = 237) to identify biomarkers associated with neurodegeneration‐enriched cognitive vulnerability (NECV). A prospective surgical cohort (n = 213) was used to evaluate circulating 14‐3‐3γ levels in relation to perioperative cognitive outcomes. Mechanistic studies were conducted using a murine surgery model, AAV‐mediated gene manipulation, neuronal cultures, pharmacological intervention, and molecular dynamics simulations.

**Results**: CSF proteomics identified 14‐3‐3γ as a candidate biomarker associated with NECV and neurodegenerative progression. Plasma 14‐3‐3γ levels were associated with POCD and postoperative delirium. In mice, anesthesia and surgery reduced hippocampal 14‐3‐3γ expression, accompanied by increased Tau phosphorylation at Thr205 among examined sites, synaptic dysfunction, and cognitive impairment. Neuronal 14‐3‐3γ overexpression rescued these deficits. Mechanistically, 14‐3‐3γ interacted with Tau in a phosphorylation‐dependent manner and regulated Tau phosphorylation homeostasis. Pharmacological stabilization of the 14‐3‐3γ–Tau interaction using FC‐A reduced Tau Thr205 phosphorylation and improved cognition, whereas TauT205E expression diminished this rescue effect.

**Conclusions**: 14‐3‐3γ represents a potential regulator of postoperative cognitive vulnerability and a candidate therapeutic target for POCD.

## Graphical abstract

Integrative multi‐cohort analyses identify 14‐3‐3γ as a biomarker and regulator of cognitive vulnerability. Experimental studies show that 14‐3‐3γ loss is associated with Tau Thr205 phosphorylation, synaptic dysfunction, and cognitive impairment, while genetic overexpression or pharmacological stabilization of 14‐3‐3γ confers protective effects in experimental models. BioRender elements were used under license (https://www.biorender.com/).

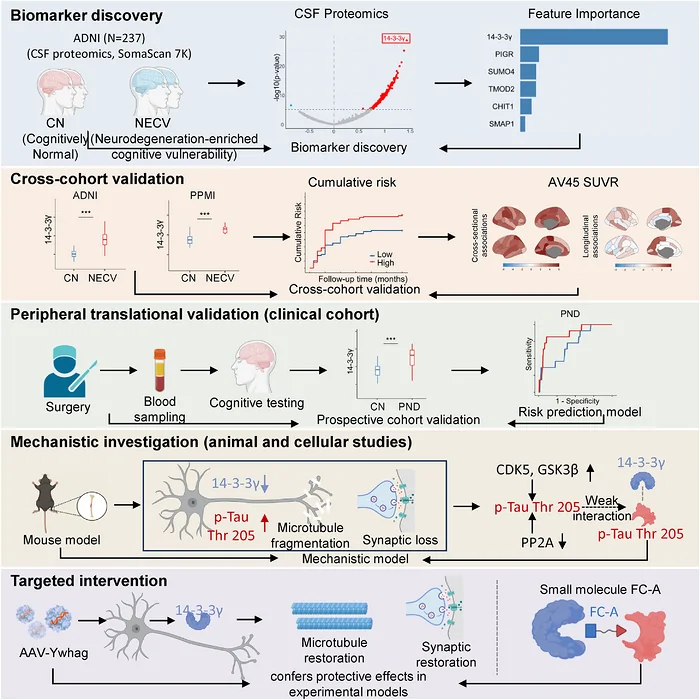

## 1 Introduction

Postoperative cognitive dysfunction (POCD) is a common and clinically significant complication following surgery, particularly among older adults, and is associated with prolonged hospitalization, loss of independence, reduced quality of life, and increased mortality [1, 2, 3, 4, 5]. Despite the large number of elderly individuals undergoing surgery each year, only a subset develops persistent postoperative cognitive decline, indicating marked inter‐individual differences in susceptibility to perioperative brain injury [1]. However, the biological factors that confer vulnerability or resilience to postoperative cognitive decline remain poorly understood. Identifying molecular determinants of postoperative cognitive vulnerability is therefore a major unmet need and may enable risk stratification, early diagnosis, and targeted intervention. These observations suggest the existence of a latent biological susceptibility to postoperative cognitive decline, which we refer to as neurodegeneration‐enriched cognitive vulnerability (NECV). Understanding the molecular basis of NECV may provide critical insights into why some individuals develop postoperative cognitive impairment whereas others remain cognitively resilient despite comparable surgical exposure.

Despite its substantial clinical burden, POCD remains under‐recognized in routine clinical practice. A systematic review by Sim and colleagues identified only 15 studies investigating preoperative biomarkers for POCD in older adults, with marked heterogeneity in cognitive outcome metrics and assessment timeframes limiting quantitative synthesis [6]. The same review noted that while amyloid biomarkers show promise, evidence for other biomarker classes remains limited, and no single biomarker has been validated for routine clinical use [7]. Consequently, current diagnosis relies primarily on neuropsychological testing, which is time‐consuming, subject to practice effects, and difficult to standardize across clinical settings. Importantly, such assessments often fail to capture early or subclinical cognitive decline, limiting their utility for perioperative decision‐making. Although a range of candidate biomarkers—including inflammatory cytokines, S100β, neuron‐specific enolase, and neurofilament light chain—have been investigated, their predictive value remains inconsistent and insufficient for clinical translation; considerable heterogeneity across studies in sampling time points, assay methods, and cognitive outcomes reduces the reproducibility and comparability of biomarker findings [8, 9, 10]. Therefore, identifying consistent, reproducible, and clinically accessible biomarkers that reflect perioperative brain vulnerability represents a critical unmet need.

Accumulating evidence suggests that POCD shares overlapping molecular mechanisms with neurodegenerative disorders, particularly those involving synaptic dysfunction, neuroinflammation, and Tau pathology [11, 12, 13]. Surgical stress and anesthesia have been shown to induce Tau hyperphosphorylation, microtubule destabilization, and synaptic loss, processes that closely resemble early pathological changes observed in Alzheimer's disease (AD) [14, 15, 16]. Notably, perioperative increases in phosphorylated Tau have been detected in both cerebrospinal fluid (CSF) and experimental models, and are associated with subsequent cognitive decline [17, 18]. However, the upstream molecular regulators that link surgical insults to Tau dysregulation and neuronal vulnerability remain poorly understood. Several studies have investigated blood‐based tau and phosphorylated tau as potential biomarkers for PND, a systematic review of preoperative biofluid biomarkers identified p‐Tau181 as a candidate, although small sample sizes and conflicting results limited definitive conclusions [6].

In this context, unbiased proteomic approaches offer a powerful strategy to identify novel biomarkers and pathways underlying cognitive impairment. CSF proteomics, in particular, provides a direct window into central nervous system biochemical changes. Recent large‐scale analyses have demonstrated that CSF protein signatures can outperform traditional biomarkers in predicting cognitive decline and disease progression [19, 20]. Notably, a multiplex proteomic study based on the Alzheimer's Disease Neuroimaging Initiative (ADNI) cohort identified YWHAG, encoding 14‐3‐3γ, as one of the most consistent biomarkers for both diagnosis and prediction of cognitive decline, with strong associations with Tau pathology, amyloid burden, and cognitive performance [21]. These findings suggest that 14‐3‐3γ may represent a central node linking neuronal stress to cognitive dysfunction. However, its role in POCD has not been investigated.

The 14‐3‐3 protein family comprises highly conserved phospho‐serine/threonine–binding proteins that regulate a wide range of cellular processes through protein–protein interactions. In the brain, 14‐3‐3 proteins are highly abundant and play essential roles in synaptic plasticity, cytoskeletal organization, and neuronal survival [22, 23]. Importantly, growing evidence indicates that 14‐3‐3 proteins directly interact with Tau and modulate its phosphorylation, aggregation, and microtubule binding dynamics [24]. For instance, 14‐3‐3 binding has been shown to regulate phospho‐Tau solubility and prevent pathological aggregation, thereby exerting neuroprotective effects [25]. Furthermore, pharmacological stabilization of 14‐3‐3 protein–protein interactions using small molecules such as fusicoccin‐A (FC‐A) has been demonstrated to reduce neuronal toxicity and improve neuronal survival in neurodegenerative models [26]. In parallel, experimental studies have shown that 14‐3‐3γ exerts anti‐apoptotic and neuroprotective effects under stress conditions, including ischemic injury, by modulating key signaling pathways [27]. Despite these advances, whether 14‐3‐3γ regulates Tau pathology and synaptic integrity in the context of surgical stress remains unknown.

Another critical barrier to clinical translation is that most biomarker discoveries in neurodegeneration are confined to CSF, which is invasive and impractical for routine perioperative use. Therefore, identifying biomarkers that can be reliably detected in peripheral blood and retain predictive value is essential for real‐world application. However, the relationship between central and peripheral expression of candidate proteins, particularly under acute surgical stress, remains largely unexplored.

To address these challenges, we conducted a comprehensive, multi‐level investigation integrating human proteomics, clinical validation, and mechanistic studies. We first performed large‐scale CSF proteomic analyses across multiple cohorts to identify candidate biomarkers associated with NECV. We then translated these findings into a prospective surgical cohort to investigate whether early postoperative plasma 14‐3‐3γ levels were associated with subsequent POCD. Finally, we employed animal and cellular models to elucidate the mechanistic role of 14‐3‐3γ in regulating Tau phosphorylation, synaptic integrity, and cognitive function, and explored pharmacological targeting of the 14‐3‐3γ–Tau interaction. We hypothesized that (1) CSF 14‐3‐3γ levels would be elevated in individuals with NECV; (2) early postoperative plasma 14‐3‐3γ would be associated with subsequent POCD and POD in a prospective surgical cohort; (3) hippocampal 14‐3‐3γ depletion following surgery would be associated with increased Tau Thr205 phosphorylation and alterations in CDK5/GSK3β/PP2A signaling; and (4) in a murine model, pharmacological stabilization of the 14‐3‐3γ‐tau interaction would ameliorate surgery‐induced cognitive deficits.

## 2 Methods

### 2.1 Biomarker Discovery in the ADNI Cohort

#### 2.1.1 Data Source and Study Population

Data for this study were obtained from the Alzheimer's Disease Neuroimaging Initiative (ADNI; http://adni.loni.usc.edu/) and the Parkinson's Progression Markers Initiative (PPMI; http://www.ppmi‐info.org/study‐design/). The authors (SQ.Z.) obtained authorized access to the ADNI and PPMI dataset and conducted all analyses in accordance with the ADNI and PPMI data‐use agreement and publication policies. ADNI and PPMI are multicentre longitudinal observational cohorts designed to facilitate biomarker discovery in neurodegenerative diseases. ADNI enrolls participants aged 55–90 across 57 sites in the United States and Canada and collects standardized clinical, neuropsychological, imaging, genetic, and biochemical data to track progression from normal cognition to mild cognitive impairment (MCI) and Alzheimer's disease (AD) dementia. PPMI recruits cognitively normal (CN) participants with similar standardized assessments to allow independent validation of AD‐related findings. All procedures were approved by the local institutional review boards of participating sites, and written informed consent was obtained from all participants or their authorized representatives.

For the ADNI‐based discovery analysis, no formal a priori sample size calculation was performed. Instead, we included all participants from the ADNI database who met the following criteria: (1) complete CSF proteomic profiling using the SomaScan 7K platform, (2) available CSF Aβ42 and p‐tau181 measurements, and (3) documented surgical history to allow classification into Neurodegeneration‐enriched cognitive vulnerability (NECV) or cognitively normal controls (CN) groups. This selection process yielded a final sample of 237 participants (152 NECV, 85 CN). CN participants were defined as MMSE ≥24 and Clinical Dementia Rating (CDR) = 0, MCI participants were defined according to established criteria, including objective memory impairment assessed by the Wechsler Memory Scale Logical Memory II delayed recall, a CDR score of 0.5, preserved activities of daily living, and absence of dementia, while AD dementia was diagnosed based on NINCDS/ADRDA criteria with MMSE 20–26 and CDR 0.5–1.0. To enhance signal‐to‐noise ratio during high‐dimensional proteomic screening, the discovery analysis was restricted to participants representing biologically distinct AT extremes (NECV A+T+ versus CN A−T−), Amyloid (A) and tau (T) status were determined according to established ADNI CSF biomarker cutoffs based on CSF Aβ42 and p‐tau181 concentrations. This strategy minimizes molecular heterogeneity introduced by intermediate AT categories and increases the likelihood of identifying proteins most strongly associated with neurodegeneration‐enriched cognitive vulnerability in the context of Alzheimer's disease pathology.

#### 2.1.2 Definition of Neurodegeneration‐Enriched Cognitive Vulnerability (NECV)

Because the ADNI cohort was not designed to investigate perioperative neurocognitive disorders and does not include detailed perioperative information, including surgical timing, anesthesia exposure, preoperative cognitive status, or postoperative cognitive trajectories, formal diagnosis of postoperative cognitive dysfunction (POCD) was not possible. Therefore, the ADNI‐based analysis was not intended to identify POCD cases. Instead, we performed a hypothesis‐generating, neurodegeneration‐enriched discovery analysis by identifying individuals with cognitive impairment consistent with mild cognitive impairment (MCI) who also had a documented history of surgery exposure. This subgroup was defined as neurodegeneration‐enriched cognitive vulnerability (NECV) and was used to enrich for individuals with cognitive vulnerability in the context of prior surgical exposure, rather than to establish a causal relationship between surgery and cognitive decline. Accordingly, NECV should be interpreted as an exploratory phenotype representing the coexistence of cognitive impairment and prior surgery history within a neurodegeneration‐enriched population, rather than a clinical diagnosis of POCD or a direct measure of surgery‐associated cognitive vulnerability [28].

#### 2.1.3 CSF Proteomic Profiling and Statistical Analysis

CSF proteomic profiling was performed using the SomaScan 7K SOMAmer‐based aptamer platform (SomaLogic), measuring 7,584 analytes corresponding to 6,361 unique proteins. CSF samples were stored at −80°C and processed following standard protocols. Raw relative fluorescence unit (RFU) values underwent hybridization normalization, median scaling, and iterative adaptive normalization to remove technical variance while preserving biological differences. Quality control excluded poorly measured analytes and low‐quality samples, resulting in 7,008 analytes across 237 baseline participants. PPMI CSF proteomics were processed using a proprietary SomaScan platform, with hybridization normalization, plate scaling, median normalization, log2 transformation, and batch correction. CSF Aβ42, p‐tau181, and t‐tau levels were quantified using Roche Elecsys automated immunoassays in both cohorts. All assays were performed in duplicate, with intra‐ and inter‐lot coefficients of variation <15%.

Differential protein analysis to identify NECV‐associated proteins was conducted using multiple linear regression models on log‐transformed and Z‐scored protein levels, adjusting for age, sex, education, and APOE ε4 status. Bonferroni correction was applied for multiple comparisons (n = 6,361 proteins). Sensitivity analyses further adjusted for CSF hemoglobin levels to control for potential blood contamination. Highly correlated proteins (Spearman r >0.7) were clustered, with the protein having the highest discriminative area under the curve (AUC) retained per cluster.

The top‐ranked protein, CSF 14‐3‐3γ, was validated across PPMI and postmortem‐confirmed AD samples. Its association with disease progression was assessed using Cox proportional hazards models and Kaplan–Meier analyses for CN → prodromal AD (CDR ≥0.5) and NECV → AD dementia transitions, with participants dichotomized by median protein levels and models adjusted for age, sex, education, and APOE ε4 status. Cross‐sectional and longitudinal associations of 14‐3‐3γ with CSF AD biomarkers, amyloid PET (AV45), tau PET (AV1451), FDG‐PET, MRI‐derived hippocampal volume and cortical thickness, and cognitive measures (ADNI composite memory, executive function, and PACC scores) were examined using multiple linear regression and linear mixed‐effects models. Longitudinal models included time × protein interaction terms, and all outcomes (except cognitive scores) were log‐transformed and Z‐scored. Analyses were adjusted for age, sex, education, APOE ε4, and intracranial volume for volumetric measures.

#### 2.1.4 Neuroimaging Analysis

Amyloid PET imaging data were obtained from the ADNI database and processed using the standardized ADNI PET pipeline. Briefly, individual structural MRI scans were segmented and parcellated using FreeSurfer (version 7.1.1) to define anatomical regions of interest (ROIs) in native space. Florbetapir ([18F]AV45) PET images were co‐registered with corresponding MRI scans, and regional standardized uptake value ratios (SUVRs) were extracted from predefined cortical ROIs based on the Desikan–Killiany atlas. For amyloid PET quantification, SUVR values were normalized using a composite reference region consisting of whole cerebellum, brainstem/pons, and eroded subcortical white matter, as previously established in ADNI studies. Associations between CSF 14‐3‐3γ concentrations and regional AV45 SUVR values were examined using multivariable linear regression models, with regional SUVR as the dependent variable and CSF 14‐3‐3γ as the independent variable. Models were adjusted for age, sex, years of education, and APOE ε4 carrier status. ROI‐specific analyses included all participants with available CSF 14‐3‐3γ measurements and quality‐controlled AV45 PET data. Multiple comparisons across cortical ROIs were controlled using the Benjamini–Hochberg false discovery rate (FDR) procedure.

### 2.2 Peripheral Translational Validation

#### 2.2.1 Sample Size Calculation

As this was an exploratory observational study designed to evaluate the association between circulating 14‐3‐3γ levels and postoperative neurocognitive outcomes, a formal a priori sample size calculation was not performed. we consecutively enrolled all eligible patients who met the inclusion criteria during a predefined recruitment period of 6 months at a single tertiary academic center. Participants meeting the predefined eligibility criteria and providing informed consent were enrolled consecutively during the study period. A total of 213 patients were enrolled during this period, which is comparable to or larger than previous prospective biomarker studies in postoperative cognitive dysfunction [10, 18, 29]. This consecutive sampling approach was chosen to minimize selection bias and to reflect the real‐world population of patients undergoing major elective surgery at our institution.

#### 2.2.2 Inclusion and Exclusion Criteria

The study included a prospective cohort of 213 patients undergoing major elective surgery at the Affiliated Drum Tower Hospital of Nanjing University Medical School. The study protocol was approved by the hospital ethics committee (approval number: 2024‐902‐02) and registered at the Chinese Clinical Trial Registry (ChiCTR2500096293). Written informed consent was obtained from all participants or their legal representatives. Inclusion criteria: (1) age ≥ 65 years; (2) scheduled for elective major surgery (cardiac, orthopedic, or aortic surgery) under general anesthesia with expected duration ≥ 2 hours; (3) American Society of Anesthesiologists (ASA) physical status I‐III; (4) ability to provide written informed consent; (5) ability to complete preoperative neuropsychological assessments. Exclusion criteria: (1) documented diagnosis of dementia or other major neurodegenerative disorders; (2) severe psychiatric illness (e.g., schizophrenia or bipolar disorder); (3) inability to complete neuropsychological assessments because of severe communication barriers, profound visual or hearing impairment, or severe neurological deficits; (4) history of severe traumatic brain injury or intracranial surgery; (5) alcohol or substance abuse disorder; (6) emergency surgery; and (7) refusal to participate.

#### 2.2.3 Outcome Definitions: POD and POCD

Detailed baseline demographic, clinical, and intraoperative characteristics are presented in Tables 1 and 2. Patients underwent comprehensive preoperative evaluation including medical history, laboratory measurements (e.g., hemoglobin, platelet count, electrolytes, renal function), comorbidities, functional status, and planned surgical and anesthesia parameters. Functional and psychological assessments included the Instrumental Activities of Daily Living (IADL) scale, the Perceived Stress Index (PSI), the Patient Health Questionnaire‐4 (PHQ‐4) and frailty score. Intraoperative variables including anesthesia duration, surgical duration, fluid and blood management, and drug dosages were recorded.

**TABLE 1:** Baseline Characteristics of Patients in the Prospective Surgical Cohort According to Postoperative Cognitive Dysfunction (POCD).

Characteristic	Non‐POCD(n = 202)	POCD (n = 11)	*p* Value
Demographic Characteristics			
Age, median (IQR), y	71.0 (68.0–76.0)	77.0 (74.5–79.5)	0.004
Male sex, No. (%)	122 (60.4)	5 (45.5)	0.357
Body mass index, median (IQR), kg/m^2^	23.5 (21.5–25.6)	24.6 (21.9–25.6)	0.608
Socioeconomic Status			
Education level, No. (%)			0.091
— Primary school or below	98 (48.5)	9 (81.8)	
— Middle school	42 (20.8)	0 (0.0)	
— High school or above	62 (30.7)	2 (18.2)	
Living alone, No. (%)	13 (6.4)	1 (9.1)	0.535
Preoperative Clinical Status			
ASA physical status (I–II), No. (%)	177 (87.6)	11 (100)	0.369
Smoking history, No. (%)	46 (22.8)	2 (18.2)	1
Alcohol consumption, No. (%)	53 (26.2)	3 (27.3)	1
Comorbidities			
Hypertension, No. (%)	116 (57.4)	10 (90.9)	0.03
Diabetes mellitus, No. (%)	34 (16.8)	2 (18.2)	1
Coronary artery disease, No. (%)	78 (38.6)	8 (72.7)	0.031
Acute myocardial infarction, No. (%)	24 (11.9)	3 (27.3)	0.15
Cerebral infarction, No. (%)	44 (21.8)	3 (27.3)	0.71
Atrial fibrillation, No. (%)	44 (21.8)	4 (36.4)	0.273
Chronic kidney disease, No. (%)	12 (5.9)	2 (18.2)	0.157
Heart failure (EF <40%), No. (%)	10 (5.0)	1 (9.1)	0.45
History of fall (past 6 months), No. (%)	21 (10.4)	1 (9.1)	1
Cancer, No. (%)	9 (4.5)	0 (0.0)	1
Laboratory Measurements			
Hemoglobin, median (IQR), g/L	127 (113–140)	126 (104–137)	0.271
Platelet count, median (IQR), ×10^9^/L	176 (149–210)	158 (118–216)	0.326
Triglycerides, median (IQR), mmol/L	1.10 (0.82–1.39)	1.37 (1.01–1.67)	0.181
Low‐density lipoprotein cholesterol, mmol/L	2.27 (1.78–2.70)	1.99 (1.73–2.77)	0.906
C‐reactive protein, median (IQR), mg/L	6.20 (3.62–14.0)	7.20 (3.75–24.3)	0.639
Serum potassium, median (IQR), mmol/L	3.93 (3.67–4.11)	4.11 (3.91–4.50)	0.087
Serum creatinine, median (IQR), µmol/L	76.0 (64.0–88.8)	86.0 (68.0–105)	0.205
eGFR, median (IQR), mL/min/1.73 m^2^	83.1 (72.7–97.0)	74.7 (50.2–86.4)	0.042
Functional and Psychological Assessments			
PSI score, median (IQR)	7.00 (5.00–8.00)	9.00 (7.00–10.0)	0.019
PHQ‐4 score, median (IQR)	0.00 (0.00–2.00)	1.00 (0.00–2.50)	0.288
IADL score, median (IQR)	8.00 (8.00–8.00)	8.00 (7.50–8.00)	0.015
Frailty score, median (IQR)	1.00 (1.00–2.00)	2.00 (1.00–2.50)	0.055
Intraoperative Variables			
Surgery type, No. (%)			0.735
— Orthopedic surgery	20 (9.9)	0 (0.0)	
— Coronary artery bypass grafting (CABG)	58 (28.7)	4 (36.4)	
— Valve surgery	91 (45.0)	5 (45.5)	
— Aortic surgery	23 (11.4)	1 (9.1)	
— Other surgery	10 (5.0)	1 (9.1)	
Anesthesia time, median (IQR), min	322 (255–399)	380 (340–450)	0.038
Surgery duration, median (IQR), min	278 (210–340)	320 (290–372)	0.04
Midazolam dose, median (IQR), mg	4.00 (2.00–5.00)	5.00 (2.50–7.00)	0.397
Sufentanil dose, median (IQR), µg	200 (110–300)	250 (105–285)	0.86
Dexmedetomidine dose, median (IQR), µg	0.00 (0.00–0.00)	0.00 (0.00–33.0)	0.622
Intraoperative Fluid and Blood Management			
Crystalloid infusion, median (IQR), mL	100 (0–500)	0 (0–0)	0.002
Colloid infusion, median (IQR), mL	1500 (1025–2000)	1500 (1000–1750)	0.377
Autologous transfusion, median (IQR), mL	50.0 (0–50.0)	50.0 (50.0–458)	0.168
Allogeneic transfusion, median (IQR), mL	0 (0–1019)	600 (0–1400)	0.321
Estimated blood loss, median (IQR), mL	800 (500–1000)	900 (700–1000)	0.336
Urine output, median (IQR), mL	800 (500–1300)	500 (375–750)	0.065

Data are presented as median (interquartile range) or number (percentage). Continuous variables were compared using Wilcoxon rank‐sum tests. Categorical variables were compared using Pearson χ^2^ tests or Fisher's exact tests, as appropriate.

POCD indicates postoperative cognitive dysfunction; ASA, American Society of Anesthesiologists; eGFR, estimated glomerular filtration rate; PHQ‐4, Patient Health Questionnaire‐4; IADL, Instrumental Activities of Daily Living.

Surgery type was categorized as follows: orthopedic surgery; coronary artery bypass grafting (CABG); valve surgery; aortic surgery; and other surgery.

**TABLE 2:** Baseline Characteristics of Patients in the Prospective Surgical Cohort According to Postoperative Delirium (POD).

Characteristic	Non‐POD (n = 191)	POD (n = 22)	*p* Value
Demographic Characteristics			
Age, median (IQR), y	72.0 (68.0–76.0)	72.5 (69.2–75.8)	0.496
Male sex, No. (%)	122 (63.9)	5 (22.7)	<0.001
Body mass index, median (IQR), kg/m^2^	23.6 (21.5–25.7)	23.4 (20.5–25.3)	0.529
Socioeconomic Status			
Education level, No. (%)			0.022
— Primary school or below	90 (47.1)	17 (77.3)	
— Middle school	39 (20.4)	3 (13.6)	
— High school or above	62 (32.5)	2 (9.09)	
Living alone, No. (%)	13 (6.81)	1 (4.55)	1
Preoperative Clinical Status			
ASA physical status (I–II), No. (%)	167 (87.4)	21 (95.5)	0.483
Smoking history, No. (%)	43 (22.5)	5 (22.7)	1
Alcohol consumption, No. (%)	51 (26.7)	5 (22.7)	0.884
Comorbidities			
Hypertension, No. (%)	112 (58.6)	14 (63.6)	0.824
Diabetes mellitus, No. (%)	30 (15.7)	6 (27.3)	0.224
Coronary artery disease, No. (%)	77 (40.3)	9 (40.9)	1
Acute myocardial infarction, No. (%)	24 (12.6)	3 (13.6)	0.747
Cerebral infarction, No. (%)	40 (20.9)	7 (31.8)	0.278
Atrial fibrillation, No. (%)	38 (19.9)	10 (45.5)	0.013
Chronic kidney disease, No. (%)	8 (4.19)	6 (27.3)	0.001
Heart failure (EF <40%), No. (%)	6 (3.14)	5 (22.7)	0.002
History of fall (past 6 months), No. (%)	20 (10.5)	2 (9.09)	1
Cancer, No. (%)	9 (4.71)	0 (0.00)	0.602
Laboratory Measurements			
Hemoglobin, median (IQR), g/L	128 (114–140)	114 (102–138)	0.031
Platelet count, median (IQR), ×10^9^/L	176 (152–213)	152 (110–216)	0.084
Triglycerides, median (IQR), mmol/L	1.10 (0.83–1.40)	1.08 (0.82–1.44)	0.673
Low‐density lipoprotein cholesterol, mmol/L	2.27 (1.80–2.73)	2.12 (1.67–2.40)	0.146
C‐reactive protein, median (IQR), mg/L	6.20 (3.60–12.9)	10.4 (4.12–30.9)	0.155
Serum potassium, median (IQR), mmol/L	3.90 (3.67–4.10)	4.14 (3.77–4.44)	0.014
Serum creatinine, median (IQR), µmol/L	75.0 (63.5–86.0)	94.0 (83.0–139)	<0.001
eGFR, median (IQR), mL/min/1.73 m^2^	83.4 (74.5–97.7)	58.3 (42.4–81.5)	<0.001
Functional and Psychological Assessments			
PSI score, median (IQR)	7.00 (5.00–8.00)	7.00 (5.00–8.75)	0.752
PHQ‐4 score, median (IQR)	0.00 (0.00–1.50)	0.50 (0.00–2.00)	0.169
IADL score, median (IQR)	8.00 (8.00–8.00)	8.00 (8.00–8.00)	0.843
Frailty score, median (IQR)	1.00 (1.00–2.00)	2.00 (1.00–2.00)	0.001
Intraoperative Variables			
Surgery type, No. (%)			0.582
— Orthopedic surgery	20 (10.5)	0 (0.0)	
— Coronary artery bypass grafting (CABG)	55 (28.8)	7 (31.8)	
— Valve surgery	84 (44.0)	12 (54.5)	
— Aortic surgery	22 (11.5)	2 (9.09)	
— Other surgery	10 (5.24)	1 (4.55)	
Anesthesia time, median (IQR), min	315 (250–380)	450 (398–521)	<0.001
Surgery duration, median (IQR), min	265 (202–320)	380 (348–446)	<0.001
Midazolam dose, median (IQR), mg	4.00 (2.00–5.50)	5.00 (3.00–5.75)	0.188
Sufentanil dose, median (IQR), µg	200 (105–300)	200 (128–290)	0.576
Dexmedetomidine dose, median (IQR), µg	0.00 (0.00–11.5)	0.00 (0.00–0.00)	0.032
Intraoperative Fluid and Blood Management			
Crystalloid infusion, median (IQR), mL	100 (0–500)	300 (0–700)	0.113
Colloid infusion, median (IQR), mL	1500 (1000–2000)	2000 (1500–2000)	0.099
Autologous transfusion, median (IQR), mL	50.0 (0–50.0)	50.0 (50.0–162)	0.13
Allogeneic transfusion, median (IQR), mL	0 (0–925)	1062 (525–1862)	<0.001
Estimated blood loss, median (IQR), mL	800 (500–1000)	1100 (900–1575)	<0.001
Urine output, median (IQR), mL	700 (400–1300)	975 (600–1275)	0.217

Data are presented as median (interquartile range) or number (percentage). Continuous variables were compared using Wilcoxon rank‐sum tests. Categorical variables were compared using Pearson χ^2^ tests or Fisher's exact tests, as appropriate.

POD indicates postoperative delirium; ASA, American Society of Anesthesiologists; eGFR, estimated glomerular filtration rate; PHQ‐4, Patient Health Questionnaire‐4; IADL, Instrumental Activities of Daily Living.

Surgery type was categorized as follows: orthopedic surgery; coronary artery bypass grafting (CABG); valve surgery; aortic surgery; and other surgery.

The main outcomes were postoperative delirium (POD) and postoperative cognitive dysfunction (POCD). POD was assessed using the Confusion Assessment Method (CAM) or CAM for the Intensive Care Unit (CAM‐ICU) as appropriate. For patients admitted to the intensive care unit (ICU) postoperatively, CAM‐ICU was administered twice daily by trained ICU nursing personnel. For patients on regular wards, the standard CAM was used. All assessments were performed for up to 7 days postoperatively or until hospital discharge. POCD was determined based on changes in cognitive function from preoperative baseline, assessed using the Mini‐Mental State Examination (MMSE) and Montreal Cognitive Assessment (MoCA) [30, 31, 32]. Postoperative cognitive assessments were performed on postoperative day 7 or prior to discharge. Cognitive changes were quantified using a Z‐score approach based on the change from preoperative baseline performance in each test. For each participant, Z‐scores were calculated as the difference between postoperative and preoperative scores divided by the standard deviation of baseline scores. A composite Z‐score was then derived by averaging across cognitive tests. POCD was defined as a composite Z‐score ≤ ‐1.96 or a Z‐score ≤ ‐1.96 in at least two individual tests, indicating significant cognitive decline [30, 31, 32]. This definition follows established criteria for postoperative cognitive dysfunction [30] and aligns with the 2018 Nomenclature Consensus Working Group recommendations, which specify that cognitive decline within 30 days post‐surgery is termed delayed neurocognitive recovery (dNCR) [30]. However, we retained the term POCD throughout this study for two reasons: (1) to ensure consistency with prior clinical literature, facilitating cross‐study comparison; and (2) to maintain terminological alignment between our clinical cohort and animal experiments, as our murine POCD model captures an acute postoperative cognitive deficit conceptually analogous to human POCD.

#### 2.2.4 Plasma 14‐3‐3γ Measurement

Blood samples were collected on postoperative day 1 before the scheduled the first daily delirium assessment (CAM or CAM‐ICU), using EDTA‐anticoagulated tubes. Therefore, plasma 14‐3‐3γ measurement preceded the first documented episode of POD in all patients who subsequently developed delirium. Plasma was separated within 30 min after collection by centrifugation at 1,000 × g for 15 min at 2–8°C. The isolated plasma samples were aliquoted and stored at −80°C until analysis. Samples were stored for less than 4 months before measurement and underwent no more than two freeze–thaw cycle. Plasma 14‐3‐3γ/YWHAG concentrations were quantified using a commercially available human 14‐3‐3 protein gamma (YWHAG) enzyme‐linked immunosorbent assay (ELISA) kit (HB4177‐Hu, Hnybio, China) according to the manufacturer's instructions. This assay is based on a double‐antibody sandwich ELISA principle, in which plasma YWHAG is captured by immobilized anti‐human YWHAG antibodies and detected using horseradish peroxidase (HRP)‐conjugated detection antibodies. Absorbance was measured at 450 nm using a microplate reader, and concentrations were calculated from a standard curve generated in each assay run. All samples were analyzed in duplicate, and laboratory personnel performing ELISA measurements were blinded to clinical outcomes (POD and POCD status). According to the manufacturer's validation data, intra‐assay and inter‐assay coefficients of variation were <3.5% and <8.9%, respectively. The reported recovery rate in EDTA plasma samples ranged from 95% to 105%. A detailed overview of all cohorts, sample sources, assay platforms, and their contributions to each analysis is provided in Table S1 (Supporting File 2).

### 2.3 Animals and Experimental Design

#### 2.3.1 Animals and Surgical Procedures

Three‐month‐old male C57BL/6 mice (20–25 g) were obtained from the Nanjing Ziyuan Laboratory Animal Technology Co., Ltd. Mice were housed under standard conditions (12 h light/dark cycle, ad libitum access to food and water) and acclimated for 7 days prior to experimentation. All experimental procedures were approved by the Institutional Animal Care and Use Committee (IACUC) of Nanjing Drum Tower Hospital and were conducted in accordance with ARRIVE guidelines. Young adult mice were chosen for the initial mechanistic studies to minimize the confounding effects of age‐related neurodegeneration and to establish causality in a system with intact baseline cognitive function. The use of male mice only was to avoid potential confounding from estrous cycle‐related hormonal fluctuations on tau phosphorylation and synaptic plasticity [33, 34].

Mice were randomly assigned to control (sham) or surgery groups. Tibial fracture with intramedullary fixation was performed under general anesthesia (isoflurane, 1.5%–2.0%) as previously described [35, 36, 37]. This model was selected because it recapitulates several clinically relevant features of major orthopedic surgery in patients, including anesthesia exposure, peripheral tissue injury, inflammation, and postoperative pain, which are considered important contributors to perioperative neurocognitive disorders. Although alternative surgical models, such as exploratory laparotomy or vascular surgical models, have also been widely used to investigate surgery‐induced cognitive impairment, each model represents distinct clinical scenarios and has specific advantages and limitations. In the present study, we selected the tibial fracture model because of its established relevance to perioperative neurocognitive disorders and its reproducibility in our previous investigations [35, 36, 37]. To minimize acute surgical pain, 2% lidocaine solution was locally applied to the incision site before surgery. No systemic postoperative analgesics were administered during the experimental period. Mice were carefully monitored after surgery, and behavioral testing was conducted after recovery from anesthesia. Behavioral tests were conducted on postoperative days 1, 3, and 7. The Animal Ethics Committee of Nanjing Drum Tower Hospital approved the animal feeding and experimental operation in this study. The approval time for animal ethics was April 7th, 2025, and the number was 2025AE01169. Detailed information on all antibodies, pharmacological agents, and critical reagents, including vendors and catalog numbers, is provided in Table S5 (Supporting File 2) to ensure reproducibility.

#### 2.3.2 Behavioral Assessments

Open Field Test (OFT): General locomotor activity was measured using an open field arena (40 × 40 × 40 cm). Mice were allowed to freely explore for 5 min, and total distance traveled and movement patterns were recorded using an automated video tracking system (EthoVision XT, Noldus Information Technology).

Y‐Maze Spontaneous Alternation: Hippocampus‐dependent spatial working memory was assessed using a Y‐maze (arms: 35 cm × 5 cm × 15 cm). Mice were placed at the end of one arm and allowed to explore freely for 8 min. Spontaneous alternation percentage was calculated as the number of successive triad entries into all three arms divided by the total possible alternations × 100. Behavioral tracking and heatmap generation were performed using EthoVision XT (Noldus Information Technology).

Fear Conditioning (FC): Contextual and cued memory were assessed using a fear conditioning system (XinRuan Information Technology, China). Mice were exposed to a conditioned stimulus (tone) paired with a foot shock (0.7 mA, 2 s) for 3 cycles. Contextual memory was assessed 24 h later by returning mice to the same chamber without shock and measuring freezing behavior. Cued memory was assessed in an altered context with tone presentation. Freezing behavior was automatically quantified using the same system.

#### 2.3.3 Hippocampal Histology and Immunohistochemistry

Golgi Staining: Neuronal morphology and dendritic spines were visualized using a Golgi‐Cox staining kit (FD NeuroTechnologies) following manufacturer instructions. Apical and basal dendrites of hippocampal CA1 neurons were imaged at 100× oil objective, and spine density was quantified manually, classifying mature and immature spines.

Immunofluorescence: Hippocampal sections (20 µm) were fixed with 4% paraformaldehyde, permeabilized, and blocked with 5% BSA. Primary antibodies included anti‐14‐3‐3γ (1:200), anti‐PSD95 (1:500), anti‐synaptophysin (SYP, 1:500), anti‐NeuN (1:500), and anti‐p‐Tau (Thr205, 1:200). Sections were incubated with appropriate fluorescent secondary antibodies (Alexa Fluor 488/594, 1:500) and counterstained with DAPI. Images were captured using a confocal microscope (Leica Stellaris 5). Fluorescence intensity and colocalization were quantified using ImageJ or Imaris software.

Transmission Electron Microscopy (TEM): Hippocampal tissue was fixed in 2.5% glutaraldehyde, post‐fixed in 1% osmium tetroxide, dehydrated, and embedded in resin. Ultrathin sections (70 nm) were stained with uranyl acetate and lead citrate. TEM images were acquired using a JEOL 1400 microscope. Microtubule breakage ratio, synaptic cleft width, postsynaptic density (PSD) thickness, and synaptic density were quantified manually in multiple fields of view (n = 3 mice/group, 2 fields per mouse).

Western Blotting: Hippocampal tissues or cultured neurons were lysed in RIPA buffer with protease/phosphatase inhibitors. Protein concentration was measured using BCA assay. Equal amounts of protein (20–40 µg) were separated by SDS‐PAGE, transferred to PVDF membranes, and probed with primary antibodies: 14‐3‐3γ, Tau (total), p‐Tau (Thr205, Ser202/Thr205, Thr231, Ser396, Ser262), PSD95, SYP, β‐tubulin, or β‐actin (1:1000–1:2000). HRP‐conjugated secondary antibodies (1:5000) were used, and bands were detected using ECL chemiluminescence. Densitometry was performed using ImageJ, normalizing to β‐actin or β‐tubulin or GAPDH.

Co‐Immunoprecipitation (Co‐IP) and Mass Spectrometry: Hippocampal lysates (500 µg protein) were incubated with anti‐14‐3‐3γ or anti‐p‐Tau (Thr205) antibodies overnight at 4°C, followed by incubation with protein A/G agarose beads. Immunoprecipitates were washed, eluted, and analyzed by SDS‐PAGE and immunoblotting or subjected to LC‐MS/MS for identification of binding partners. Quantification of co‐IP signals was normalized to input controls.

#### 2.3.4 Primary Neuronal Culture and Transfection

HT22 cells were maintained in Dulbecco's Modified Eagle Medium (DMEM, high glucose) supplemented with 10% fetal bovine serum (FBS), 100 U/mL penicillin, and 100 µg/mL streptomycin. Cells were cultured at 37 °C in a humidified atmosphere containing 5% CO_2_ and passaged every 2–3 days before reaching 80%–90% confluence. Primary neurons were isolated from the hippocampi of embryonic day 18 (E18) C57BL/6 mouse embryos. Dissected hippocampi were digested with 0.25% trypsin‐EDTA for 15 min at 37 °C, followed by gentle trituration in Neurobasal medium supplemented with 2% B27, 0.5 mM L‐glutamine, and 1% penicillin/streptomycin. Cells were plated on poly‐D‐lysine–coated plates at a density of 1 × 10^5^ cells/cm^2^ and maintained at 37 °C in 5% CO_2_. Half of the medium was replaced every 3–4 days, and neurons were used for experiments after 10–14 days in vitro (DIV), when synaptic maturation is established. Cells were transfected with siRNA targeting 14‐3‐3γ (si14‐3‐3γ), non‐targeting siRNA (siNC), or plasmids expressing wild‐type Tau, phospho‐deficient (T205A), or phospho‐mimetic (T205E) Tau constructs using Lipofectamine 2000. For overexpression experiments, 14‐3‐3γ constructs were co‐transfected. Pharmacological treatments included CDK5 inhibitor (roscovitine, 10 µM), GSK3β inhibitor (LiCl, 10 mM), or PP2A activator (DT061, 1 µM) for 24 h.

#### 2.3.5 Adeno‐Associated Virus (AAV)‐Mediated Gene Manipulation

##### 2.3.5.1 AAV‐Mediated 14‐3‐3γ Overexpression

Neuron‐specific overexpression was achieved via stereotaxic injection of AAV vectors into hippocampal CA1 and dentate gyrus (AP: +2.06 mm, ML: ±1.5 mm, DV: −1.5/−2.0 mm). Vectors included pAAV‐hSyn‐Ywhag‐3xFLAG‐P2A‐EGFP‐WPRE (OE) or control vector pAAV‐hSyn‐3xFLAG‐P2A‐EGFP‐WPRE. Behavioral tests and tissue collection were performed 3–4 weeks post‐injection to allow sufficient expression.

Mice were anesthetized with isoflurane (1.5%–2.0%) and placed in a stereotaxic instrument (RWD Life Science, Shenzhen, China). A total of 1 µL of AAV vector was injected into each hemisphere at the following coordinates (relative to bregma): AP: +2.06 mm, ML: ±1.5 mm from midline, DV: −1.5 mm (CA1) and −2.0 mm (dentate gyrus). Injections were performed at a rate of 0.2 µL/min using a Hamilton syringe, and the needle was left in place for 5 min after injection to allow diffusion. Behavioral tests and tissue collection were performed 3–4 weeks post‐injection to allow sufficient expression.

##### 2.3.5.2 AAV‐Mediated TauT205E Overexpression

To constitutively mimic tau phosphorylation at Thr205, we generated AAV vectors expressing the phospho‐mimetic Tau mutant T205E. The following plasmids were used for AAV production: pAAV‐hSyn‐EGFP‐P2A‐MAPT(T205E)‐3xFLAG‐WPRE (referred to as AAV‐TauT205E) and pAAV‐hSyn‐EGFP‐P2A‐3xFLAG‐WPRE (empty vector control, referred to as AAV‐GFP). Stereotaxic injection procedures were identical to those described above for AAV‐14‐3‐3γ. For the loss‐of‐rescue experiment, mice received co‐injection of AAV‐TauT205E (or AAV‐GFP control) followed by FC‐A treatment and behavioral testing as described in the experimental timeline.

#### 2.3.6 Small Molecule Intervention

FC‐A (fusicoccin‐A, a 14‐3‐3 stabilizer) was administered intraperitoneally at 5 mg/kg, based on previous studies demonstrating its efficacy in stabilizing 14‐3‐3 protein‐protein interactions in neurological models [26, 38]. FC‐A was administered intraperitoneally 30 min before surgery. For inhibition studies, BV02 (14‐3‐3 inhibitor, 10 mg/kg) was co‐administered [39]. Behavioral testing and biochemical analyses were performed as described above.

#### 2.3.7 Molecular Dynamics Simulations of 14‐3‐3γ–p‐Tau Interaction

To elucidate the structural basis by which FC‐A stabilizes the 14‐3‐3γ–p‐Tau (Thr205) interaction, molecular docking and all‐atom molecular dynamics (MD) simulations were performed. A structural model of the 14‐3‐3γ–p‐Tau complex was first generated, followed by docking of FC‐A into the protein–protein interface based on known 14‐3‐3 binding modes. Two systems were constructed: the apo complex (14‐3‐3γ–p‐Tau) and the FC‐A–bound complex, representing the absence and presence of the small molecule stabilizer, respectively.

Both systems were parameterized using the AMBER force field for proteins and GAFF parameters for FC‐A, solvated in explicit water, and subjected to energy minimization, equilibration, and 500 ns production MD simulations under periodic boundary conditions. Temperature and pressure were maintained at physiological conditions, and long‐range electrostatics were treated using the particle mesh Ewald method.

Trajectory analyses were performed to assess conformational stability and interaction strength, including conformational ensemble comparison, structural fluctuation analysis, center‐of‐mass (COM) distance measurement between 14‐3‐3γ and p‐Tau, and binding free energy estimation using the MM/PBSA method. These analyses enabled quantitative comparison of interaction stability between the apo and FC‐A–bound systems.

#### 2.3.8 Statistical Analysis

Statistical analyses were performed using GraphPad Prism 9 (GraphPad Software, USA) and R software where applicable. Data distribution was assessed using the Shapiro–Wilk test, and potential outliers were evaluated based on predefined experimental criteria. Normally distributed continuous variables are presented as mean ± SEM unless otherwise specified, whereas non‐normally distributed variables are presented as median with interquartile range (IQR). Data transformation was performed when necessary to meet the assumptions of parametric analyses.

For comparisons between two independent groups, two‐sided unpaired Student's *t*‐tests were applied for normally distributed data, whereas nonparametric Mann–Whitney U tests were used when distributional assumptions were not satisfied. For comparisons involving more than two groups, one‐way analysis of variance (ANOVA) followed by appropriate post hoc multiple‐comparison tests was performed for normally distributed data, while Kruskal–Wallis tests with Dunn's multiple‐comparison tests were applied for nonparametric data. Paired analyses were performed using paired *t*‐tests or Wilcoxon signed‐rank tests when appropriate. Categorical variables were presented as counts and percentages. Pearson χ^2^ tests were used when assumptions were met; otherwise, Fisher's exact tests were applied for categorical variables with small expected cell counts. Given the limited number of POCD events, baseline comparisons were considered exploratory and were interpreted cautiously without adjustment for multiple comparisons. Multivariable regression analyses were adjusted for prespecified clinically relevant covariates. Model performance was evaluated using complementary measures of discrimination, calibration, reclassification, and clinical utility. Discrimination was assessed using ROC curves and AUCs, with between‐model comparisons performed using the DeLong test. Calibration was evaluated using calibration plots, while precision–recall curves were used to assess performance in the presence of imbalanced outcomes. The incremental value of plasma 14‐3‐3γ beyond clinical variables was assessed using NRI and IDI, and DCA was performed to evaluate net clinical benefit across a range of threshold probabilities. Given the limited number of outcome events, these predictive analyses were considered exploratory and were not interpreted as evidence of clinical validation. For analyses of the ADNI and PPMI cohorts, models evaluating CSF biomarkers, imaging biomarkers, and cognitive trajectories were adjusted for age, sex, education level, and APOE ε4 carrier status. For PET imaging analyses, all ROI‐based models used the same covariate adjustment strategy. For the prospective surgical cohort, regression models evaluating associations between plasma 14‐3‐3γ and postoperative neurocognitive outcomes were adjusted for clinically relevant confounders, including age, sex, and surgical type when applicable. Additional perioperative variables were considered according to the specific analysis objectives.

The specific statistical methods used for each experiment, including sample sizes, statistical tests, and data presentation formats, are described in the corresponding experimental sections and figure legends. All statistical tests were two‐sided, and *p* < 0.05 was considered statistically significant. Significance levels are indicated as *p < 0.05, **p < 0.01, ***p < 0.001, and ****p < 0.0001; ns indicates not significant. Sample sizes were determined based on previous studies using the tibial fracture model and our preliminary observations, with an estimated effect size corresponding to a 20–30% difference, a significance level of α = 0.05, and statistical power of 80%.

## 3 Results

### 3.1 CSF 14‐3‐3γ Identifies Neurodegeneration‐Enriched Cognitive Vulnerability and Predicts Subsequent Neurodegenerative Progression

#### 3.1.1 Identification of CSF Proteins Associated with Neurodegeneration‐Enriched Cognitive Vulnerability

**FIGURE 1:**
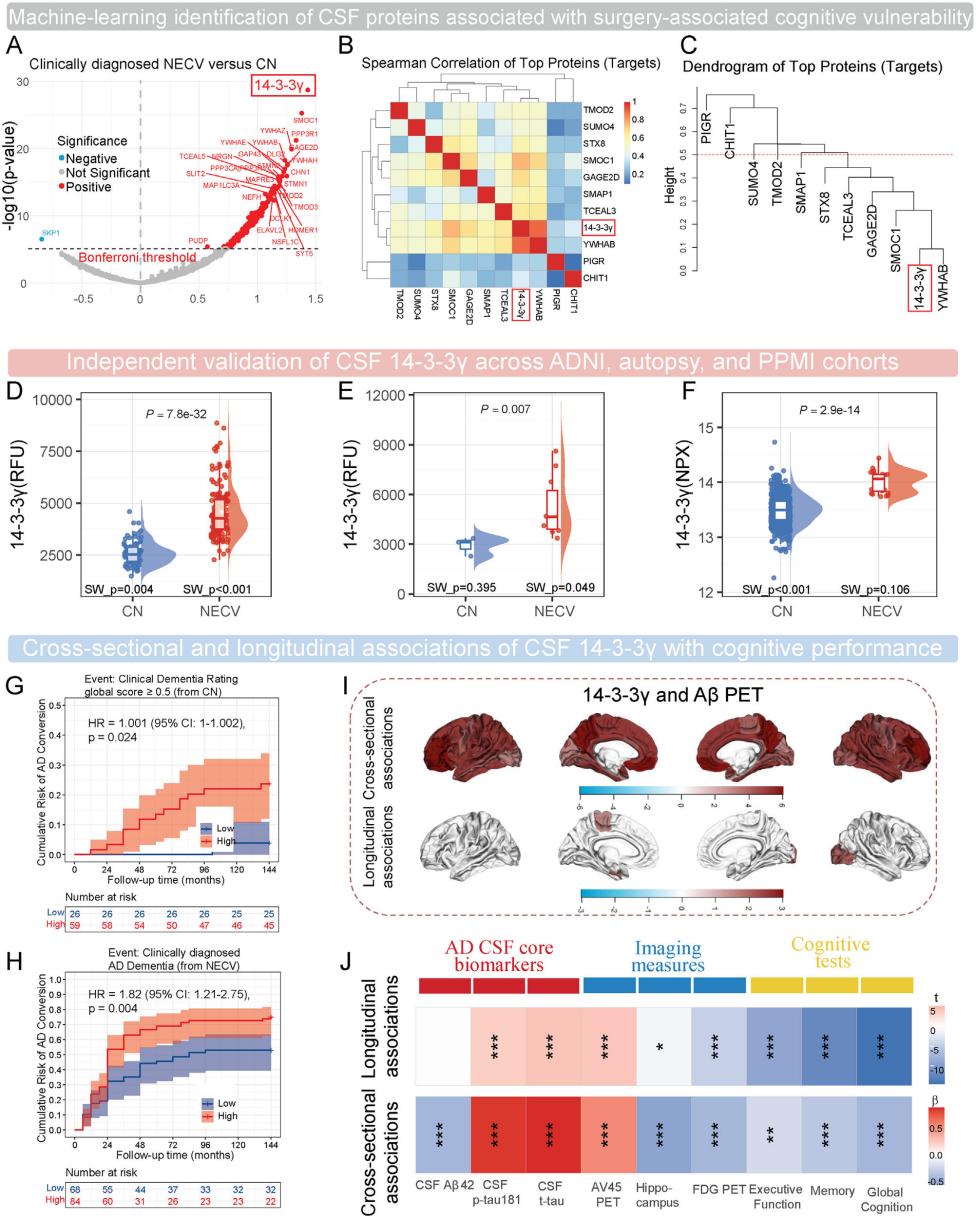
Multi‐cohort discovery and validation of CSF 14‐3‐3γ associated with cognitive vulnerability. (A) Volcano plot showing the beta estimate (x‐axis) and −log10(*p* value) (y‐axis) for differential protein analysis between individuals classified as neurodegeneration‐enriched cognitive vulnerability (NECV) and cognitively normal (CN) participants. Results of the multiple linear regressions adjusted for age, sex, education and APOE ε4 status are shown. *p* values were calculated under two‐sided tests and no multiple comparisons were applied. CSF proteins above the red horizontal line were significantly dysregulated between NECV and controls after Bonferroni corrections. (B) A heatmap shows the Spearman rank‐order correlations between each pair of the top‐11 candidate proteins for discriminating the clinically diagnosed NECV population from CN. (C) A dendrogram of hierarchical clustering based on calculated correlations. (D–F) Validation of CSF 14‐3‐3γ levels across different cohorts. (D) Differential expression of CSF 14‐3‐3γ between clinically diagnosed NECV and CN groups in the ADNI discovery cohort. (E) Autopsy neuropathological validation in the ADNI cohort, comparing 14‐3‐3γ levels between autopsy‐confirmed AD and non‐AD groups. (F) Independent external validation in the PPMI cohort, showing 14‐3‐3γ levels in NECV and CN groups. Statistics for (D–F): The raincloud plots illustrate the data distribution for each group. The boxes span the interquartile range (IQR) from the 25th percentile to the 75th percentile, with the central line representing the median. The whiskers extend to the smallest and largest values within 1.5 times the IQR. The cloud to the right of each box displays the distribution density. Statistical significance was assessed using a two‐sided Mann–Whitney U test. (G) Cumulative risk of clinical progression from cognitively normal (CN) to the incident prodromal stage of AD (defined by a Clinical Dementia Rating [CDR] global score ≥ 0.5). (H) Cumulative risk of progression from neurodegeneration‐enriched cognitive vulnerability (NECV) to incident AD dementia. The curves are visualized for individuals with low (blue line) and high (red line) baseline CSF 14‐3‐3γ levels. Shaded regions represent standard errors derived from the cumulative incidence proportions. Cox proportional‐hazard models were used to estimate the association between baseline 14‐3‐3γ levels and the risk of conversion, with hazard ratios (HRs), 95% confidence intervals (CIs) and *p* values calculated after adjusting for age, sex, years of education and APOE ε4 status. *p* values were determined using two‐sided tests and no multiple comparisons were applied. (I) Associations between CSF 14‐3‐3γ levels and regional amyloid deposition assessed by florbetapir ([18F]AV45) PET. Regional AV45 SUVR values were analyzed using multivariable linear regression models adjusted for age, sex, education, and APOE ε4 status. Cortical regions were defined according to the Desikan–Killiany atlas. Statistical significance was determined after false discovery rate correction for multiple comparisons. Brain maps were generated using a visualization framework adapted from Janssen et al. (2025) [49]. All results shown are derived from independent analyses in the current study. (J) Heat maps illustrate the relationships between 14‐3‐3γ and cognitive endophenotypes at baseline (top) and over time (bottom). Colors correspond to beta coefficients, while asterisks denote levels of statistical significance following Bonferroni correction (***p < 0.001, **p < 0.01, *p < 0.05, no symbols *p* ≥ 0.05).

To systematically identify cerebrospinal fluid (CSF) proteins associated with early cognitive decline, we performed multi‐cohort proteomic profiling comparing individuals with neurodegeneration‐enriched cognitive vulnerability (NECV) and cognitively normal (CN) controls. Differential expression analysis in the discovery cohort revealed a subset of proteins significantly dysregulated in NECV, among which 14‐3‐3γ (YWHAG) ranked among the top candidates after stringent Bonferroni correction (Figure 1A). Network‐based correlation analysis further demonstrated that 14‐3‐3γ occupied a central position within the co‐expression structure of top‐ranked proteins, suggesting coordinated regulation within disease‐relevant pathways (Figure 1B,C).

#### 3.1.2 Independent Validation of CSF 14‐3‐3y Across ADNI, Autopsy, and PPMI Cohorts

We next validated these findings across independent cohorts. CSF 14‐3‐3γ levels were significantly elevated in participants classified as NECV compared with CN controls (*p* = 7.8 × 10^−^^32^), further increased in autopsy‐confirmed Alzheimer's disease (AD) cases (*p* = 0.007), and replicated in the external PPMI cohort (*p* = 2.9 × 10^−^^14^) (Figure 1D–F), demonstrating consistent cross‐cohort reproducibility.

#### 3.1.3 Cross‐Sectional and Longitudinal Associations of CSF 14‐3‐3y with Cognitive Performance

Longitudinal survival analyses revealed that elevated baseline CSF 14‐3‐3γ was associated with increased risk of disease progression. Specifically, higher 14‐3‐3γ levels predicted conversion from CN to prodromal AD (hazard ratio [HR] = 1.001, 95% CI: 1.001–1.002, *p* = 0.024) and from NECV to AD dementia (HR = 1.82, 95% CI: 1.21–2.75, *p* = 0.004) (Figure 1G,H), indicating its prognostic value for clinical deterioration. We further examined the relationship between CSF 14‐3‐3γ and core AD biomarkers, neuroimaging measures, and cognitive performance. Cross‐sectionally, higher CSF 14‐3‐3γ levels were strongly associated with hallmark AD pathology, including lower CSF Aβ42 (β = −0.451, *p* = 1.15 × 10^−^^1^ ^5^) and higher CSF p‐tau181 (β = 0.861, *p* = 5.26 × 10^−^^7^ ^1^) and t‐tau (β = 0.885, *p* = 6.47 × 10^−^^7^ ^4^). Consistently, 14‐3‐3γ was positively associated with cortical amyloid burden measured by AV45 PET SUVR (β = 0.536, *p* = 9.69 × 10^−^^1^ ^6^), and negatively associated with hippocampal volume (β = −0.565, *p* = 1.05 × 10^−^^7^) and cerebral glucose metabolism assessed by FDG PET (β = −0.444, *p* = 5.21 × 10^−^^9^). At the cognitive level, elevated 14‐3‐3γ correlated with worse executive function (β = −0.232, *p* = 3.79 × 10^−^^3^), memory (β = −0.336, *p* = 2.12 × 10^−^^4^), and global cognition (β = −0.450, *p* = 2.32 × 10^−^^1^ ^1^) (Figure 1J; Table S3, Supporting File 2). Regionally, CSF 14‐3‐3γ showed widespread positive associations with cortical amyloid deposition across nearly all examined brain regions (Table S2, Supporting File 2), including frontal, temporal, parietal, and limbic cortices (β range ≈ 0.34–0.62, all Bonferroni‐corrected *p* < 0.01). Notably, strong effects were observed in disease‐relevant regions such as the precuneus, medial orbitofrontal cortex, and posterior cingulate cortex, supporting a global relationship between 14‐3‐3γ and amyloid pathology (Figure 1I). Longitudinal analyses further demonstrated that baseline CSF 14‐3‐3γ predicts progressive neurodegeneration and cognitive decline. Higher 14‐3‐3γ levels were associated with faster increases in CSF p‐tau181 (β = 1.91 × 10^−^^5^, *p* = 7.57 × 10^−^^5^) and t‐tau (β = 2.26 × 10^−^^4^, *p* = 3.24 × 10^−^^6^), as well as accelerated accumulation of amyloid burden (AV45 PET: β = 2.76 × 10^−^^7^, *p* = 7.46 × 10^−^^6^). In parallel, elevated 14‐3‐3γ predicted longitudinal reductions in FDG PET metabolism (β = −4.90 × 10^−^^7^, *p* = 3.32 × 10^−^^7^) and progressive decline in executive function (β = −2.87 × 10^−^^6^, *p* = 5.27 × 10^−^^2^ ^0^), memory (β = −3.68 × 10^−^^6^, *p* = 3.26 × 10^−^^3^ ^3^), and global cognition (β = −2.18 × 10^−^^5^, *p* = 9.44 × 10^−^^4^ ^6^) (Figure 1J; Table S4, Supporting File 2). In contrast, longitudinal associations with hippocampal volume and CSF Aβ42 were modest or not statistically significant. At the regional level, longitudinal associations between CSF 14‐3‐3γ and cortical amyloid progression were relatively limited (Table S3, Supporting File 2), with only a few regions such as the entorhinal cortex (left: β = 3.45 × 10^−^^4^, *p* = 0.023) and lateral occipital cortex (right: β = 2.10 × 10^−^^4^, *p* = 0.041) showing nominal significance, suggesting that 14‐3‐3γ may more strongly reflect baseline amyloid burden rather than its regional accumulation dynamics.

Collectively, these results demonstrate that CSF 14‐3‐3γ is not only a sensitive marker of early cognitive impairment but also a consistent predictor of molecular pathology, neurodegeneration, and longitudinal cognitive decline, supporting its potential as an integrated biomarker of disease vulnerability and progression. While NECV does not directly represent POCD, the finding that CSF 14‐3‐3γ levels predict both NECV status and subsequent AD dementia conversion suggests that this protein may reflect a common molecular vulnerability shared by perioperative cognitive stress and neurodegenerative disease progression.

### 3.2 Early Postoperative Plasma 14‐3‐3γ Is Associated with Perioperative Neurocognitive Outcomes

**FIGURE 2:**
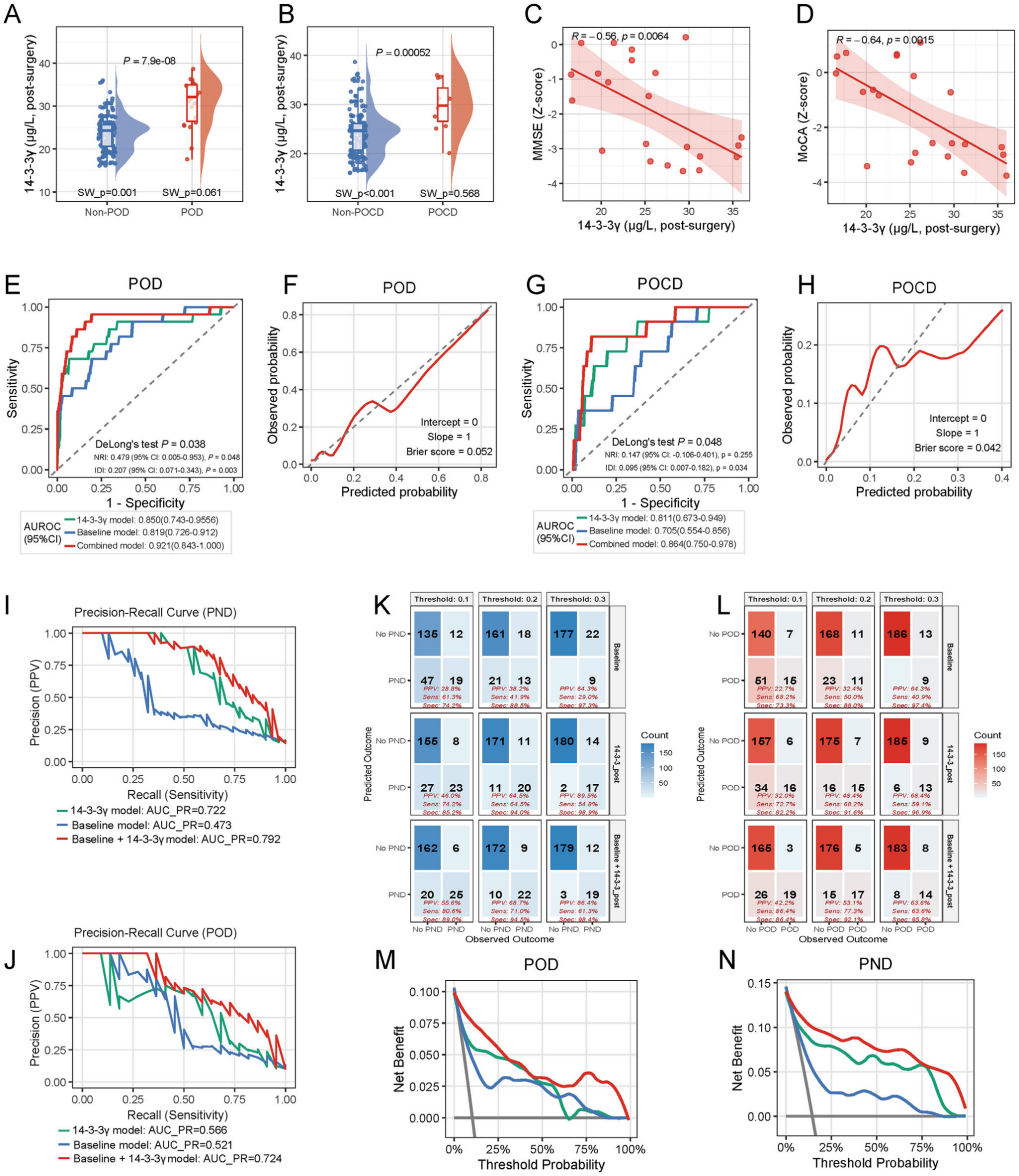
Early postoperative plasma 14‐3‐3γ was associated with subsequent POD and POCD in the prospective surgical cohort. (A,B) Plasma 14‐3‐3γ in a prospective surgical cohort (N = 213). Postoperative plasma 14‐3‐3γ levels in patients with and without postoperative delirium (POD) (A) or postoperative cognitive dysfunction (POCD) (B). Data are shown as violin plots with overlaid boxplots (median ± IQR). Statistical significance was assessed using a two‐sided Mann–Whitney U test. (C,D) Linear correlation analyses between postoperative plasma 14‐3‐3γ levels and cognitive performance (MMSE, C; MoCA, D) in the 22 matched patients (11 POCD and 11 non‐POCD). Pearson correlation coefficients (R) and two‐sided *p* values are reported. (E–L) Multivariable logistic regression models incorporating plasma 14‐3‐3γ predict risk of POD and POCD in a prospective surgical cohort (N = 213). (E,G) Receiver operating characteristic (ROC) curves for predicting POD (E) and POCD (G) using plasma 14‐3‐3γ–based models. The AUROC (95% CI) for POD was 0.850 (0.743–0.956) for the 14‐3‐3γ model, 0.819 (0.726–0.912) for the baseline model, and 0.921 (0.843–1.000) for the combined model. For POCD, AUROC values were 0.811 (0.673–0.949), 0.705 (0.554–0.856), and 0.864 (0.750–0.978), respectively. DeLong's test for comparison between models yielded *p* = 0.038 (POD) and *p* = 0.048 (POCD). Net reclassification improvement (NRI) and integrated discrimination improvement (IDI) statistics are shown in each panel. (F,H) Calibration curves showing observed versus predicted probabilities for POD (F) and POCD (H) in the matched patient cohort. The slope and intercept indicate agreement between predicted and observed outcomes. Brier scores were 0.052 (POD) and 0.042 (POCD), indicating acceptable model calibration. (I–L) Additional evaluation of model performance using precision‐recall analysis and threshold‐based classification metrics under imbalanced outcome conditions. (I) Precision‐recall curves for predicting perioperative neurocognitive disorders (PND), defined as the occurrence of postoperative delirium (POD) or postoperative cognitive dysfunction/delayed neurocognitive recovery (POCD/dNCR). The area under the precision‐recall curve (AUPRC) was 0.722 for the plasma 14‐3‐3γ model, 0.473 for the baseline clinical model, and 0.792 for the combined model incorporating plasma 14‐3‐3γ. (J) Precision‐recall curves for POD prediction. The corresponding AUPRC values were 0.566, 0.521, and 0.724 for the plasma 14‐3‐3γ model, baseline clinical model, and combined model, respectively. (K) Confusion matrices of the combined POD prediction model (baseline clinical variables plus plasma 14‐3‐3γ) at representative probability thresholds (0.1, 0.2, and 0.3). Each matrix displays the numbers of true positive, false positive, true negative, and false negative classifications. Positive predictive value (PPV), sensitivity, and specificity are indicated within each panel. (L) Classification performance of the combined PND model across different probability thresholds. (M,N) Decision curve analysis (DCA) evaluating the potential clinical utility of plasma 14‐3‐3γ–based models for predicting POD (M) and PND (N). The net benefit of the plasma 14‐3‐3γ‐only model, baseline clinical model, and combined model incorporating plasma 14‐3‐3γ is shown across a range of threshold probabilities. The “treat‐all” and “treat‐none” strategies are shown as reference lines. The combined model provided greater net benefit than the individual models across a range of clinically relevant threshold probabilities. DCA was not performed for POCD because of the limited number of events (n = 11), which could result in unstable and difficult‐to‐interpret decision curves. Positive predictive value (PPV), sensitivity, specificity, and negative predictive value (NPV) are shown for thresholds of 0.1, 0.2, and 0.3. Abbreviations: PLT, platelet count; PSI, perioperative stress index; TG, triglycerides; CAD, coronary artery disease; CRP, C‐reactive protein; BMI, body mass index; AUROC, area under the receiver operating characteristic curve; NRI, net reclassification improvement; IDI, integrated discrimination improvement.

To evaluate the clinical relevance of 14‐3‐3γ in POCD, we analyzed a prospective surgical cohort recruited at our center (registration number: ChiCTR2500096293). Baseline demographic, clinical, and intraoperative characteristics of the 213 enrolled patients are presented in Table 1 (stratified by POCD status) and Table 2 (stratified by POD status). Baseline characteristics were compared descriptively between outcome groups, and these comparisons should be interpreted cautiously because of the limited number of outcome events. Among the 213 enrolled patients, 22 (10.3%) developed postoperative delirium (POD) as assessed by CAM‐ICU within the first 7 postoperative days. Postoperative cognitive dysfunction (POCD), defined by a composite Z‐score ≤ ‐1.96 at postoperative day 7, occurred in 11 patients (5.2%). According to the 2018 Nomenclature Consensus Working Group recommendations, cognitive decline within 30 days post‐surgery is more precisely termed delayed neurocognitive recovery (dNCR); in our cohort, all 11 POCD cases met criteria for dNCR. No patients exhibited persistent cognitive decline beyond 30 days in our follow‐up period. The incidence rates are consistent with previously reported ranges for major surgery in elderly populations [1]. In this prospective cohort, postoperative plasma 14‐3‐3γ levels were significantly higher in patients with postoperative delirium (POD) and POCD compared to controls (Figure 2A,B). Importantly, plasma 14‐3‐3γ levels negatively correlated with cognitive performance scores, including MMSE and MoCA (Figure 2C,D), indicating that elevated levels reflect worse cognitive outcomes.

Integration of plasma 14‐3‐3γ into multivariable logistic regression models substantially improved predictive performance for both POD and POCD. Combined models incorporating 14‐3‐3γ and clinical variables achieved superior discrimination compared to baseline models alone (Figure 2E–L). To determine whether plasma 14‐3‐3γ provided incremental predictive value beyond conventional clinical variables, we compared baseline clinical models with models incorporating plasma 14‐3‐3γ. For POD prediction, the baseline model achieved an AUC of 0.819 (95% CI, 0.726–0.912), which increased to 0.921 (95% CI, 0.843–1.000) after incorporation of plasma 14‐3‐3γ (DeLong test, *p* = 0.038). Addition of plasma 14‐3‐3γ significantly improved risk reclassification (NRI = 0.479, 95% CI 0.005–0.953, *p* = 0.048) and discrimination (IDI = 0.207, 95% CI 0.071–0.343, *p* = 0.003) (Figure 2E). The combined model demonstrated good calibration (Brier score = 0.052) (Figure 2F). For POCD prediction, incorporation of plasma 14‐3‐3γ improved the AUC from 0.705 (95% CI, 0.554–0.856) for the baseline model to 0.864 (95% CI, 0.750–0.978) for the combined model (DeLong test, *p* = 0.048). The combined model showed improved discrimination based on IDI (0.095, 95% CI 0.007–0.182, *p* = 0.034), although the NRI improvement did not reach statistical significance (NRI = 0.147, 95% CI ‐0.100–0.401, *p* = 0.255) (Figure 2G). Calibration analysis indicated good agreement between predicted and observed risks (Brier score = 0.042) (Figure 2H).

To further evaluate the potential clinical utility of plasma 14‐3‐3γ under conditions of outcome imbalance, we performed decision curve analysis (DCA), precision‐recall analyses, and threshold‐based classification assessments. DCA demonstrated that the combined models incorporating plasma 14‐3‐3γ provided greater net benefit than the baseline clinical models and the plasma 14‐3‐3γ‐only models across a range of threshold probabilities for both POD and PND (Figure 2M,N). These findings suggest a potential incremental net benefit of incorporating plasma 14‐3‐3γ beyond conventional clinical variables in this exploratory cohort. However, given the limited number of outcome events, these decision‐analytic findings should be considered exploratory rather than evidence of established clinical utility. Because only 11 patients developed POCD, DCA was not performed for POCD separately because of the potential instability and limited interpretability of decision curves with such a small number of events. We therefore additionally evaluated a clinically relevant composite outcome of perioperative neurocognitive disorders (PND), defined as the occurrence of either POD or POCD. For PND prediction, the combined model incorporating baseline clinical variables and postoperative plasma 14‐3‐3γ demonstrated superior performance compared with the baseline model and plasma 14‐3‐3γ alone, with AUPRC values of 0.792, 0.473, and 0.722, respectively (Figure 2I). At clinically relevant probability thresholds, the combined model maintained favorable classification performance. At a threshold of 0.2, the combined model achieved a sensitivity of 71.0%, specificity of 94.5%, PPV of 68.7%, and NPV of 95.0%, correctly identifying 22 of 31 patients with PND while generating 10 false‐positive classifications (Figure 2K,L). Increasing the threshold to 0.3 improved PPV to 86.4% and specificity to 98.4%, whereas a lower threshold of 0.1 provided higher sensitivity (80.6%) at the expense of more false‐positive predictions. For POD prediction, precision‐recall analysis showed that the combined model also improved discrimination compared with baseline clinical variables, with AUPRC values of 0.724 versus 0.521, respectively (Figure 2J).

### 3.3 Surgery Induces Cognitive Impairment Accompanied by Hippocampal 14‐3‐3γ Loss and Preferential Tau Thr205 Hyperphosphorylation

#### 3.3.1 Anesthesia and Surgery Induce Perioperative Cognitive Impairment in Mice

**FIGURE 3:**
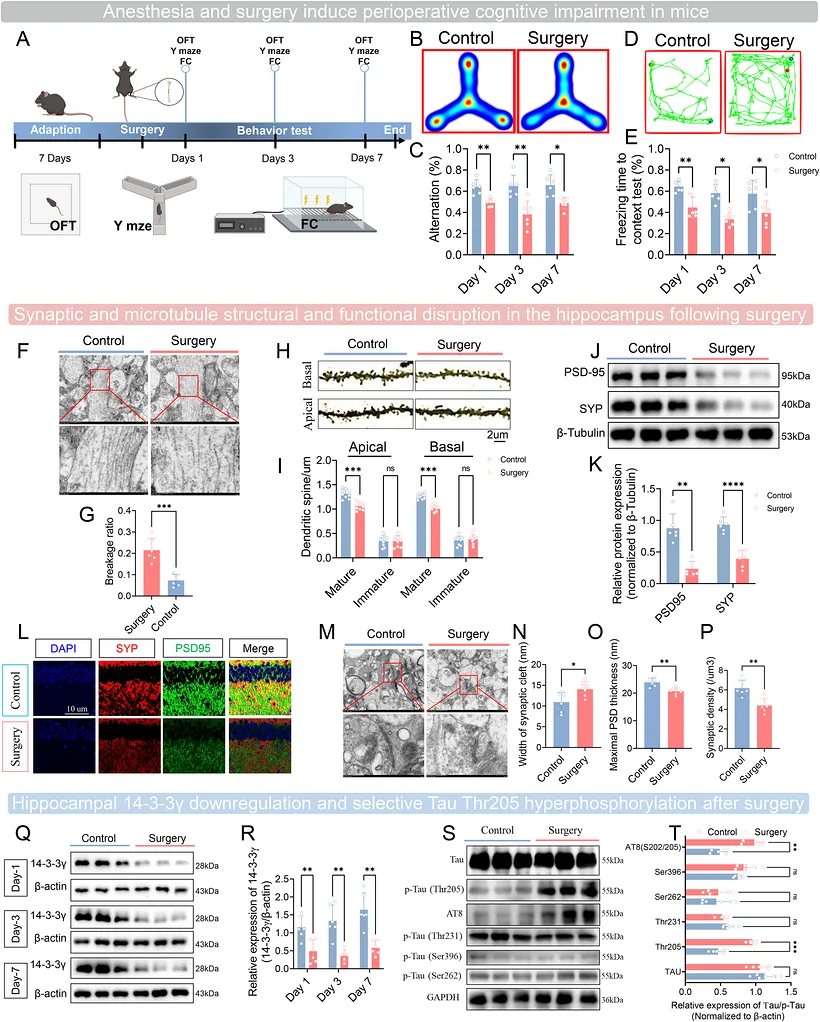
Anesthesia and surgery induce cognitive impairment accompanied by hippocampal 14‐3‐3γ loss, microtubule disruption, and Tau Thr205 hyperphosphorylation. (A–E) Anesthesia and tibial fracture surgery induce perioperative cognitive impairment in 3‐month‐old C57BL/6 male mice. (A) Experimental timeline and behavioral workflow. Mice underwent 7 days of adaptation prior to tibial fracture with intramedullary fixation (surgery) or sham procedures (control). Behavioral tests included open field test (OFT) for general locomotion, Y‐maze for spontaneous alternation, and fear conditioning (FC) for hippocampus‐dependent contextual and cued memory. Cognitive assessments were performed on postoperative days 1, 3, and 7. The schematic illustration in panel A was created using BioRender (https://www.biorender.com/), with graphical elements obtained from the BioRender library. (B) Representative Y‐maze heatmaps for control and surgery mice, illustrating reduced exploratory alternation in the surgery group. (C) Quantification of spontaneous alternation in the Y‐maze at postoperative days 1, 3, and 7. Surgery mice showed significantly lower alternation percentages compared with controls (*p < 0.05; two‐sided unpaired *t*‐test; n = 6 mice/group). (D) Representative trajectories of mice during the contextual fear conditioning test. Surgery mice showed altered exploration patterns compared with controls, consistent with impaired hippocampus‐dependent memory. (E) Freezing behavior during the contextual phase of the FC test at postoperative days 1, 3, and 7. Surgery mice exhibited significantly reduced freezing time compared with controls (*p < 0.05; two‐sided unpaired *t*‐test; n = 6 mice/group). Data are presented as mean ± SEM. OFT and cued FC tests are shown in Supporting Materials to demonstrate preserved locomotor activity and cued memory performance. F–P Surgery induces synaptic and microtubule structural and functional disruption in the hippocampus. (F) Representative transmission electron microscopy (TEM) images of hippocampal microtubules in control and surgery mice. Insets show higher‐magnification views highlighting microtubule organization and fragmentation. (G) Quantification of microtubule breakage ratio. Microtubule breakage was defined as the proportion of disrupted microtubules relative to the total number of microtubules within the same field of view. Microtubules were manually counted, and the number of visibly broken microtubules was divided by the total microtubule count per image. Surgery significantly increased microtubule breakage compared with controls. (H) Representative Golgi staining images of hippocampal neurons showing dendritic spines in apical and basal dendrites in control and surgery mice. Scale bar, 2 µm. (I) Quantification of dendritic spine density, including mature and immature spines in apical and basal dendrites. Surgery reduced mature spine density without significantly affecting immature spines. (J) Representative immunoblot images of synaptic proteins PSD95 and synaptophysin (SYP) in the hippocampus, with β‐tubulin as a loading control. (K) Quantification of PSD95 and SYP protein expression normalized to β‐tubulin. Surgery significantly decreased synaptic protein levels compared with controls. (L) Representative immunofluorescence images of hippocampal sections stained for PSD95 (green), SYP (red), and nuclei (DAPI, blue). Merged images show reduced synaptic signal intensity in surgery mice. Scale bar, 10 µm. (M) Representative TEM images of synaptic ultrastructure in the hippocampus. Insets show higher‐magnification views of synaptic contacts. (N–P) Quantitative analysis of synaptic ultrastructural parameters, including synaptic cleft width (N) postsynaptic density (PSD) thickness (O) and synaptic density (P). Surgery induced significant alterations in synaptic architecture, characterized by increased synaptic cleft width, reduced PSD thickness, and decreased synaptic density. Data are presented as mean ± SEM. For TEM‐based analyses (F,G,M–P), n = 3 mice per group, with 2 independent fields of view analyzed per mouse. For dendritic spine analysis (H,I), n = 3 mice per group, with 3 neurons analyzed per mouse. For immunoblot analysis (J,K), n = 6 mice per group. Statistical comparisons were performed using two‐sided unpaired *t*‐tests. Significance levels are indicated as *p < 0.05, **p < 0.01, ***p < 0.001, and ****p < 0.0001; ns, not significant. (Q–T) Surgery induces hippocampal 14‐3‐3γ downregulation and selective Tau Thr205 hyperphosphorylation. (Q) Representative immunoblot images of 14‐3‐3γ expression in the hippocampus at postoperative days 1, 3, and 7 in control and surgery mice, with β‐actin as a loading control. (R) Quantification of 14‐3‐3γ protein levels normalized to β‐actin at postoperative days 1, 3, and 7, showing a significant reduction following surgery. (S) Representative immunoblot images of total Tau and site‐specific phosphorylated Tau in the hippocampus at postoperative day 7, including p‐Tau (Thr205), AT8 (Ser202/Thr205), p‐Tau (Thr231), p‐Tau (Ser396), and p‐Tau (Ser262), with GAPDH as a loading control. (T) Quantification of total Tau and phosphorylated Tau levels normalized to β‐actin. Surgery selectively increased Tau phosphorylation at Thr205, whereas no significant changes were observed at other phosphorylation sites. Data are presented as mean ± SEM. For immunoblot analyses, n = 6 mice per group. Statistical comparisons were performed using two‐sided unpaired *t*‐tests. Significance levels are indicated as *p < 0.05, **p < 0.01, ***p < 0.001, and ****p < 0.0001; ns, not significant.

To investigate mechanistic links, we established a murine model of perioperative neurocognitive disorders. Mice subjected to anesthesia and tibial fracture surgery exhibited significant impairments in hippocampus‐dependent memory, as evidenced by reduced spontaneous alternation in the Y‐maze and decreased contextual freezing (Figure 3A–E), while locomotor activity and cued memory remained intact (Figure S1, Supporting File 2).

#### 3.3.2 Synaptic and Microtubule Structural and Functional Disruption in the Hippocampus Following Surgery

At the structural level, surgery induced pronounced microtubule disruption and synaptic degeneration. Electron microscopy revealed increased microtubule fragmentation (Figure 3F,G), while Golgi staining showed reduced dendritic spine density (Figure 3H,I). Synaptic protein levels (PSD95 and synaptophysin) were significantly decreased, accompanied by ultrastructural synaptic defects (Figure 3J–P).

#### 3.3.3 Hippocampal 14‐3‐3γ Downregulation and Preferential Tau Thr205 Hyperphosphorylation after Surgery

Biochemically, surgery resulted in a marked reduction of hippocampal 14‐3‐3γ expression (Figure 3Q,R). Importantly, analysis of other 14‐3‐3 isoforms revealed no significant or consistent changes in expression following surgery, including 14‐3‐3η, 14‐3‐3σ, 14‐3‐3ζ/δ, 14‐3‐3ε, and 14‐3‐3α/β (Figure S2, Supporting File 2), supporting a selective vulnerability of the 14‐3‐3γ isoform in the perioperative brain. Tau protein normally stabilizes microtubules, and its hyperphosphorylation reduces this stabilizing function, leading to microtubule fragmentation. Conversely, microtubule disruption can activate stress signaling pathways that further promote tau phosphorylation. Concomitantly, Tau phosphorylation was selectively increased at Thr205 and AT8 (Ser202/Thr205), without significant changes at other phosphorylation sites; Tau Thr205 hyperphosphorylation was detectable and pronounced at day 7 (Figure 3S,T). These findings identify a molecular signature linking 14‐3‐3γ loss to Tau hyperphosphorylation, with Thr205 showing the most prominent increase among the examined phosphorylation sites at postoperative day 7. Among the examined sites, phosphorylation at Thr205 (including AT8 immunoreactivity reflecting Ser202/Thr205 phosphorylation) showed the most robust increase following surgery, whereas other assessed sites did not exhibit significant changes at postoperative day 7 (Figure 3S,T). This does not preclude the possibility that other sites become phosphorylated at earlier or later time points; indeed, anesthesia‐induced hypothermia has been shown to promote phosphorylation at Thr212, Ser262, and Ser404 [40]. However, the sustained elevation of Thr205 phosphorylation through postoperative day 7 suggests that this site may be particularly relevant for the persistent cognitive deficits observed in our model.

Notably, while hippocampal 14‐3‐3γ was markedly reduced after surgery, its levels were significantly elevated in both serum and cerebrospinal fluid in mice and in clinical cohorts, indicating a compartment‐specific divergence between central and peripheral expression (Figure S3, Supporting File 2). This dissociation suggests a hypothesis‐generating observation: the elevated extracellular (plasma/CSF) 14‐3‐3γ and reduced intracellular (hippocampal) 14‐3‐3γ following surgery may reflect distinct biological processes.

### 3.4 Neuron‐Specific Overexpression of 14‐3‐3γ Rescues Cognitive and Synaptic Deficits

**FIGURE 4:**
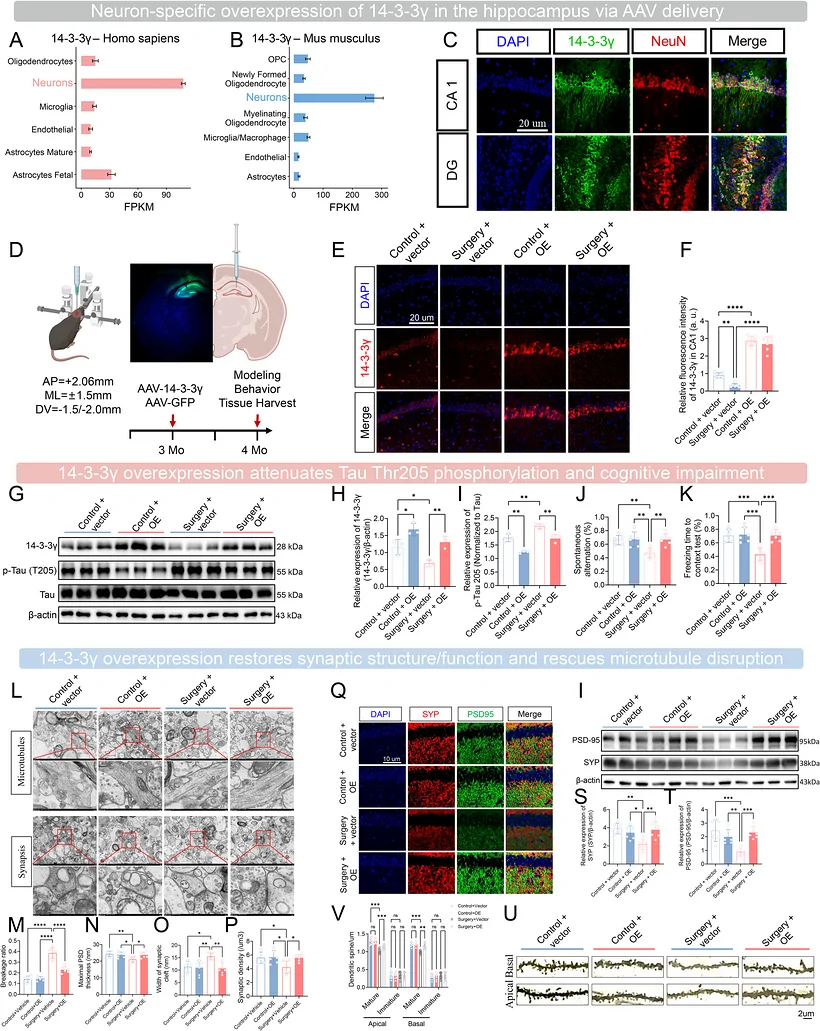
Neuron‐specific overexpression of 14‐3‐3γ rescues perioperative cognitive dysfunction by suppressing Tau Thr205 phosphorylation and restoring synaptic and microtubule integrity. (A–F) Neuron‐specific overexpression of 14‐3‐3γ in the hippocampus via AAV delivery. (A,B) Single‐cell sequencing analysis of 14‐3‐3γ across CNS cell types of both human and mouse origin (https://www.brainrnaseq.org/). (C) Representative immunofluorescence images of hippocampal regions (CA1 and dentate gyrus, DG) showing co‐localization of 14‐3‐3γ (green) with the neuronal marker NeuN (red), confirming neuronal enrichment. Nuclei are stained with DAPI (blue). Scale bar, 20 µm. (D) Schematic of adeno‐associated virus (AAV)–mediated hippocampal delivery of 14‐3‐3γ. Mice received stereotaxic injection of AAV vectors (AP: +2.06 mm, ML: ±1.5 mm, DV: −1.5/−2.0 mm), followed by surgery, behavioral testing, and tissue collection. Overexpression (OE) vector: pAAV‐hSyn‐Ywhag‐3xFLAG‐P2A‐EGFP‐WPRE; control vector: pAAV‐hSyn‐3xFLAG‐P2A‐EGFP‐WPRE. The schematic illustration in panel D was created using BioRender (https://www.biorender.com), with graphical elements from the BioRender platform. (E) Representative fluorescence images showing AAV‐mediated expression of 14‐3‐3γ in the hippocampus across experimental groups. (F) Quantification of 14‐3‐3γ fluorescence intensity in the CA1 region, confirming efficient overexpression in AAV‐treated mice. (G–K) AAV‐mediated 14‐3‐3γ overexpression attenuates Tau Thr205 phosphorylation and cognitive impairment. (G) Representative immunoblot images of 14‐3‐3γ, phosphorylated Tau (Thr205), and total Tau in hippocampal tissue. (H) Quantification of 14‐3‐3γ protein expression. (I) Quantification of p‐Tau (Thr205) levels normalized to total Tau, showing that 14‐3‐3γ overexpression reduces surgery‐induced Tau Thr205 hyperphosphorylation. (J,K) Behavioral assessments, including spontaneous alternation in the Y‐maze (J) and contextual freezing in fear conditioning (K). AAV‐mediated 14‐3‐3γ overexpression ameliorates surgery‐induced cognitive deficits. (L‐U) AAV‐mediated 14‐3‐3γ overexpression restores synaptic structure/function and rescues microtubule disruption. (L) Representative TEM images of hippocampal microtubules and synaptic ultrastructure across experimental groups, showing reduced microtubule fragmentation and improved synaptic integrity following 14‐3‐3γ overexpression. (M–P) Quantification of microtubule breakage ratio (M) and synaptic ultrastructural parameters, including postsynaptic density (PSD) thickness (N) synaptic cleft width (O) and synaptic density (P). (Q) Representative immunofluorescence images of synaptic markers PSD95 (green) and synaptophysin (SYP, red) with DAPI (blue), showing restoration of synaptic signal intensity upon 14‐3‐3γ overexpression. (R) Representative immunoblot images of PSD95 and SYP in hippocampal tissue. (S,T) Quantification of synaptic protein expression normalized to β‐actin. (U) Representative Golgi staining images of dendritic spines in apical and basal dendrites. (V) Quantification of dendritic spine density, including mature and immature spines in apical and basal compartments, showing rescue of spine loss by 14‐3‐3γ overexpression. Data are presented as mean ± SEM. For immunoblot and behavioral analyses, n = 6 mice per group. For TEM analyses, n = 3 mice per group with 2 independent fields of view per mouse, effects of surgery and 14‐3‐3γ overexpression were analyzed by two‐way ANOVA followed by an adjusted post hoc test. For dendritic spine analysis, n = 3 mice per group with 3 neurons analyzed per mouse. Statistical comparisons were performed using two‐sided unpaired *t*‐tests. Significance levels are indicated as *p < 0.05, **p < 0.01, ***p < 0.001, and ****p < 0.0001; ns, not significant.

To determine causality, we overexpressed 14‐3‐3γ in hippocampal neurons via AAV delivery. Efficient neuronal targeting was confirmed by colocalization with NeuN and consistent expression in hippocampal regions (Figure 4A–F). Functionally, 14‐3‐3γ overexpression significantly attenuated surgery‐induced Tau Thr205 hyperphosphorylation (Figure 4G–I). Behavioral assessments showed marked improvement in working memory and contextual fear memory (Figure 4J,K), indicating rescue of cognitive deficits. At the structural level, 14‐3‐3γ overexpression restored microtubule integrity and synaptic architecture, as demonstrated by reduced microtubule breakage, normalization of synaptic ultrastructure, and recovery of dendritic spine density (Figure 4L–V). Synaptic protein expression was also restored (Figure 4Q–T). These results establish a protective role for 14‐3‐3γ in maintaining neuronal structure and function under perioperative stress.

### 3.5 Loss of 14‐3‐3γ Is Associated with Altered Kinase–Phosphatase Signaling and Increased Tau Thr205 Phosphorylation

**FIGURE 5:**
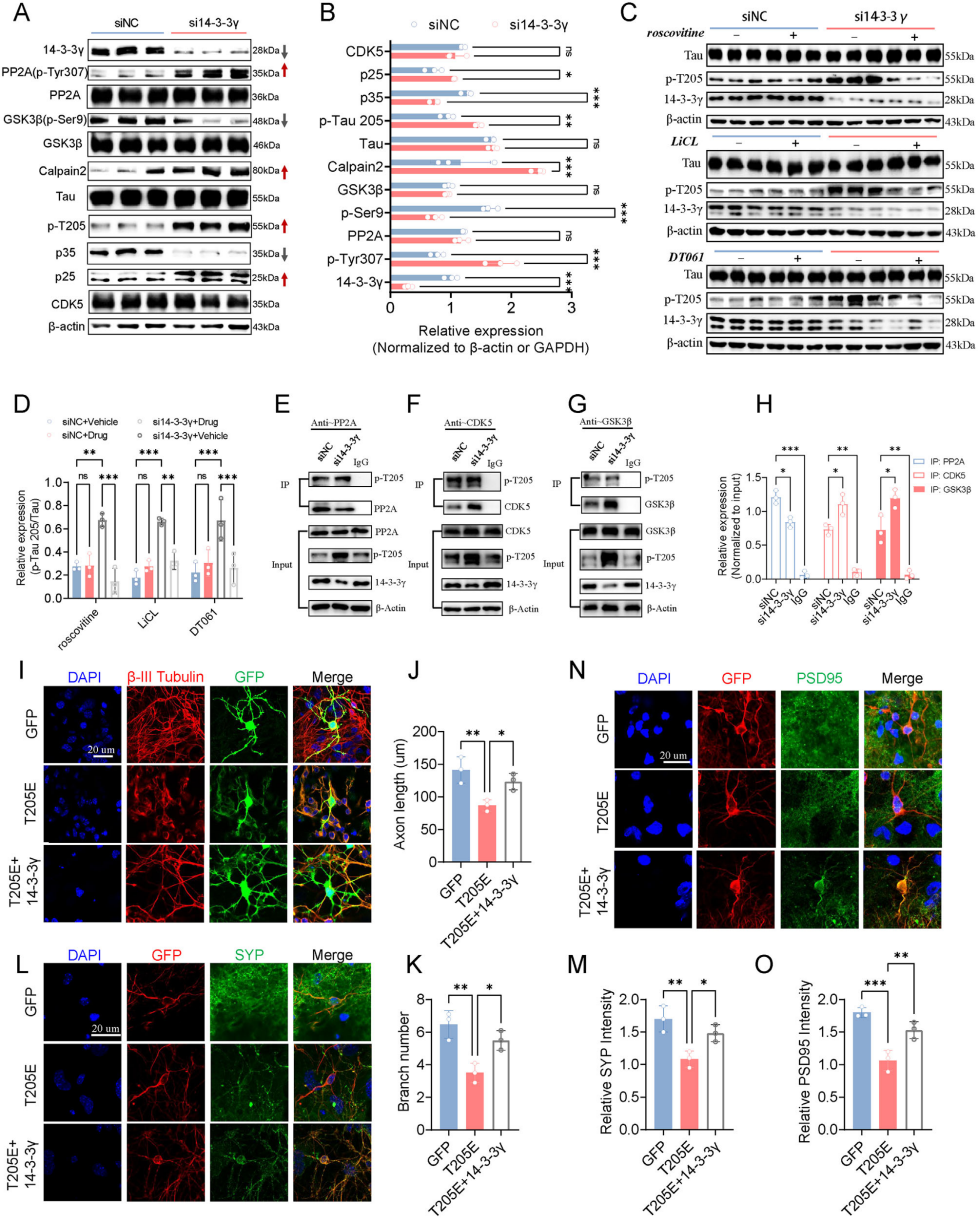
Loss of 14‐3‐3γ is associated with altered kinase–phosphatase signaling and increased Tau Thr205 phosphorylation. (A,B) 14‐3‐3γ knockdown disrupts kinase–phosphatase balance and promotes Tau Thr205 phosphorylation. (A) Representative immunoblot analysis of 14‐3‐3γ, CDK5 pathway components (CDK5, p25, p35), GSK3β signaling (total GSK3β and p‐Ser9), and PP2A activity (total PP2A and p‐Tyr307), along with total Tau and p‐Tau (Thr205), in HT22 hippocampal neuronal cells transfected with control siRNA (siNC) or siRNA targeting 14‐3‐3γ (si14‐3‐3γ). (B) Quantification of protein expression normalized to β‐actin or GAPDH. Knockdown of 14‐3‐3γ leads to activation of CDK5 (increased p25/p35 ratio), activation of GSK3β (reduced inhibitory phosphorylation at Ser9), and inhibition of PP2A (increased p‐Tyr307), accompanied by elevated Tau Thr205 phosphorylation. (C,D) Pharmacological modulation of kinase/phosphatase pathways reverses Tau Thr205 hyperphosphorylation. (C) Representative immunoblot analysis following treatment with CDK5 inhibitor (roscovitine), GSK3β inhibitor (LiCl), or PP2A activator (DT061) in siNC and si14‐3‐3γ cells, showing modulation of Tau Thr205 phosphorylation. (D) Quantification of p‐Tau (Thr205) levels normalized to total Tau, demonstrating that pharmacological inhibition of CDK5 and GSK3β, or activation of PP2A, attenuates si14‐3‐3γ–induced Tau Thr205 hyperphosphorylation. (E,H) 14‐3‐3γ contributes to Tau phosphorylation homeostasis in association with CDK5/GSK3β/PP2A signaling. (E–G) Co‐immunoprecipitation (co‐IP) analyses showing interactions between p‐Tau (Thr205) and PP2A (E), CDK5 (F), and GSK3β (G) in control and 14‐3‐3γ–deficient conditions. (H) Quantification of p‐Tau (Thr205) association with PP2A, CDK5, and GSK3β, indicating decreased association with PP2A and increased association with CDK5 and GSK3β upon 14‐3‐3γ knockdown. (I–O) 14‐3‐3γ overexpression rescues T205E‐induced cytoskeletal and synaptic deficits in primary neurons. (I) Representative immunofluorescence images of primary neurons expressing GFP control, phospho‐mimetic Tau (T205E), or T205E with 14‐3‐3γ overexpression, stained for βIII‐tubulin (red) and GFP (green). (J) Quantification of axonal length, showing reduced neurite extension in T205E‐expressing neurons that is partially restored by 14‐3‐3γ overexpression. (K) Quantification of dendritic branch number across conditions. (L) Representative immunofluorescence images of synaptophysin (SYP) in primary neurons. (M) Quantification of SYP fluorescence intensity. (N) Representative immunofluorescence images of PSD95 in primary neurons. (O) Quantification of PSD95 fluorescence intensity, demonstrating rescue of synaptic protein deficits by 14‐3‐3γ overexpression. Data are presented as mean ± SEM. For cell‐based experiments, n = 3 independent experiments per group. Statistical comparisons were performed using two‐sided unpaired *t*‐tests or one‐way ANOVA with post hoc multiple comparisons where appropriate. Significance levels are indicated as *p < 0.05, **p < 0.01, ***p < 0.001, and ****p < 0.0001; ns, not significant.

We next investigated how 14‐3‐3γ regulates Tau phosphorylation. Knockdown of 14‐3‐3γ in HT22 cells led to activation of CDK5 signaling (increased p25/p35 ratio), activation of GSK3β (reduced inhibitory phosphorylation at Ser9), and inhibition of PP2A (increased inhibitory phosphorylation at Tyr307) (Figure 5A,B). These changes were accompanied by increased Tau Thr205 phosphorylation, consistent with preferential regulation of this phosphorylation site under 14‐3‐3γ deficiency conditions. Pharmacological modulation of these pathways reversed the effects of 14‐3‐3γ deficiency: inhibition of CDK5 (roscovitine), inhibition of GSK3β (LiCl), or activation of PP2A (DT061) significantly reduced Tau Thr205 hyperphosphorylation (Figure 5C,D). Mechanistically, co‐immunoprecipitation analyses showed that loss of 14‐3‐3γ decreased the association of p‐Tau with PP2A while increasing its association with CDK5 and GSK3β (Figure 5E–H), indicating a shift toward kinase‐dominated regulation.

In primary neurons, expression of phospho‐mimetic Tau (T205E) induced neurite degeneration and synaptic deficits, which were significantly rescued by 14‐3‐3γ overexpression (Figure 5I–O). These findings suggest that 14‐3‐3γ contributes to Tau phosphorylation homeostasis in association with CDK5/GSK3β/PP2A signaling.

### 3.6 14‐3‐3γ Preferentially Interacts with Tau in a Thr205 Phosphorylation–Dependent Manner

**FIGURE 6:**
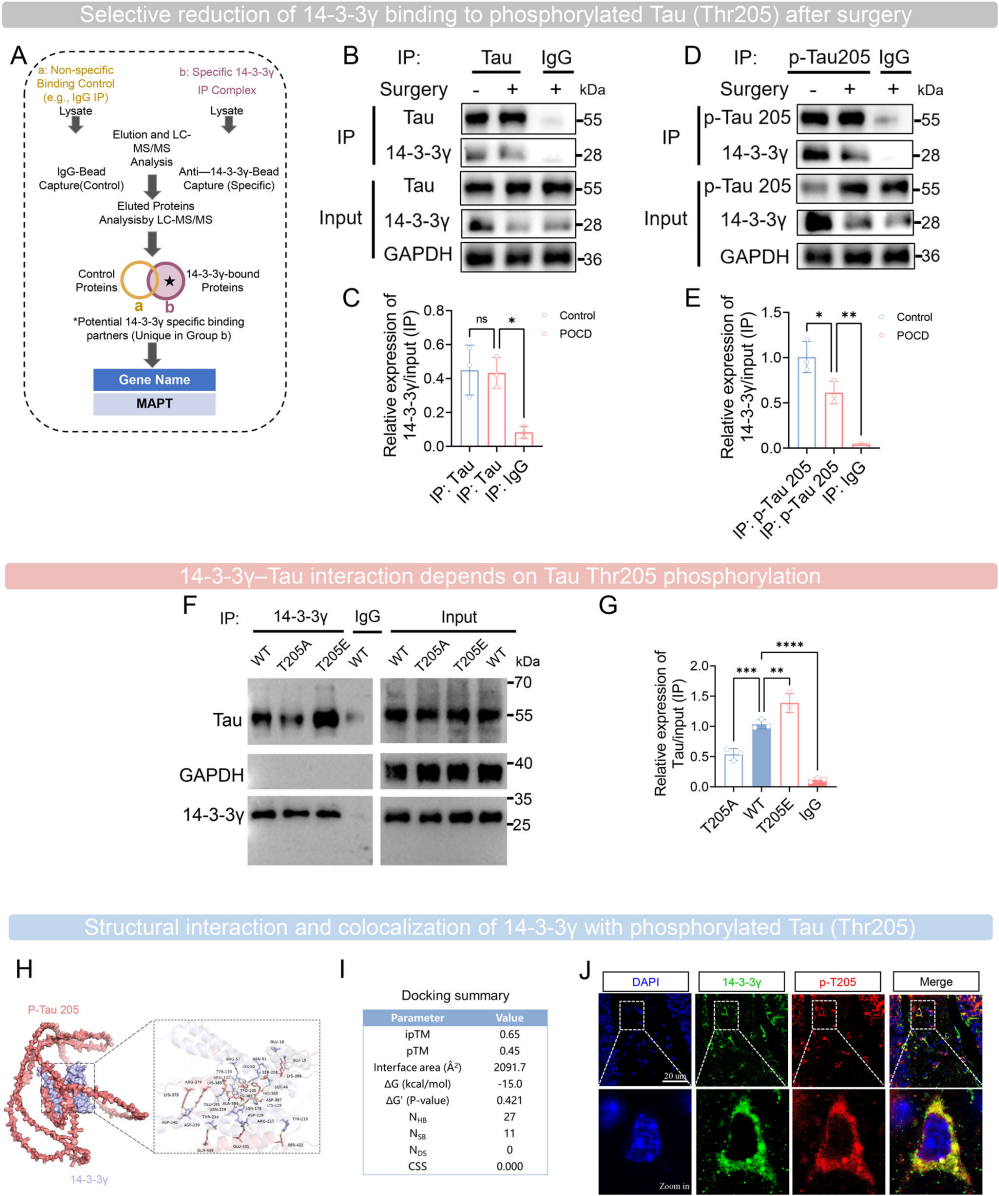
14‐3‐3γ associates with Tau in a Thr205 phosphorylation‐dependent manner. (A–E) Selective reduction of 14‐3‐3y binding to phosphorylated Tau (Thr205) after surgery. (A) Schematic illustration of the immunoprecipitation–mass spectrometry (IP–MS) workflow used to identify 14‐3‐3γ–interacting proteins in hippocampal tissue. Tau (MAPT) was identified as a candidate binding partner of 14‐3‐3γ. (B) Representative co‐immunoprecipitation (co‐IP) of Tau and 14‐3‐3γ from hippocampal lysates of control and surgery mice. (C) Quantification of 14‐3‐3γ binding to Tau (normalized to input), showing reduced interaction after surgery. (D) Co‐IP analysis using p‐Tau (Thr205) antibody, showing association between 14‐3‐3γ and phosphorylated Tau (Thr205) in hippocampal tissue. (E) Quantification of 14‐3‐3γ binding to p‐Tau (Thr205), demonstrating a significant reduction following surgery. (F,G) Phosphorylation‐dependent association. (F) Representative co‐IP showing interaction between 14‐3‐3γ and Tau variants (WT, T205A, T205E). (G), Quantification of Tau binding to 14‐3‐3γ (normalized to input), demonstrating enhanced association with the phospho‐mimetic T205E mutant and reduced binding with the T205A mutant, indicating dependence on Thr205 phosphorylation. (H–J) Structural interaction and colocalization. (H) Structural model of the 14‐3‐3γ–p‐Tau T205 complex obtained from molecular docking. The inset highlights key interface residues mediating hydrogen bonding and hydrophobic interactions between 14‐3‐3γ (blue) and p‐Tau 205 (red). (I) Summary of docking parameters for the 14‐3‐3γ–p‐Tau T205 complex, including interface area (Å^2^), predicted binding free energy (ΔG, kcal/mol), interface residue counts (hydrogen bonds, salt bridges, disulfide interactions), and confidence scores (ipTM, pTM, CSS). (J) Confocal immunofluorescence showing cellular localization of 14‐3‐3γ (green) and p‐Tau T205 (red) in the hippocampal dentate gyrus (DG) region. Nuclei are labeled with DAPI (blue). The merged image shows substantial spatial colocalization of the two signals. Scale bar, 20 µm. Insets show magnified views of selected regions. Data are presented as mean ± SEM. For co‐IP quantification, n = 3 independent experiments per group. Statistical comparisons were performed using two‐sided unpaired t‐tests. Significance levels are indicated as *p < 0.05, **p < 0.01, ***p < 0.001, and ****p < 0.0001; ns, not significant.

To elucidate the molecular mechanism, we performed immunoprecipitation–mass spectrometry, identifying Tau (MAPT) as a candidate binding partner of 14‐3‐3γ (Figure 6A). Co‐immunoprecipitation demonstrated an association between 14‐3‐3γ and Tau, which was significantly reduced following surgery (Figure 6B,C). Importantly, interaction with Thr205‐phosphorylated Tau was also markedly decreased after surgery (Figure 6D,E), suggesting that the association between 14‐3‐3γ and Tau is influenced by Tau phosphorylation status. Using HT22 cells expressing Tau mutants, we demonstrated that 14‐3‐3γ binding is strongly influenced by Thr205 phosphorylation status: the phospho‐mimetic T205E mutant showed enhanced interaction, whereas the phospho‐deficient T205A mutant exhibited reduced binding (Figure 6F,G). Structural modeling further supported a putative interaction interface between 14‐3‐3γ and phosphorylated Tau at Thr205 (Figure 6H,I). Immunofluorescence analysis revealed strong colocalization between 14‐3‐3γ and p‐Tau (Thr205) signals within the hippocampal dentate gyrus (DG) region (Figure 6J). Together, these data support an association between 14‐3‐3γ and Tau that is influenced by Tau phosphorylation status.

### 3.7 Pharmacological Enhancement of 14‐3‐3γ–Tau Interaction Ameliorates POCD

**FIGURE 7:**
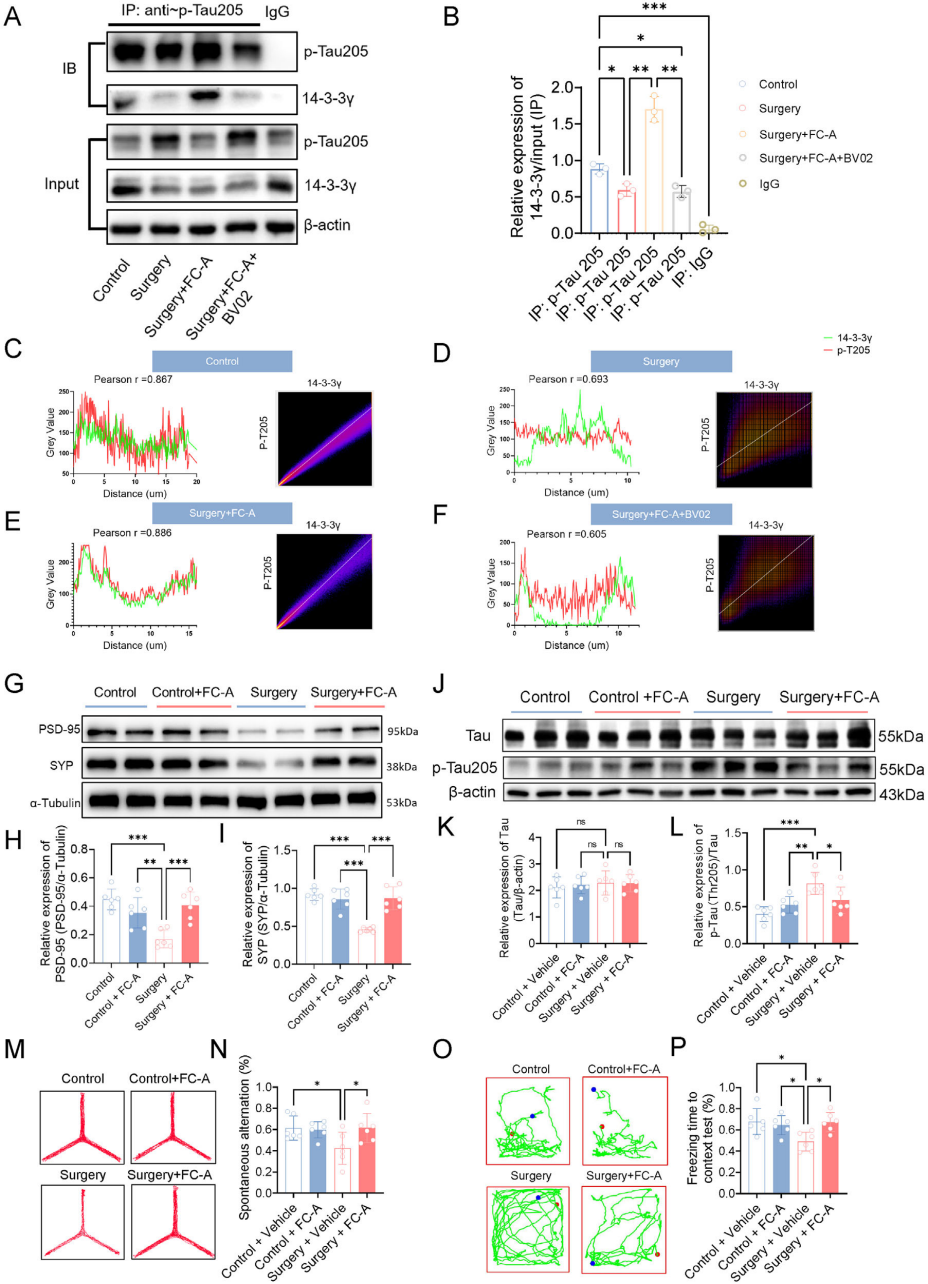
Small molecule FC‐A enhances 14‐3‐3γ–p‐Tau (Thr205) interaction and alleviates postoperative cognitive impairment. (A,B) FC‐A enhances 14‐3‐3γ binding to phosphorylated Tau (Thr205). (A) Representative co‐immunoprecipitation (co‐IP) of 14‐3‐3γ with p‐Tau (Thr205) in hippocampal lysates from control, surgery, surgery + FC‐A, and surgery + FC‐A + BV02 groups. (B) Quantification of 14‐3‐3γ binding to p‐Tau (Thr205) normalized to input, demonstrating that FC‐A significantly restores the interaction disrupted by surgery, while the 14‐3‐3 inhibitor BV02 attenuates this effect (n = 3 independent experiments; *p < 0.05, **p < 0.01, ***p < 0.001). (C–F) Confocal immunofluorescence showing colocalization of 14‐3‐3γ (green) and p‐Tau (Thr205, red) in hippocampal neurons. Line‐scan intensity profiles and Pearson's correlation coefficients indicate reduced colocalization after surgery (D) and recovery following FC‐A treatment (E), which is partially reversed by BV02 (F). (G–I) Representative immunoblots (G) and quantification of PSD‐95 (H) and synaptophysin (SYP, I) in hippocampal tissue. Surgery reduced synaptic protein levels, which were significantly rescued by FC‐A treatment (n = 6; mean ± SEM; ***p < 0.001). (J–L) Representative immunoblots (J) and quantification of total Tau (K) and p‐Tau Thr205 (L). FC‐A reduces surgery‐induced Tau T205 hyperphosphorylation without altering total Tau levels (n = 6; mean ± SEM; *p < 0.05, **p < 0.01, ***p < 0.001). (M–P) Behavioral analyses. (M) Representative Y‐maze traces. (N) Quantification of spontaneous alternation in the Y‐maze, showing improved working memory with FC‐A. (O) Representative open field trajectories. (P) Quantification of contextual freezing time in fear conditioning, demonstrating enhanced hippocampus‐dependent memory after FC‐A administration (n = 6; mean ± SEM; *p < 0.05, **p < 0.01).

To determine whether pharmacological targeting of the 14‐3‐3γ–Tau interaction could confer therapeutic benefit, surgery mice were treated with the small molecule FC‐A. Co‐immunoprecipitation assays showed that FC‐A markedly enhanced the binding between 14‐3‐3γ and phosphorylated Tau (Thr205), reversing the surgery‐induced reduction (Figure 7A–B). The 14‐3‐3 inhibitor BV02 partially blocked this effect, supporting the involvement of 14‐3‐3γ in the FC‐A‐enhanced association between 14‐3‐3γ and phosphorylated Tau. Immunofluorescence analyses demonstrated that surgery decreased colocalization of 14‐3‐3γ and p‐Tau 205 in hippocampal neurons. FC‐A restored their colocalization, which was partially reversed by BV02 (Figure 7C–F). At the biochemical level, surgery induced a significant reduction of synaptic proteins PSD‐95 and SYP; FC‐A treatment effectively rescued these synaptic deficits (Figure 7G–I). Concurrently, FC‐A significantly reduced Tau Thr205 hyperphosphorylation without affecting total Tau levels (Figure 7J–L), indicating modulation of this disease‐associated phosphorylation event.

**FIGURE 8:**
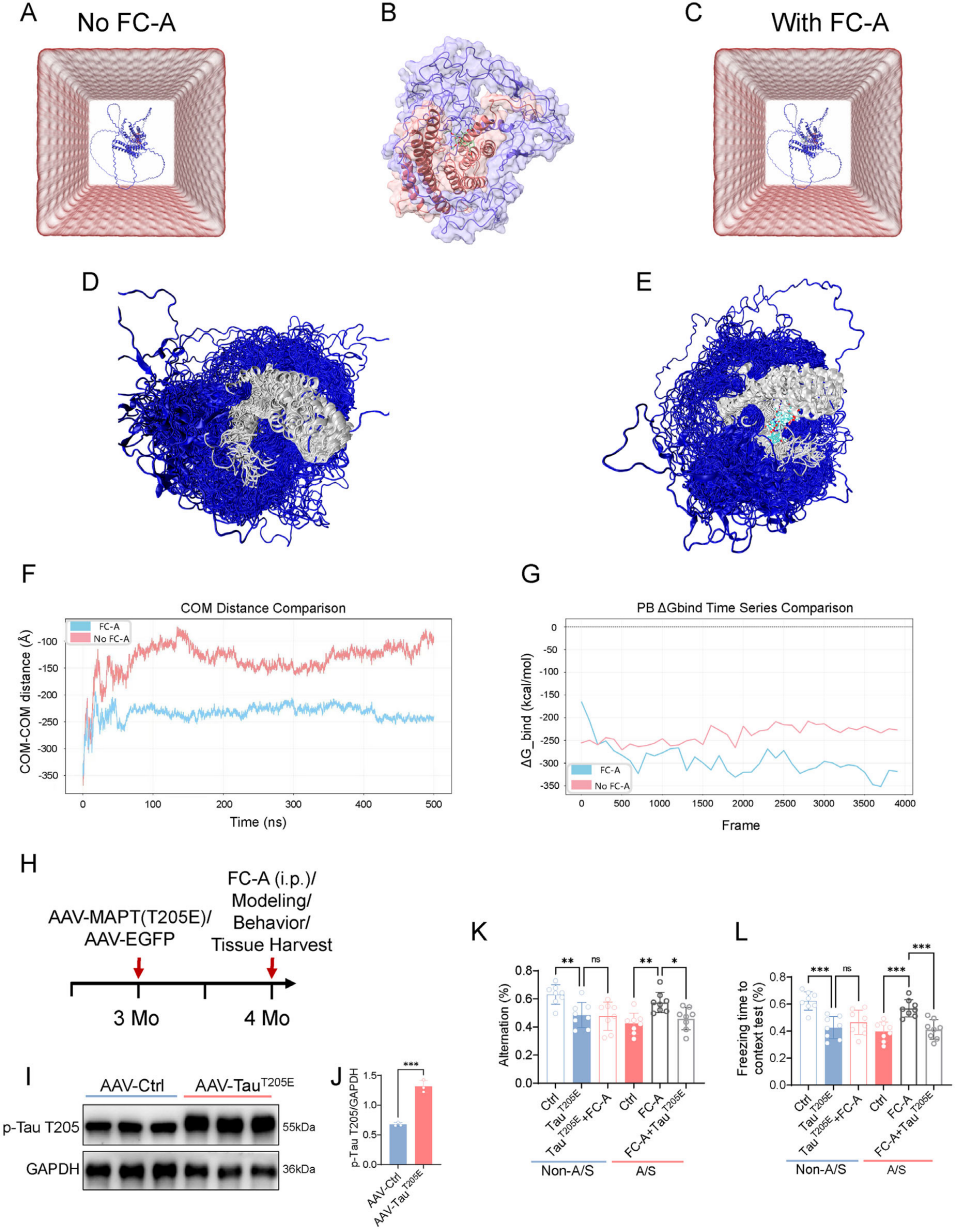
FC ‐ A stabilizes the 14 ‐ 3 ‐ 3γ–p ‐ Tau Thr205 interaction and its cognitive protection is dependent on Tau Thr205 phosphorylation in mice. (A–C) Representative conformational ensembles of the 14‐3‐3γ–p‐Tau T205 complex in the absence (A) or presence (C) of FC‐A over 0–500 ns molecular dynamics simulations, illustrating differences in structural compactness. (B) Structural model of the 14‐3‐3γ–p‐Tau T205 complex highlighting the binding interface. (D,E) Superimposed trajectories showing the conformational space sampled by the complex without (D) or with (E) FC‐A, indicating reduced structural fluctuation and increased compactness in the presence of FC‐A. (F) Time evolution of the center‐of‐mass (COM) distance between 14‐3‐3γ and p‐Tau T205 during simulations. The FC‐A–bound system exhibits consistently shorter COM distances, indicating enhanced interaction stability. (G) Binding free energy (ΔG_bind) calculated using the Poisson–Boltzmann method across simulation frames. The FC‐A–bound complex shows a more negative ΔG_bind, consistent with increased binding affinity. (H–L) In vivo validation of Thr205 dependency. (H) Experimental timeline. (I‐J) Western blot confirmation of TauT205E expression. (K,L) Behavioral assessments (Y‐maze and fear conditioning) showing that TauT205E expression abolishes FC‐A‐mediated cognitive protection (n = 8 per group; one‐way ANOVA with Tukey's test; *p < 0.05, **p < 0.01, ***p < 0.001).

Behaviorally, FC‐A improved hippocampus‐dependent cognitive function. Surgery mice exhibited impaired working memory and contextual fear memory, as evidenced by reduced spontaneous alternation in the Y‐maze and decreased freezing in fear conditioning. FC‐A treatment restored these behaviors toward control levels (Figure 7M–P). Finally, to probe the molecular mechanism, molecular dynamics simulations of the 14‐3‐3γ–p‐Tau T205 complex with or without FC‐A were performed (Figure 8A–G). FC‐A binding reduced the center‐of‐mass distance and structural fluctuation (Figure 8F), while increasing the binding free energy magnitude (ΔG_bind) (Figure 8G), consistent with enhanced interaction stability. These results support the notion that FC‐A stabilizes the 14‐3‐3γ–Tau interface, providing a molecular basis for the observed biochemical and functional rescue. Collectively, these findings demonstrate that pharmacological stabilization of the 14‐3‐3γ–p‐Tau interaction can restore synaptic protein expression and hippocampus‐dependent cognition following surgery, highlighting FC‐A as a promising therapeutic strategy for postoperative cognitive dysfunction.

### 3.8 Tau Thr205 Phosphomimetic Expression Compromises FC‐A‐Mediated Cognitive Protection

To determine whether modulation of Tau Thr205 phosphorylation contributes to the cognitive benefits conferred by 14‐3‐3γ stabilization, a phosphomimetic Tau mutant (TauT205E) was expressed in the hippocampus using AAV‐mediated gene delivery (Figure 8H). Western blot analysis confirmed successful expression of TauT205E, resulting in markedly elevated p‐Tau Thr205 levels compared with AAV‐control mice (Figure 8I,J). In the absence of anesthesia and surgery, TauT205E expression alone was sufficient to impair cognitive performance. Compared with control mice, TauT205E‐expressing animals exhibited significantly reduced spontaneous alternation in the Y‐maze and decreased freezing behavior during contextual fear testing, indicating deficits in both working memory and hippocampus‐dependent memory (Figure 8K,L). Importantly, FC‐A treatment failed to improve these impairments, suggesting that persistent Tau Thr205 phosphomimetic signaling can overcome the protective effects mediated by 14‐3‐3γ stabilization. Consistent with previous experiments, FC‐A significantly improved behavioral performance following anesthesia and surgery. However, hippocampal expression of TauT205E largely abolished these protective effects. Despite FC‐A administration, mice expressing TauT205E displayed reduced spontaneous alternation and contextual freezing responses compared with FC‐A‐treated animals, indicating persistent cognitive dysfunction (Figure 8K,L).

Together, these findings demonstrate that enforced Tau Thr205 phosphomimetic signaling is sufficient to induce cognitive impairment and renders animals unresponsive to FC‐A‐mediated neuroprotection. These results support a model in which Tau Thr205 phosphorylation represents an important downstream component of 14‐3‐3γ‐mediated neuroprotection, and modulation of this phosphorylation event contributes to the cognitive benefits of FC‐A.

## 4 Discussion

In this study, we demonstrate that 14‐3‐3γ serves as a consistent biomarker and mechanistic regulator of cognitive dysfunction in acute perioperative contexts. Our multi‐cohort CSF proteomics revealed that 14‐3‐3γ is elevated in individuals exhibiting neurodegeneration‐enriched cognitive vulnerability (NECV), a phenotype characterized by prior surgical exposure and objective cognitive impairment. These observations were recapitulated in perioperative plasma, where higher 14‐3‐3γ levels were associated with subsequent POD and POCD, providing preliminary translational evidence for its potential relevance to perioperative neurocognitive outcomes. Mechanistically, hippocampal 14‐3‐3γ depletion in murine surgery models drives selective Tau Thr205 hyperphosphorylation, microtubule fragmentation, and synaptic loss, which are rescued by neuron‐specific 14‐3‐3γ overexpression or pharmacological stabilization of the 14‐3‐3γ–Tau interaction.

Previous studies have established associations between 14‐3‐3 proteins and cognitive function in the context of Alzheimer's disease (AD). Specifically, 14‐3‐3 proteins interact with tau and modulate its phosphorylation, aggregation, and toxicity [41]. Elevated levels of 14‐3‐3 proteins in cerebrospinal fluid have been suggested as a biomarker for AD [21]. More recently, multiplex proteomic analysis of the ADNI cohort identified YWHAG (encoding 14‐3‐3γ) as one of the most consistent biomarkers for both diagnosis and prediction of cognitive decline, with strong associations with tau pathology, amyloid burden, and cognitive performance [21]. However, the role of 14‐3‐3γ in the acute perioperative setting—specifically, its response to surgical stress and its mechanistic contribution to postoperative cognitive dysfunction—has remained unexplored. Several critical distinctions make our investigation novel. First, while previous studies have focused on chronic neurodegenerative conditions, we demonstrate that 14‐3‐3γ levels change dynamically within days after surgical insult, with opposing directionality between central (brain tissue) and peripheral (plasma/CSF) compartments. Second, we establish a causal link between 14‐3‐3γ depletion and site‐specific tau phosphorylation at Thr205, a post‐translational modification not previously investigated in the context of POCD. Third, we provide preclinical proof‐of‐concept that pharmacological stabilization of the 14‐3‐3γ‐tau interaction using FC‐A can ameliorate surgery‐induced cognitive deficits in experimental model. These findings extend the known biology of 14‐3‐3γ from a passive biomarker of neurodegeneration to an active, druggable regulator of acute cognitive vulnerability.

A particularly intriguing observation is the compartment‐specific divergence in 14‐3‐3γ expression: hippocampal tissue showed marked downregulation after surgery, while plasma and CSF levels were elevated. While direct evidence for compartment‑specific divergence of 14‑3‑3γ expression after surgical stress remains limited, increased levels of 14‑3‑3 proteins in cerebrospinal fluid and other extracellular fluids have been reported in various neurodegenerative and rapidly progressive neurological disorders. Detection of CSF 14‑3‑3γ has been used as a diagnostic marker in prion disease and is correlated with other markers of neuronal damage such as tau and phosphorylated tau [23, 42]. Moreover, CSF 14‑3‑3 proteins are frequently elevated in non‑prion neurodegenerative conditions, reflecting neuronal injury and potential leakage of intracellular proteins into extracellular fluids [43]. This apparent paradox warrants careful consideration. Several mechanisms may explain this phenomenon. First, increased levels of 14‐3‐3 proteins in CSF and other extracellular fluids have been documented in various neurological conditions characterized by neuronal injury, including prion disease, stroke, and traumatic brain injury [41]. In these contexts, elevated extracellular 14‐3‐3 proteins are thought to reflect leakage from damaged neurons rather than active secretion. Detection of CSF 14‐3‐3γ has been used as a diagnostic adjunct for Creutzfeldt‐Jakob disease and correlates with other neuronal injury markers such as tau and phosphorylated tau [23]. Second, the observed elevation in plasma 14‐3‐3γ in POCD patients may similarly represent a ‘spillover’ phenomenon from injured central nervous system compartments. Consistent with this interpretation, a previous proteomic study in aged rats with delayed neurocognitive recovery identified decreased 14‐3‐3β/α expression in the hippocampus, prefrontal cortex, and temporal lobe, while serum levels were also reduced in geriatric postoperative delirium patients [44], suggesting that isoform‐specific patterns of central‐peripheral correlation may differ. Third, the intracellular depletion of 14‐3‐3γ in hippocampal neurons following surgical stress—rather than the extracellular elevation—likely represents the pathologically relevant event for synaptic vulnerability. The 14‐3‐3 family proteins are constitutively abundant in the brain, comprising approximately 1% of total soluble protein. Their depletion would disrupt multiple protein‐protein interaction networks, including those regulating tau phosphorylation, mitochondrial function, and synaptic maintenance. We propose that while extracellular 14‐3‐3γ elevation serves as a clinically useful peripheral biomarker of neuroaxonal injury, the intracellular loss in vulnerable brain regions (e.g., hippocampus) may contribute to the molecular cascade leading to Tau hyperphosphorylation and cognitive impairment.

Another mechanistic insight is the site‐specific Tau phosphorylation observed at Thr205 in hippocampal neurons seven days post‐surgery. While we did not observe significant changes at Ser202, Thr231, Ser396, or Ser262 at this timepoint, it is plausible that phosphorylation at these or other residues occurs transiently at earlier stages (postoperative days 1–3) or in different subcellular compartments. Tau phosphorylation is a highly dynamic process regulated by multiple kinases and phosphatases, and selective accumulation at Thr205 may reflect a delayed or persistent pathological mark at seven days post‐insult, which corresponds with observed synaptic and cognitive deficits [45, 46, 47]. Future studies with temporally resolved analyses could clarify the kinetics of site‐specific Tau phosphorylation and the interplay between transient and persistent modifications. These insights align with prior reports showing that selective phosphorylation sites have distinct impacts on microtubule stability and neuronal vulnerability. Although our findings highlight Tau Thr205 phosphorylation as an important molecular event associated with surgery‐induced cognitive impairment, Tau contains numerous phosphorylation sites that may have distinct or synergistic effects on Tau function. In the present study, we examined several representative Tau phosphorylation sites based on previous evidence and our preliminary observations; however, we cannot exclude the involvement of additional phosphorylation events. Future unbiased phosphoproteomic analyses will be required to comprehensively characterize surgery‐induced Tau phosphorylation changes and determine their relative contributions to cognitive vulnerability.

A third critical consideration is the isoform specificity of 14‐3‐3. The 14‐3‐3 family comprises multiple isoforms (β, ε, γ, ζ, η, σ, τ), each with overlapping but non‐identical client proteins and functional roles [48]. We focused on 14‐3‐3γ because of its prominent signal in CSF proteomics, its consistent associations with cognitive and neurodegenerative measures, and prior evidence linking γ to Tau pathology and synaptic regulation [21]. Nevertheless, other isoforms may contribute to both acute and chronic cognitive dysfunction. Preliminary Western blot analyses in our lab suggest differential regulation of other isoforms post‐surgery, warranting comprehensive investigation. Understanding isoform‐specific contributions is critical for mechanistic clarity and for guiding targeted therapeutic strategies, especially when considering small‐molecule 14‐3‐3 modulators with potential isoform selectivity.

Compared with prior studies, our work uniquely bridges human biomarker discovery, peripheral translation, and mechanistic validation in vivo. While previous studies have highlighted 14‐3‐3 isoforms in AD [21], none have integrated multi‐cohort CSF proteomics, plasma validation, and causally validated neuronal mechanisms linking 14‐3‐3γ to site‐specific Tau phosphorylation, synaptic integrity, and cognitive outcomes. Moreover, our pharmacological intervention demonstrates proof‐of‐concept for therapeutic modulation of the 14‐3‐3γ–Tau axis, offering a strategy distinct from conventional anti‐amyloid or anti‐tau therapies.

Several limitations should be acknowledged. First, the temporal resolution of Tau phosphorylation is limited to postoperative day 7 in hippocampal tissue, which may overlook early transient changes at other phosphorylation sites or in other brain regions. Second, while we focused on 14‐3‐3γ because of its prominent signal in proteomic analyses and its consistent associations with cognitive and neurodegenerative phenotypes, other isoforms may modulate cognitive outcomes; comprehensive profiling of all isoforms is warranted. Third, species differences in 14‐3‐3γ regulation and Tau dynamics may affect translational relevance of murine findings to humans. Fourth, our pharmacological studies employed a single small‐molecule modulator (FC‐A); dose‐response relationships, chronic administration effects, and safety profiles remain to be fully evaluated. Fifth, the ADNI cohort was not specifically designed to investigate perioperative neurocognitive disorders and lacks detailed information regarding surgical timing, anesthesia exposure, preoperative cognitive status, and postoperative cognitive trajectories. Therefore, the ADNI‐based discovery analysis should be interpreted as hypothesis‐generating rather than direct evidence of POCD‐associated biomarkers. Sixth, although early postoperative plasma 14‐3‐3γ was associated with subsequent POD or POCD, the relatively small number of outcome events limited the development of robust multivariable prediction models. Seventh, the predictive models should be considered exploratory and hypothesis‐generating rather than clinically deployable tools. Future studies involving larger, multicenter cohorts with standardized perioperative cognitive assessments and biomarker measurements are required to evaluate their generalizability and clinical utility. Eighth, our experiments were performed in young adult mice rather than aged animals. Given that advanced age is a major risk factor for perioperative neurocognitive disorders, future studies using aged animal models are warranted to further validate the translational relevance of the 14‐3‐3γ–Tau pathway. Ninth, the experimental studies included only male mice, and potential sex‐dependent differences in neuroinflammation, synaptic plasticity, and Tau‐related signaling cannot be excluded. Tenth, although our findings suggest that 14‐3‐3γ may influence Tau phosphorylation through modulation of CDK5/GSK3β/PP2A signaling homeostasis, the current study does not establish whether these effects are mediated by direct regulation of kinase or phosphatase activity or represent secondary consequences of altered Tau phosphorylation. Further studies using biochemical approaches, such as direct interaction assays and kinase/phosphatase activity measurements, are required to elucidate the precise molecular mechanism. Finally, although we controlled for major clinical confounders in perioperative cohorts, unmeasured variables such as systemic inflammation, comorbidities, or genetic predisposition may influence peripheral 14‐3‐3γ levels and cognitive outcomes.

## 5 Conclusions

These findings identify 14‐3‐3γ as a potential molecular regulator of postoperative cognitive vulnerability and suggest the 14‐3‐3γ–Tau Thr205 signaling pathway as a candidate therapeutic target for POCD.

## Author Contributions

Conceptualization: **Xiaoping Gu**, **Shouqiang Zhu**; Methodology: **Shouqiang Zhu**, **Tianjiao Xia**, **Chang Ye**; Software: **Shouqiang Zhu**, **Xue Han**, **Heng Zhang**; Formal analysis: **Shouqiang Zhu**; Data curation: **Shouqiang Zhu**, **Tianjiao Xia**, **Chang Ye**; Writing – original draft: **Shouqiang Zhu**; Writing – review & editing: **Xiaoping Gu**, **Tianjiao Xia**, **Chang Ye**; Supervision: **Xiaoping Gu**, **Tianjiao Xia**, **Chang Ye**.

## Funding

This work was supported by the Jiangsu Provincial Medical Key Discipline (Grant No. ZDXK202232 to X.P.G.). The funder had no role in study design, data collection and analysis, decision to publish, or preparation of the manuscript.

## Ethics Approvals and Clinical Study Registration

The retrospective analyses of publicly available cohort data were conducted in accordance with the ethical requirements of the original studies. The Alzheimer's Disease Neuroimaging Initiative (ADNI) and the Parkinson's Progression Markers Initiative (PPMI) are multicentre longitudinal observational studies with protocols approved by the institutional review boards at each participating site. Participants (or their authorized representatives) provided written informed consent for data collection, storage, and sharing at the time of enrollment, and all data were de‑identified prior to release for research use. Investigators accessed ADNI and PPMI data under the respective Data Use Agreements and Publication Policies, with no new human subjects recruitment required for these secondary analyses. The prospective clinical cohort of surgical patients was approved by the Ethics Committee of Nanjing Drum Tower Hospital, affiliated with Nanjing University Medical School (Approval No. 2024‑902‑02), and registered at the Chinese Clinical Trial Registry (ChiCTR2500096293). Written informed consent was obtained from all participants or their legal representatives prior to enrollment, and all procedures were performed in accordance with the Declaration of Helsinki and local regulations. The Animal Ethics Committee of Nanjing Drum Tower Hospital approved the animal feeding and experimental operation in this study. The approval time for animal ethics was April 7th, 2025, and the number was 2025AE01169.

## Conflicts of Interest

The authors declare no conflicts of interest.

## Supporting information




**Supporting File 1**: advs77480‐sup‐0001‐SuppMat.pdf.

**Supporting File 2**: advs77480‐sup‐0002‐SuppMat.pdf.

## Data Availability

The ADNI and PPMI datasets used in this study are publicly available through controlled‐access repositories. Researchers can obtain access by submitting an application through the ADNI Data Archive (http://adni.loni.usc.edu/) and PPMI data portal (https://www.ppmi‐info.org/), subject to approval of the respective data‐use agreements. Data from the prospective cohort at the Affiliated Drum Tower Hospital of Nanjing University Medical School contains sensitive patient‐level clinical information and cannot be publicly shared due to ethical and legal restrictions. Researchers who wish to request access to de‐identified data may submit a request to the Data Access Committee of Nanjing Drum Tower Hospital through the hospital's official website: https://www.njglyy.com/ or via the institutional contact email: gyethics@163.com. All requests will be reviewed in accordance with institutional policies and applicable data protection regulations. The authors do not have special access privileges to these data. All molecular biology experimental data are available from the corresponding author (XP.G., xiaopinggu@nju.edu.cn) upon reasonable request.
