# Supplementary material for: 14‐3‐3γ Protects Against Postoperative Cognitive Dysfunction by Regulating Tau Thr205 Phosphorylation and Synaptic Integrity
Source: Adv Sci (Weinh). 2026 Sep 27:e77480. Online ahead of print. doi: 10.1002/advs.77480 (PMC13616388; doi:10.1002/advs.77480)

Fig. 3J

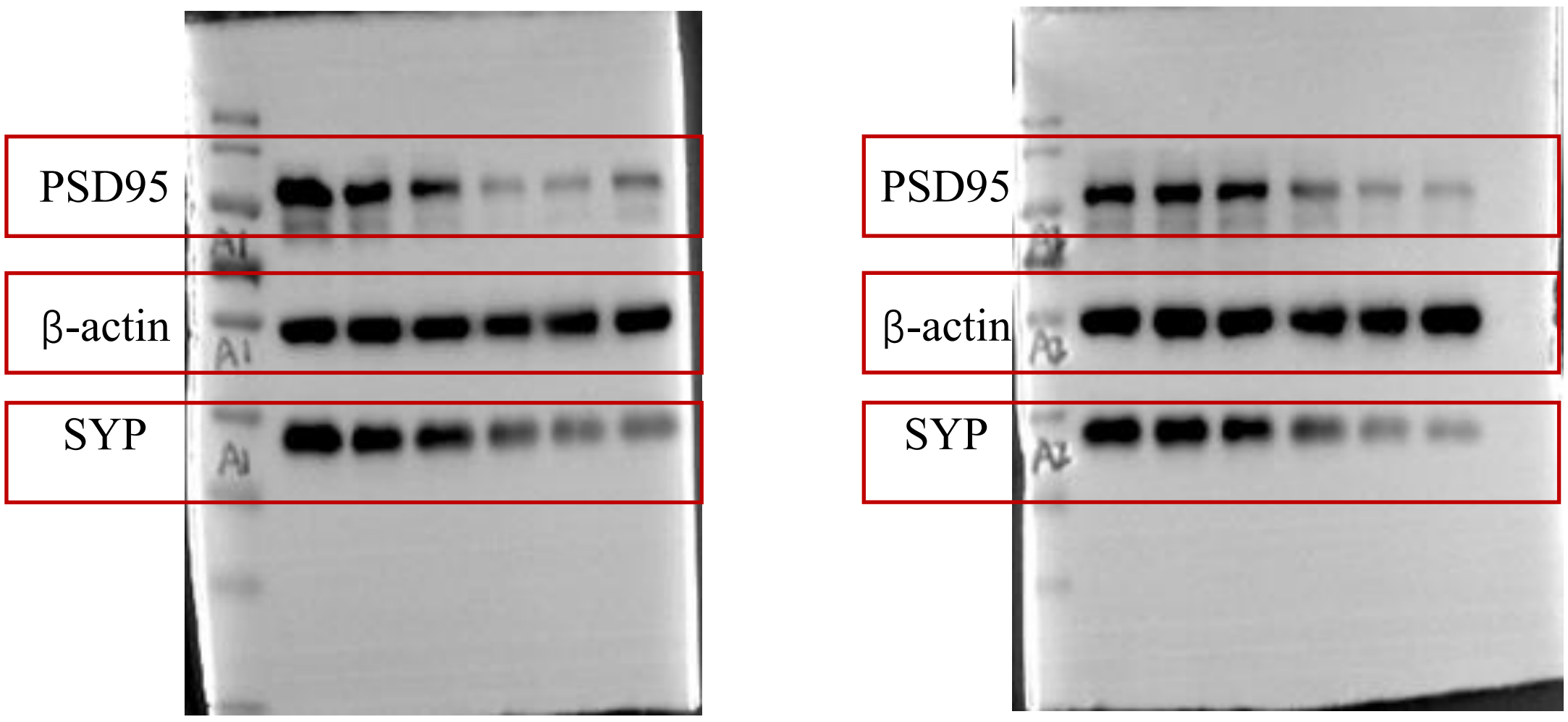

Fig. 3Q-Day 1

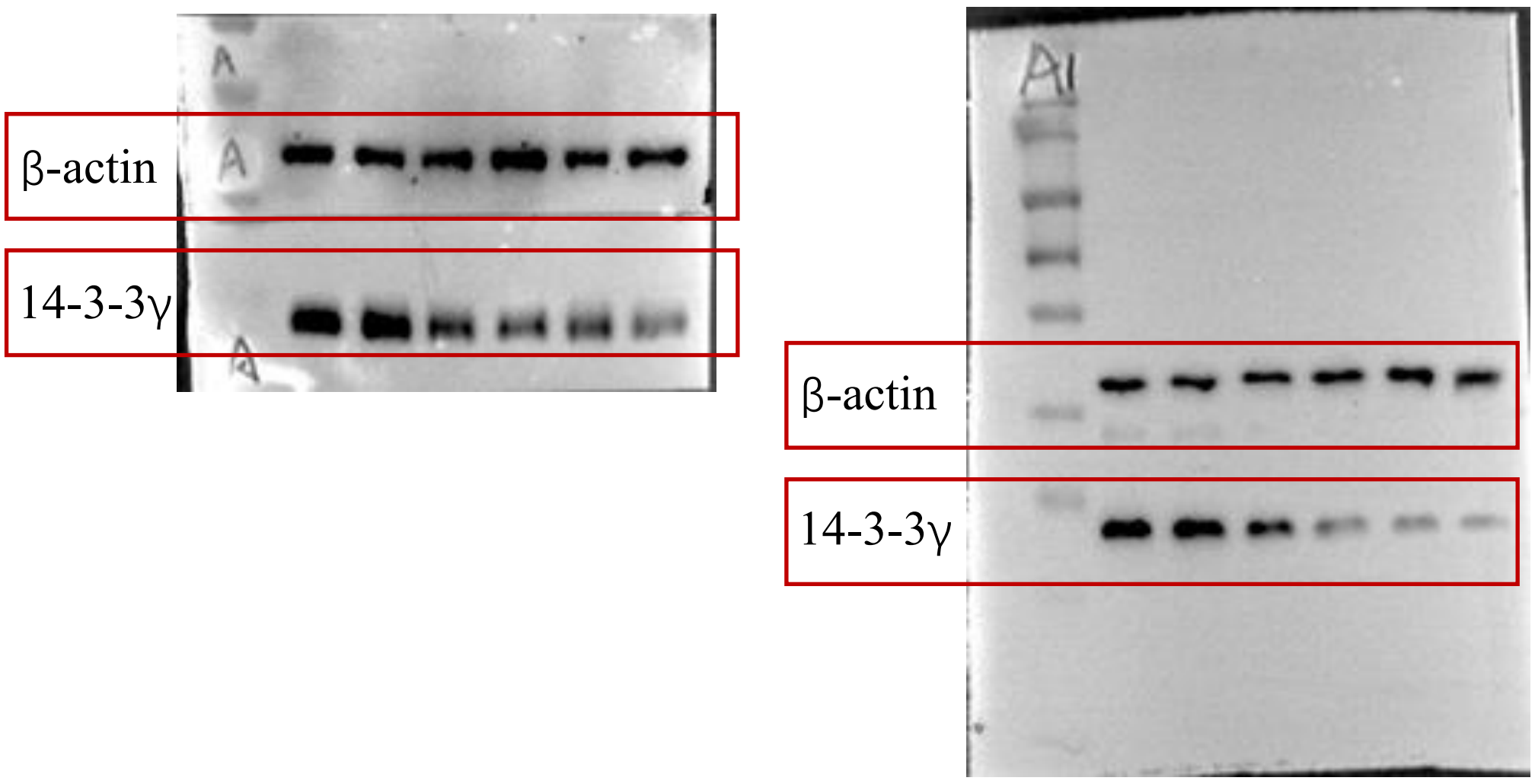

Fig. 3Q-Day 2

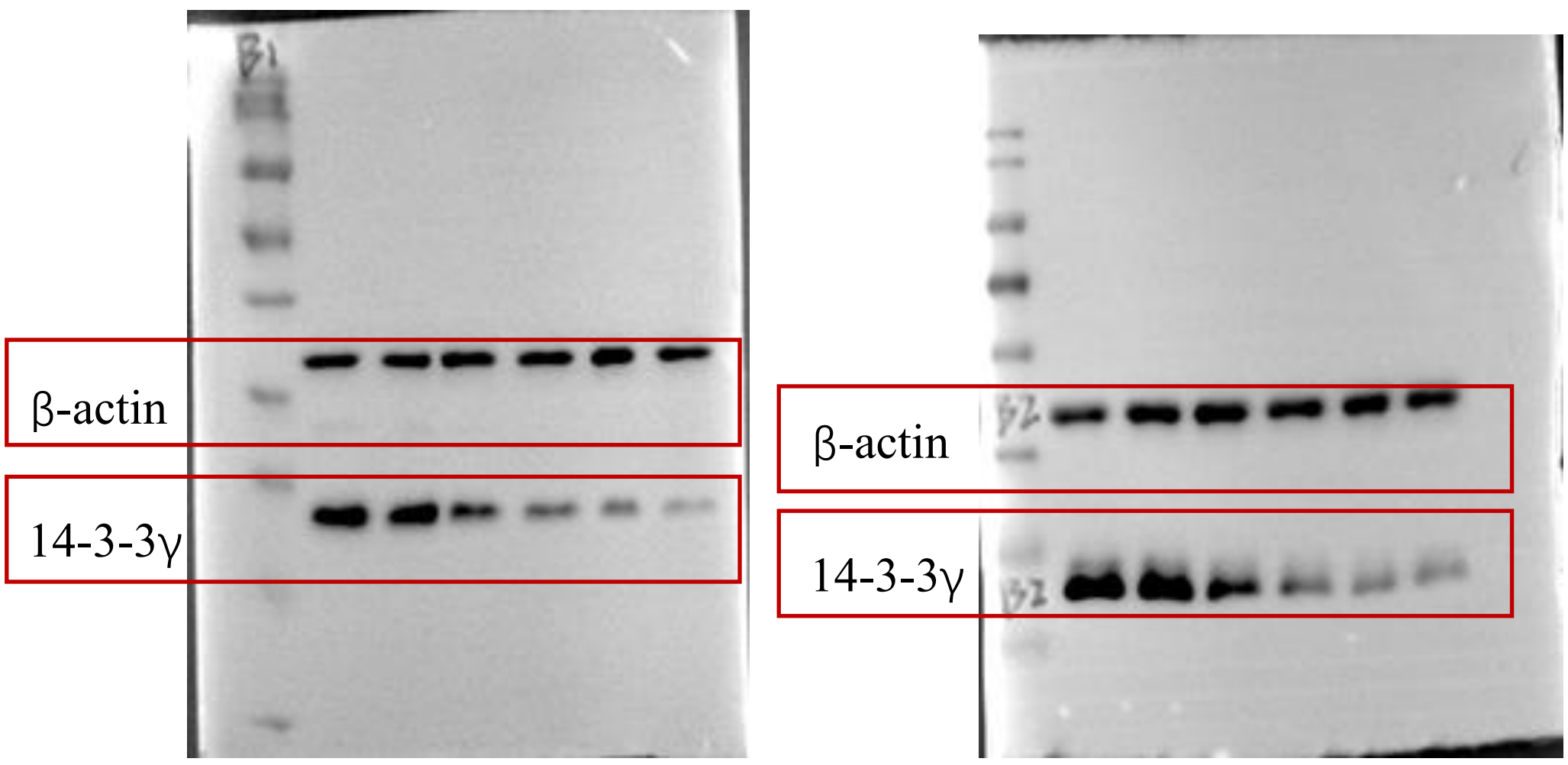

Fig. 3Q-Day 3

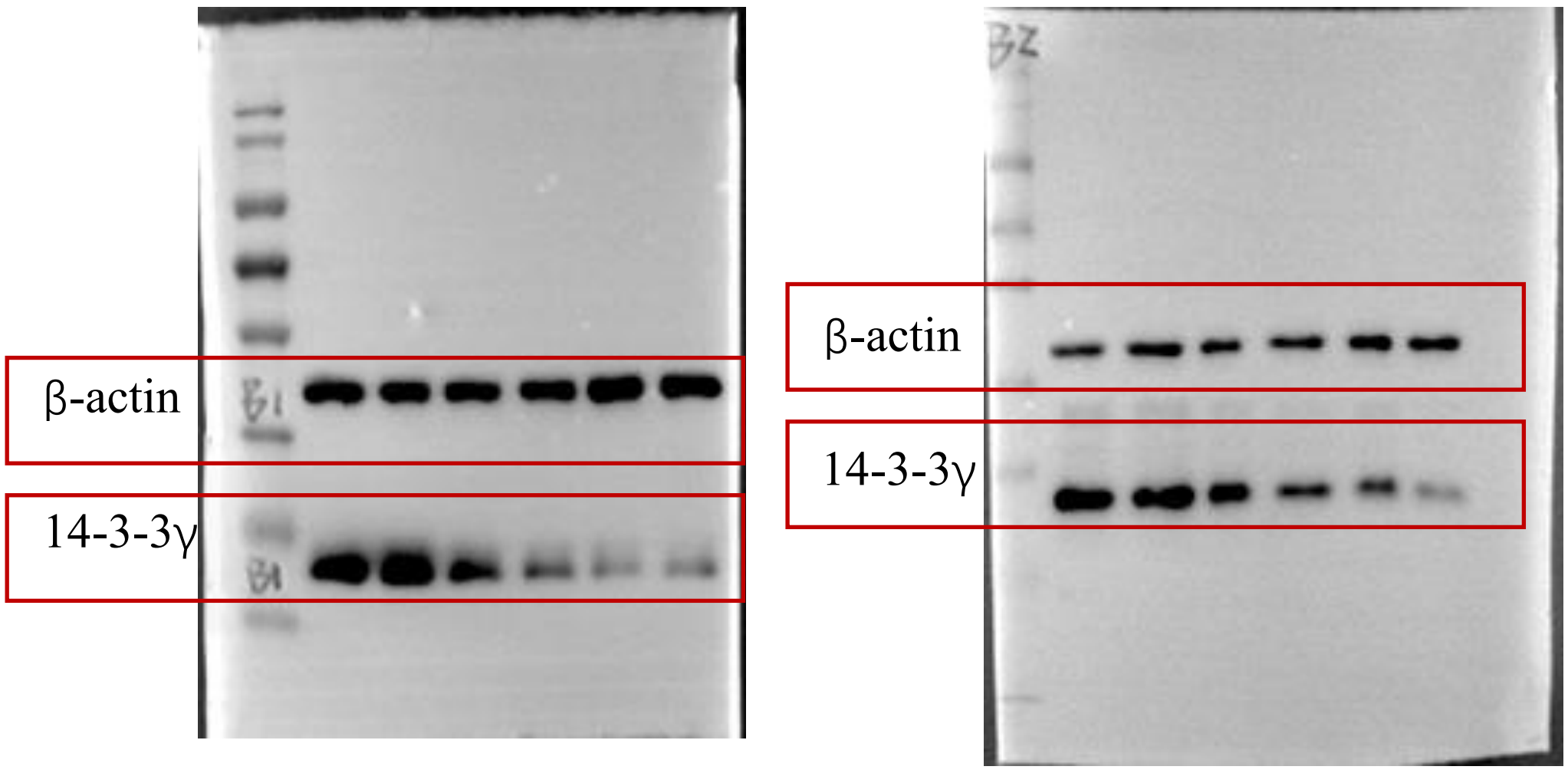

Fig. 3S-AT8

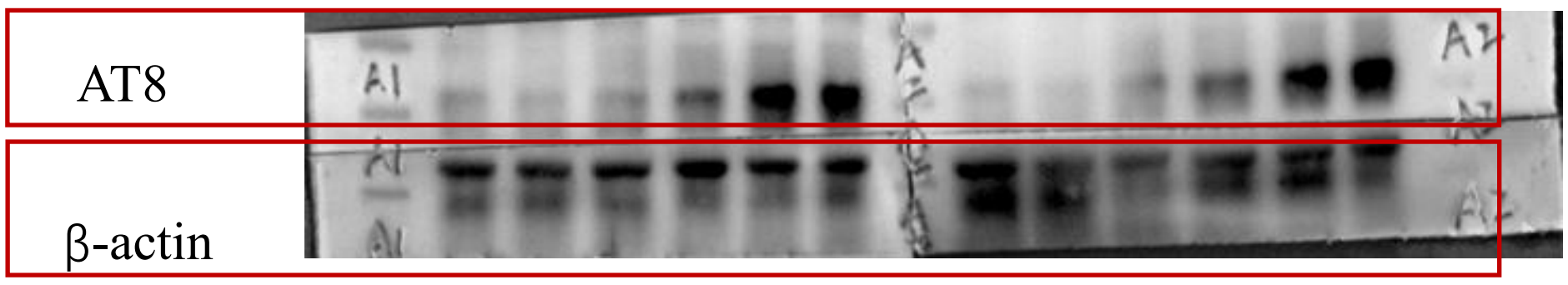

Fig. 3S-S262

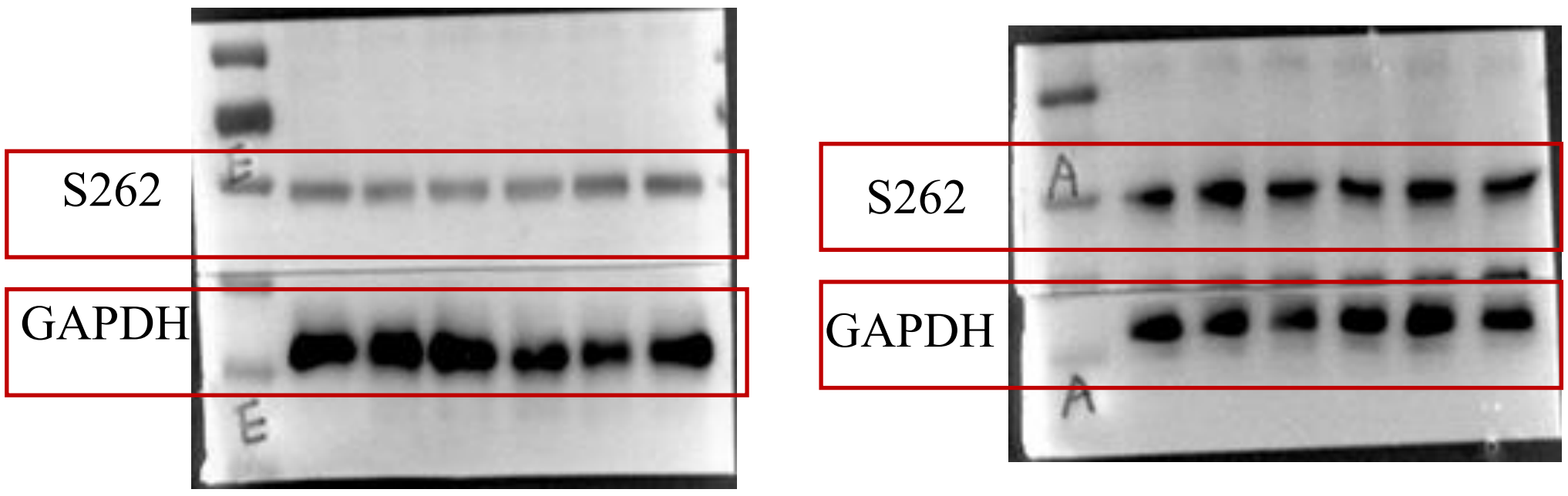

Fig. 3S-S396

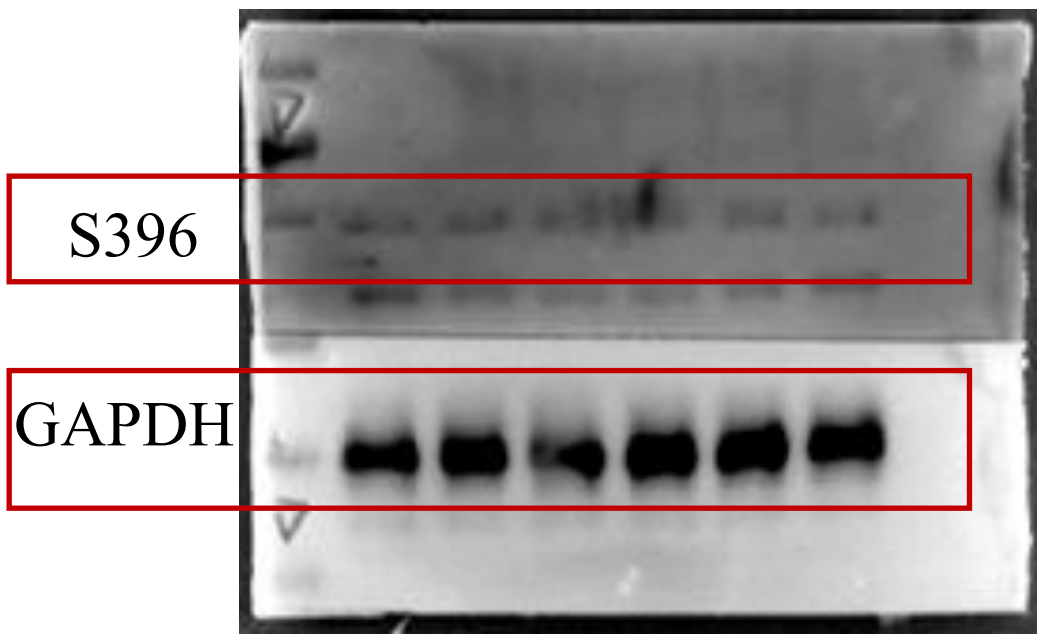

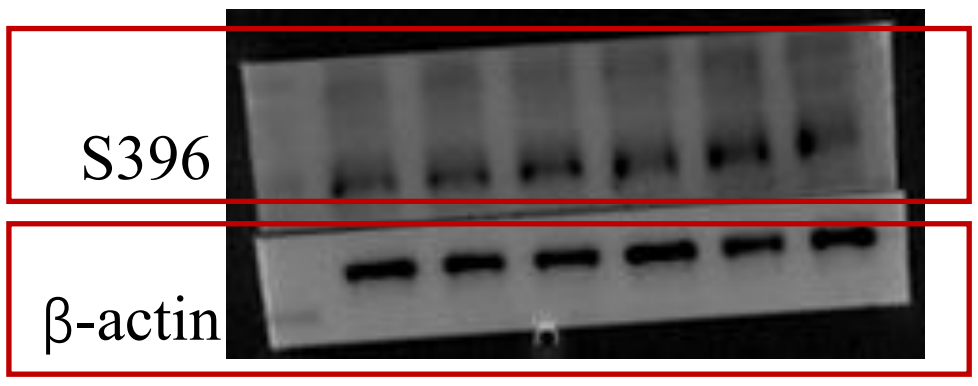

Fig. 3S-T205

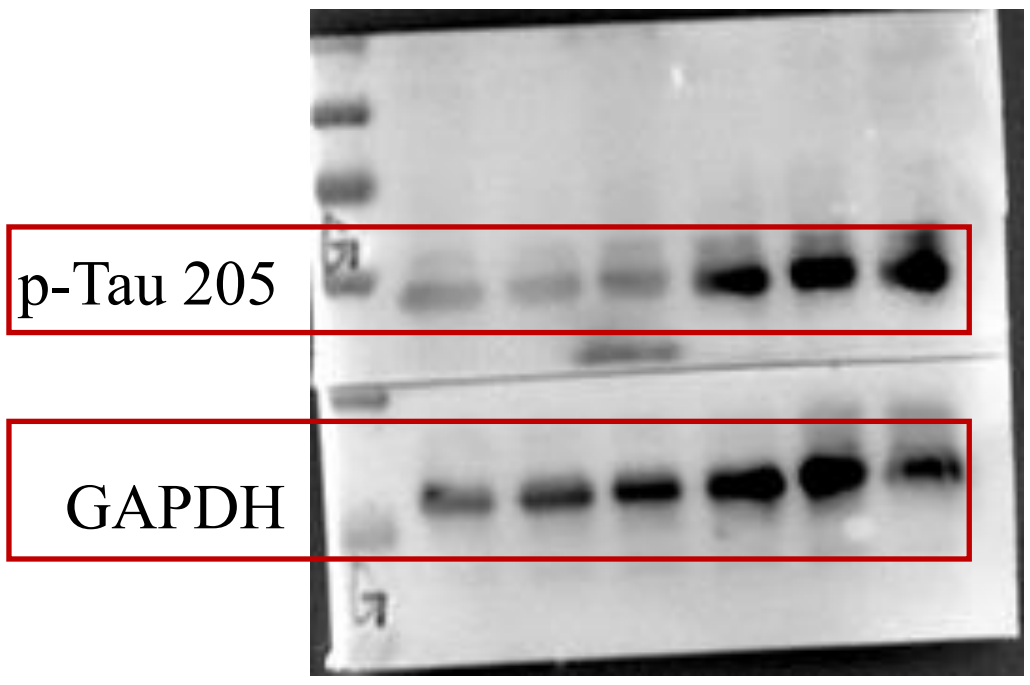

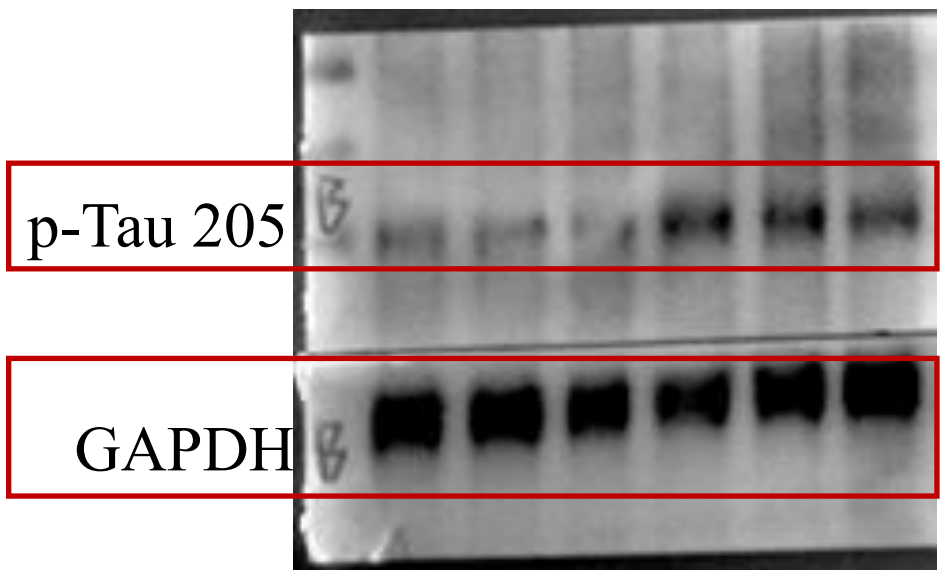

Fig. 3S-T231

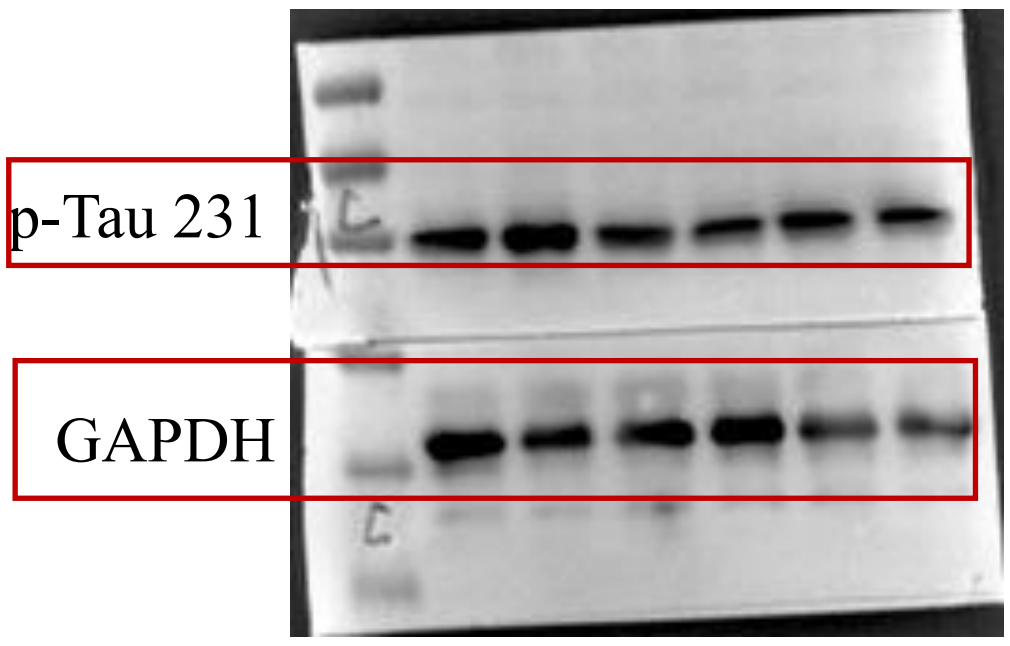

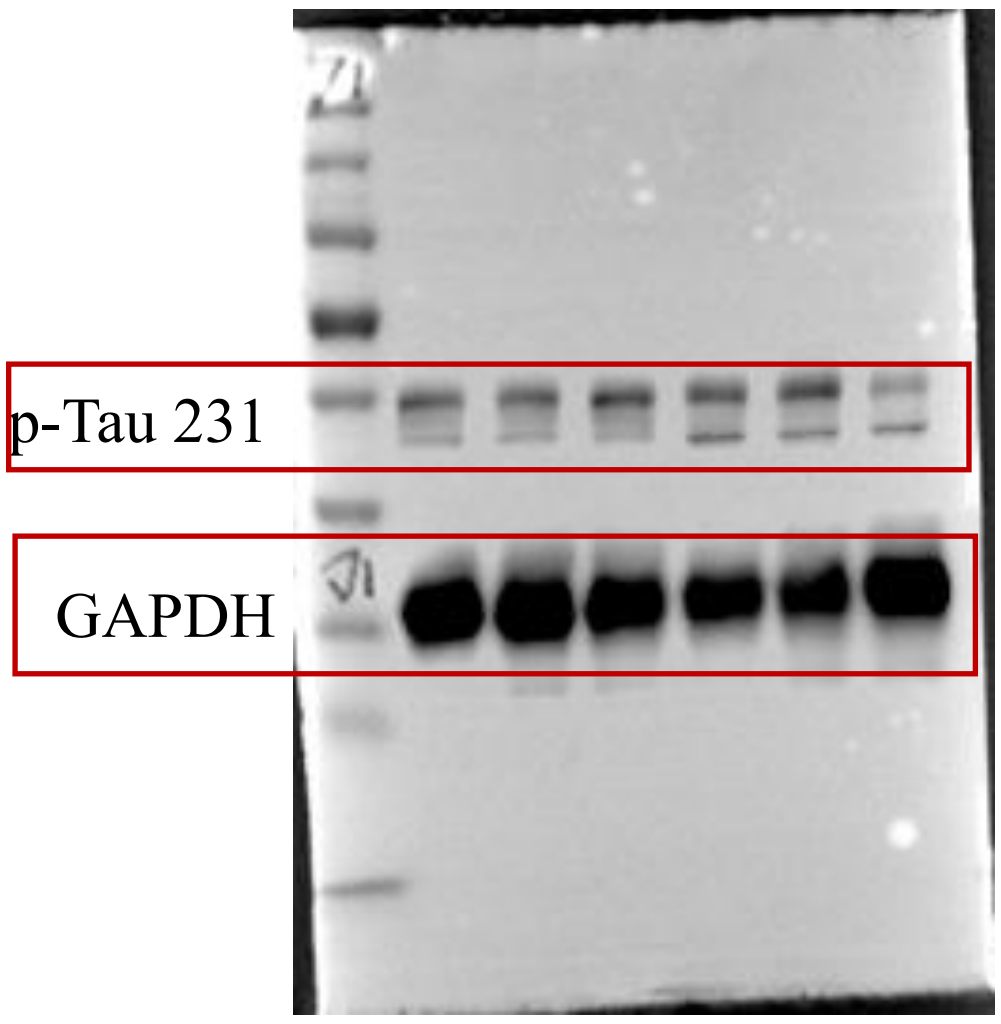

Fig. 3S-Tau

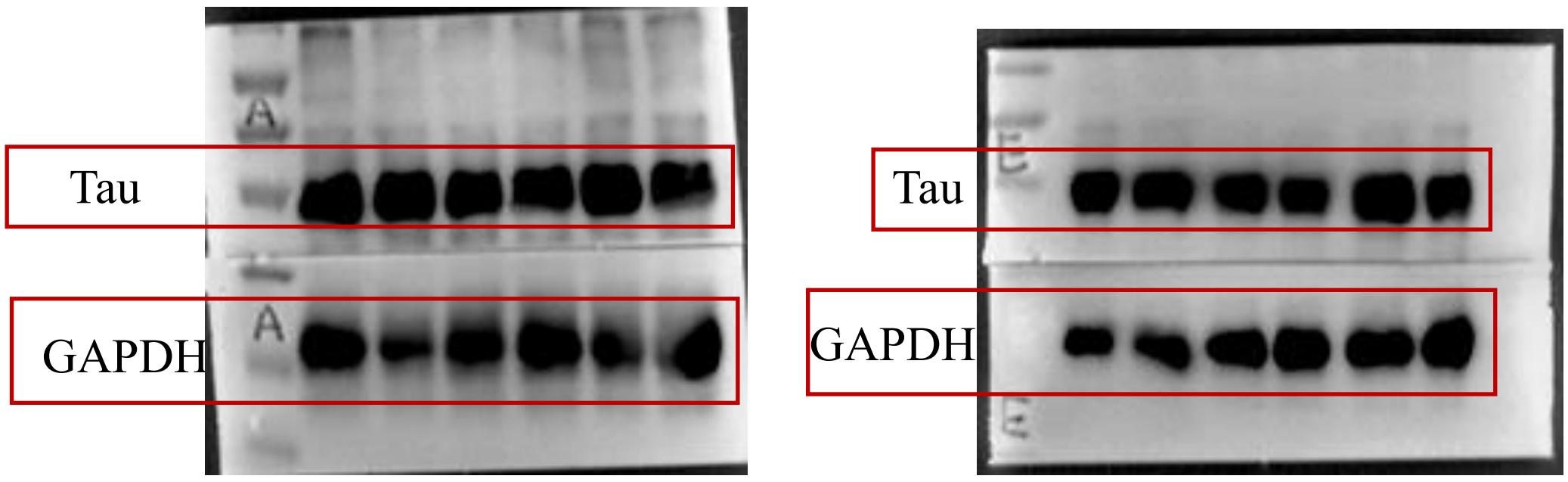

Fig. 4G

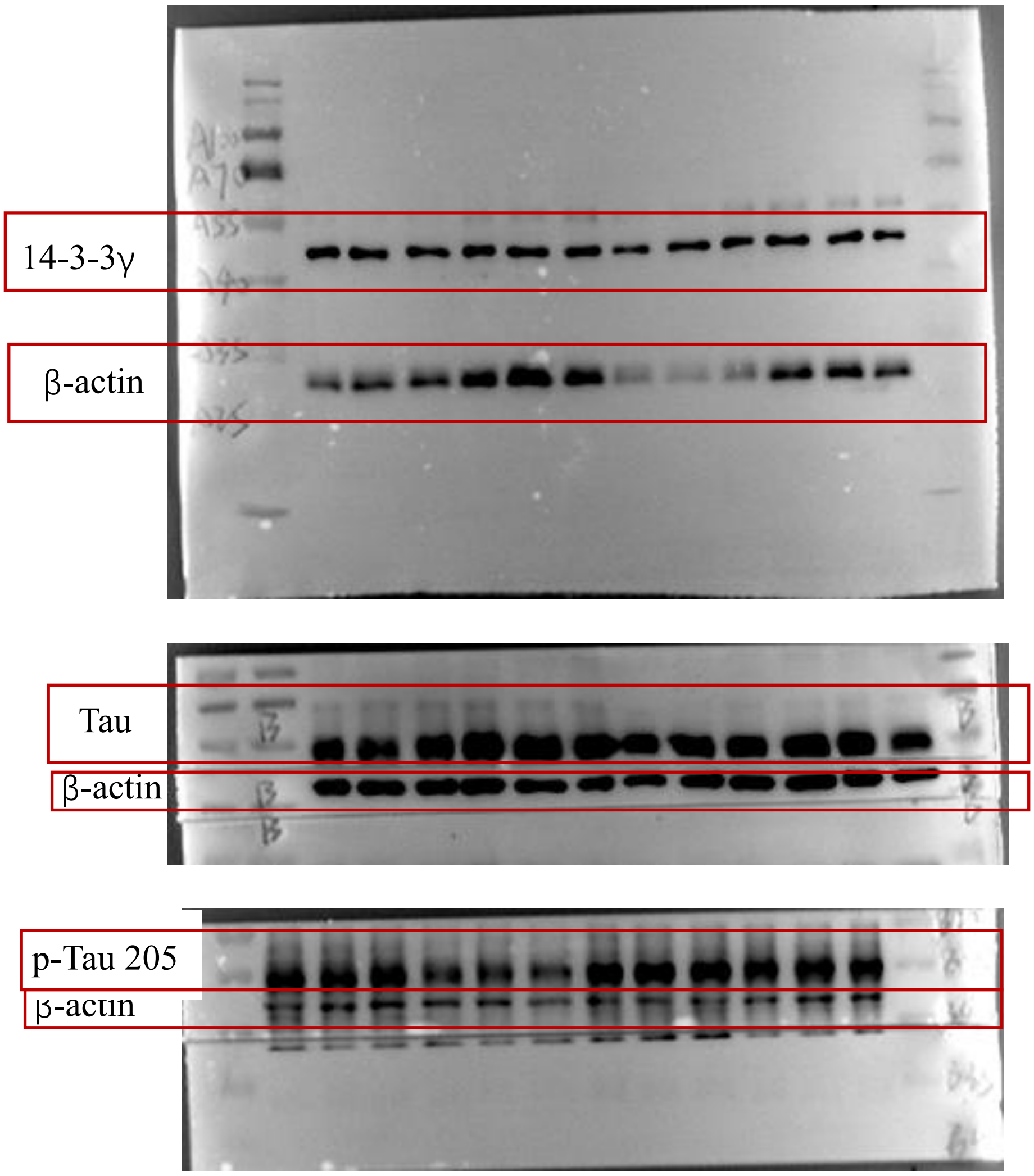

Fig. 4I

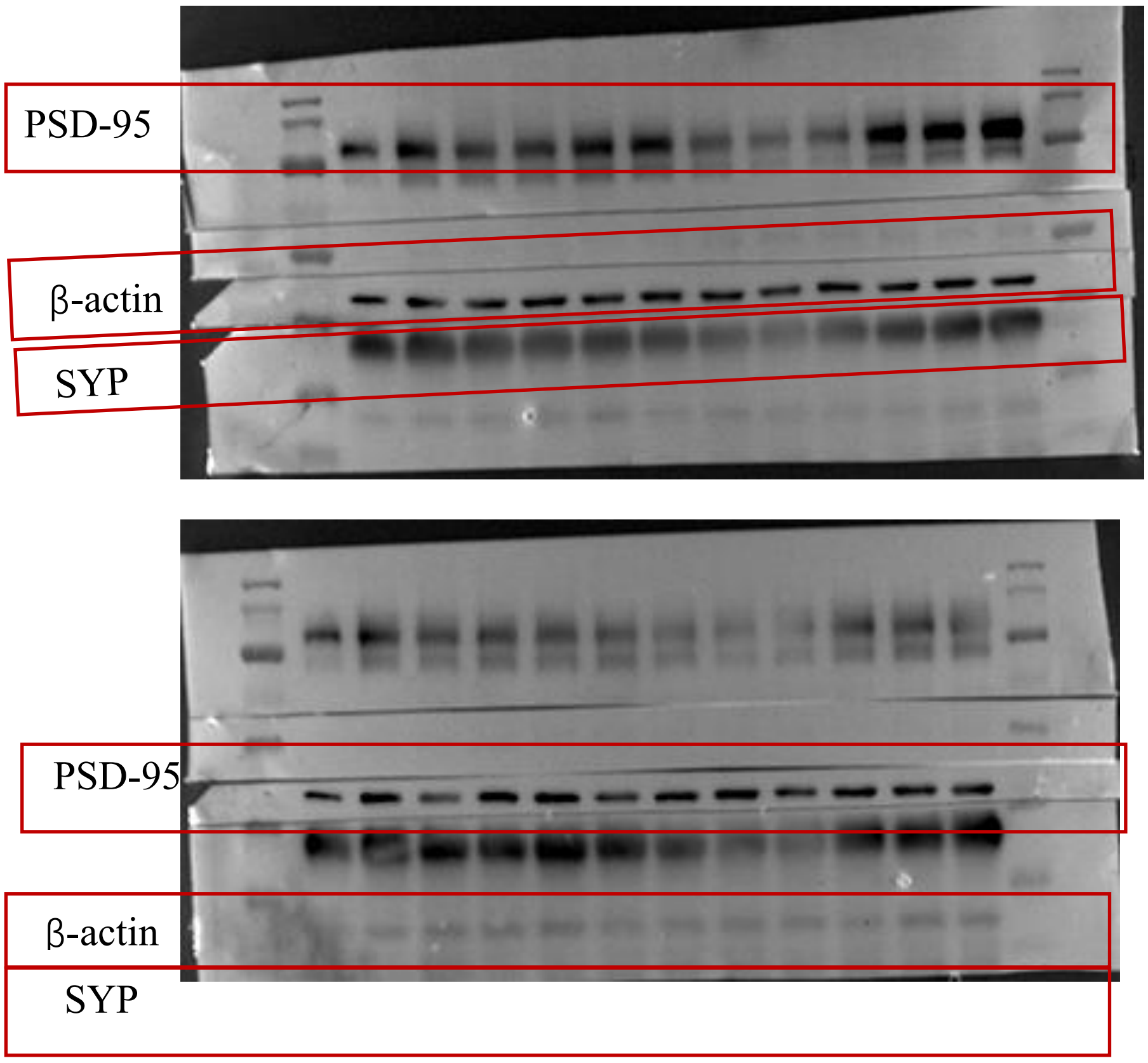
PSD-95
β-actin
SYP
PSD-95
β-actin
SYP

Fig. 5B

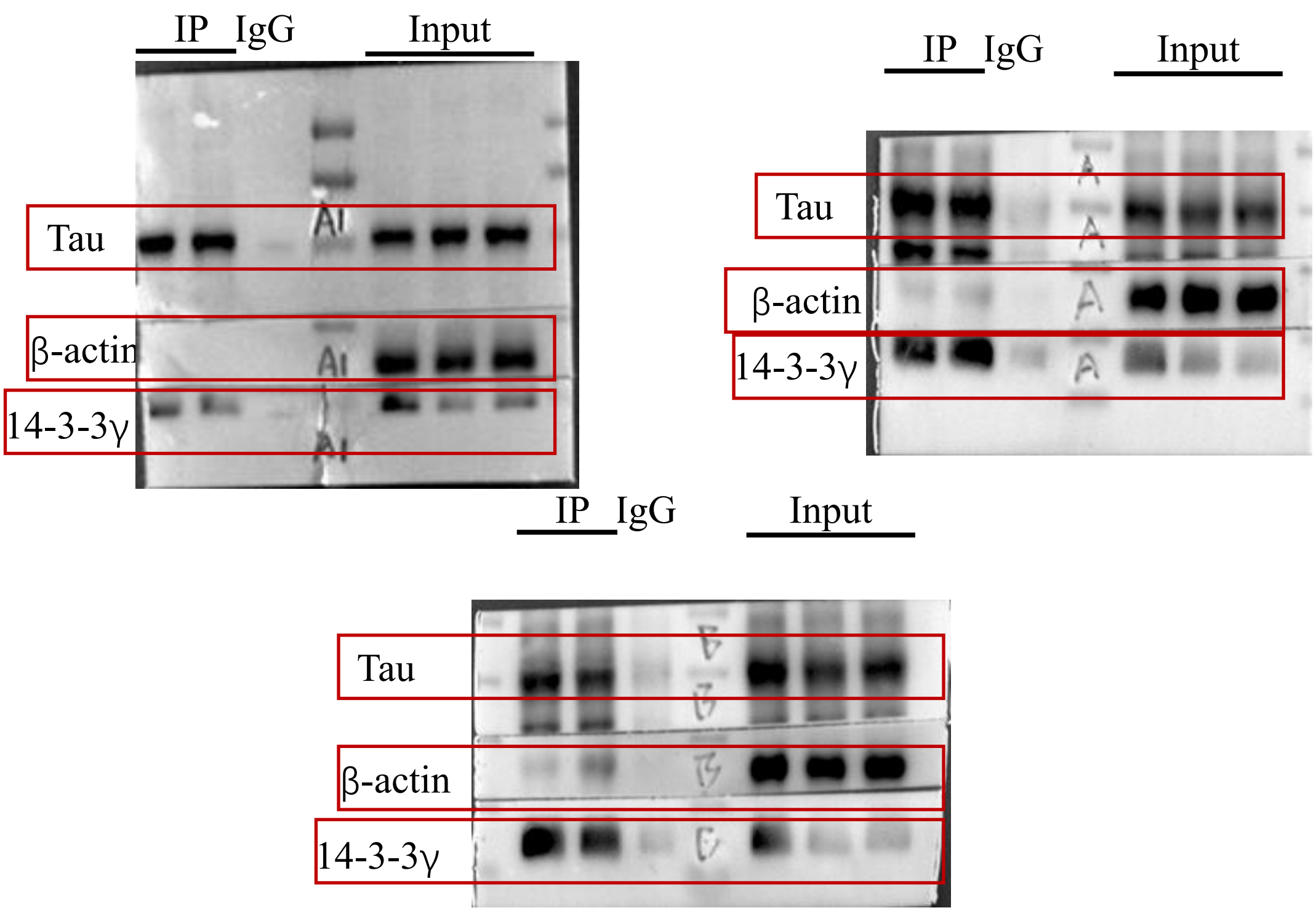
IP IgG
Input
Tau
β-actin
14-3-3γ
IP IgG
Input
Tau
β-actin
14-3-3γ
IP IgG
Input
Tau
β-actin
14-3-3γ

Fig. 5D

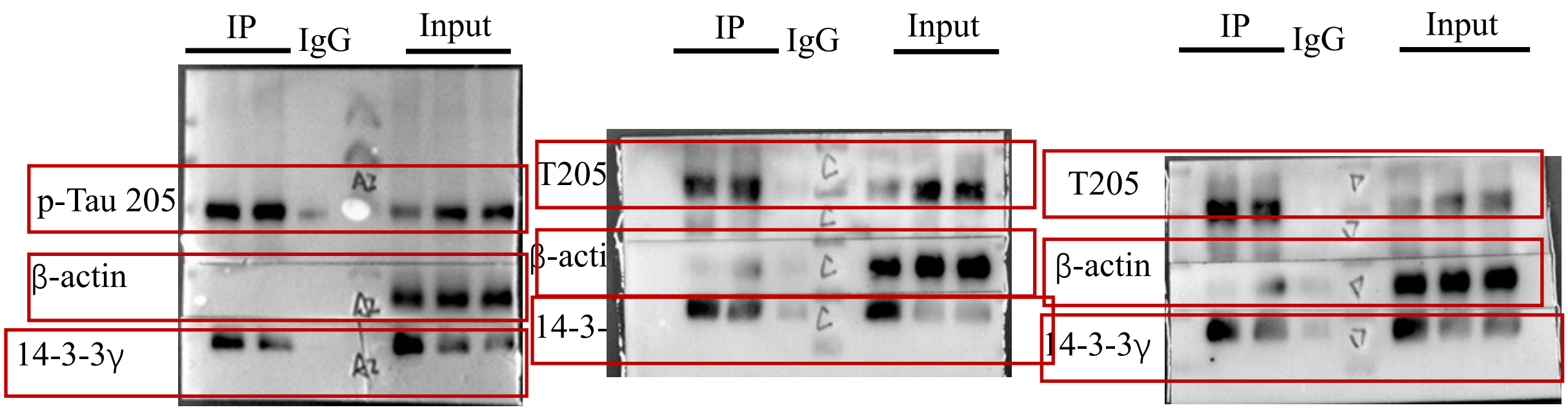
IP
IgG
Input
p-Tau 205
β-actin
14-3-3γ
IP
IgG
Input
T205
β-acti
14-3-
IP
IgG
Input
T205
β-actin
14-3-3γ

Fig. 5G

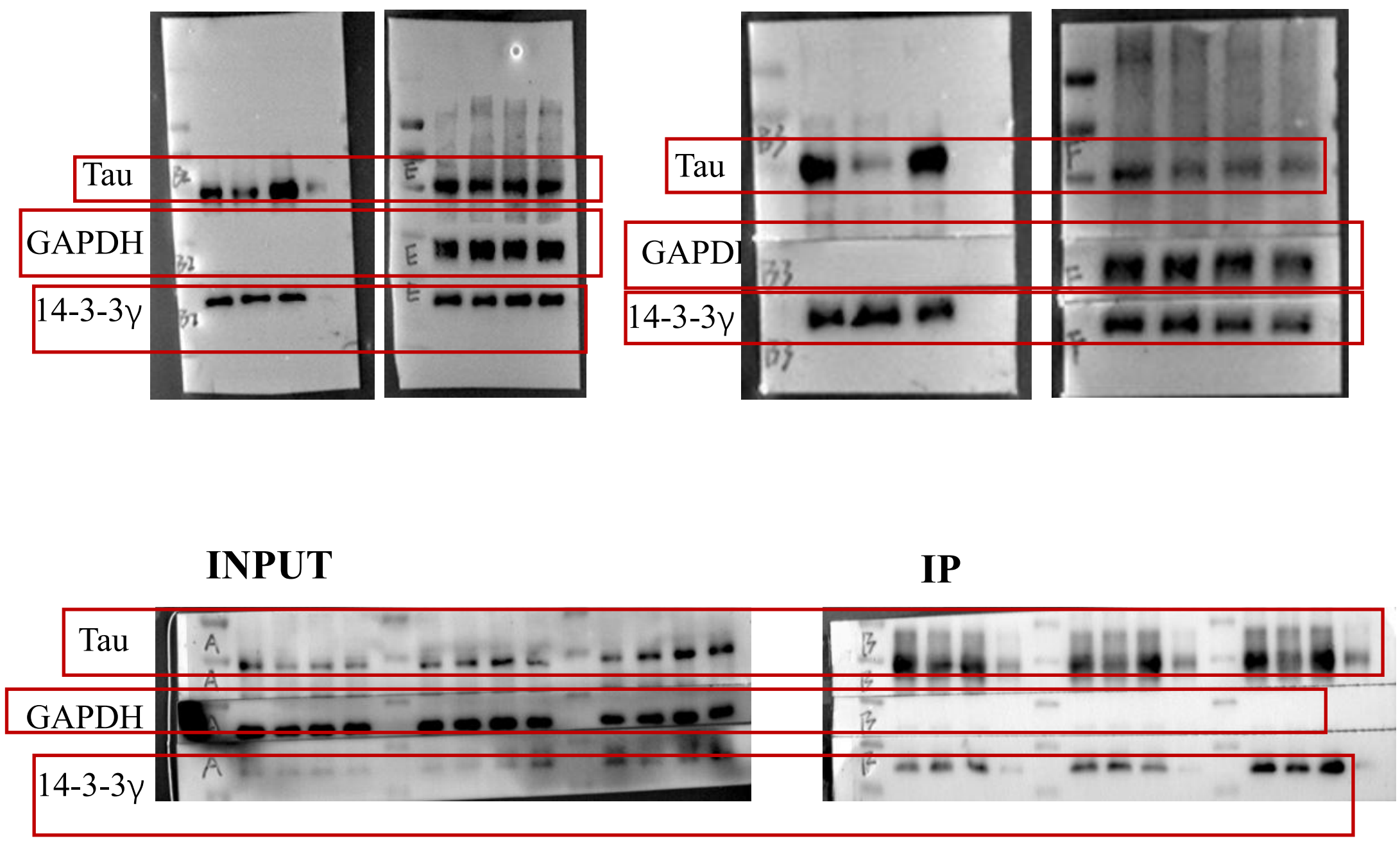
Tau
GAPDH
14-3-3γ
Tau
GAPDI
14-3-3γ
INPUT
IP
Tau
GAPDH
14-3-3γ

Fig. 6A

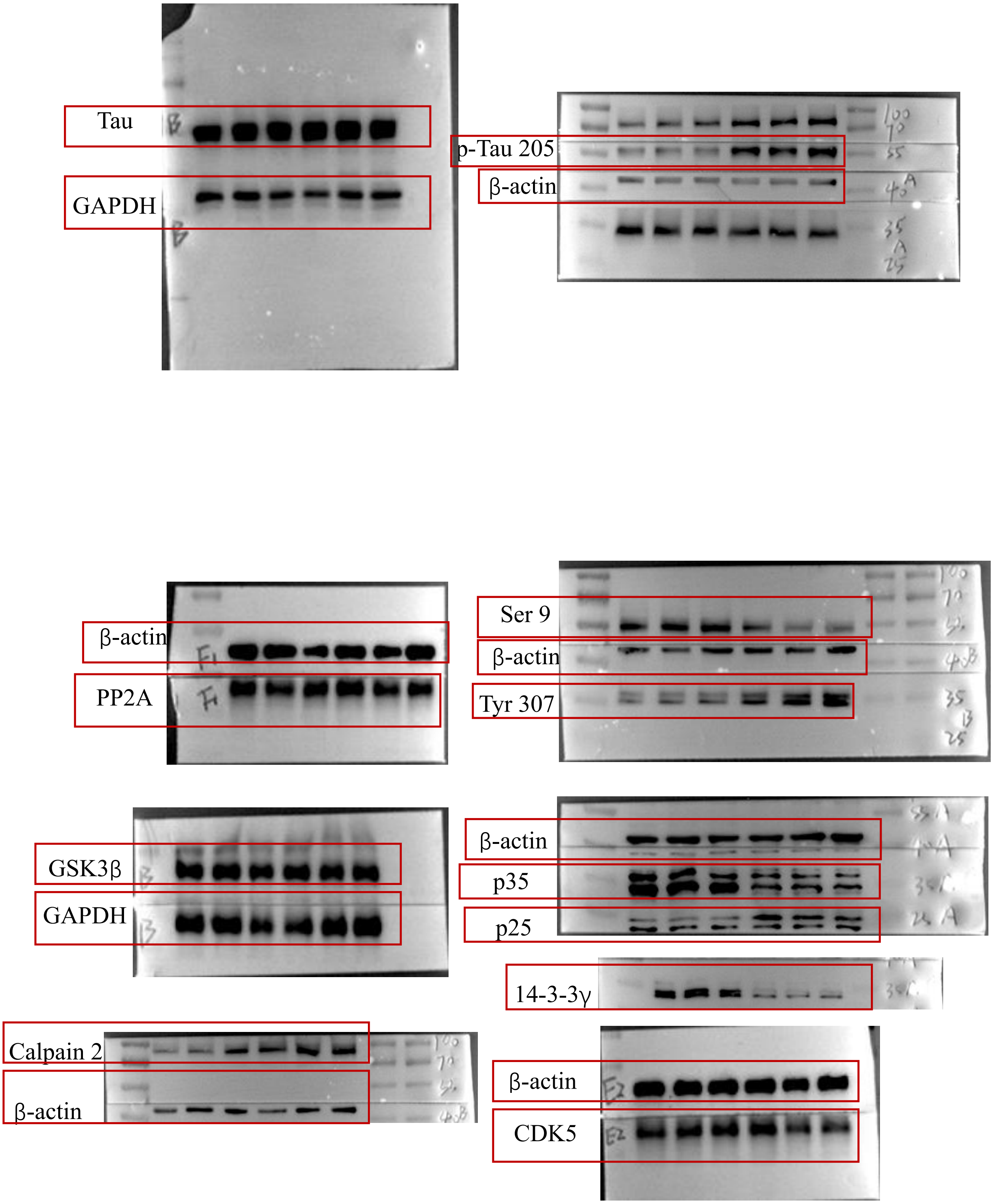
Tau
GAPDH
p-Tau 205
β-actin
β-actin
PP2A
Ser 9
β-actin
Tyr 307
GSK3β
GAPDH
β-actin
p35
p25
14-3-3γ
Calpain 2
β-actin
β-actin
CDK5

Fig. 6C-LiCL

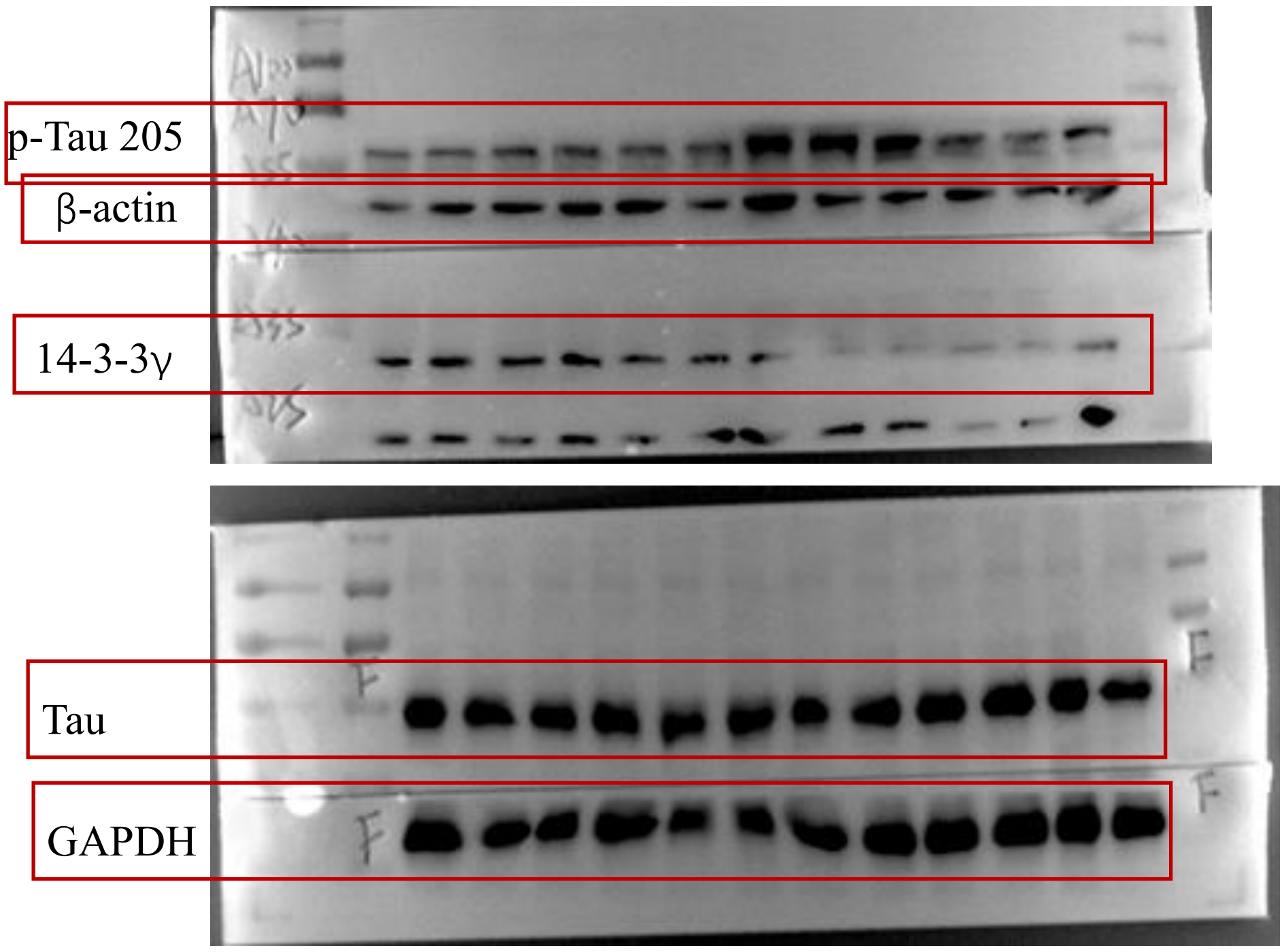

Fig. 6C-DT061

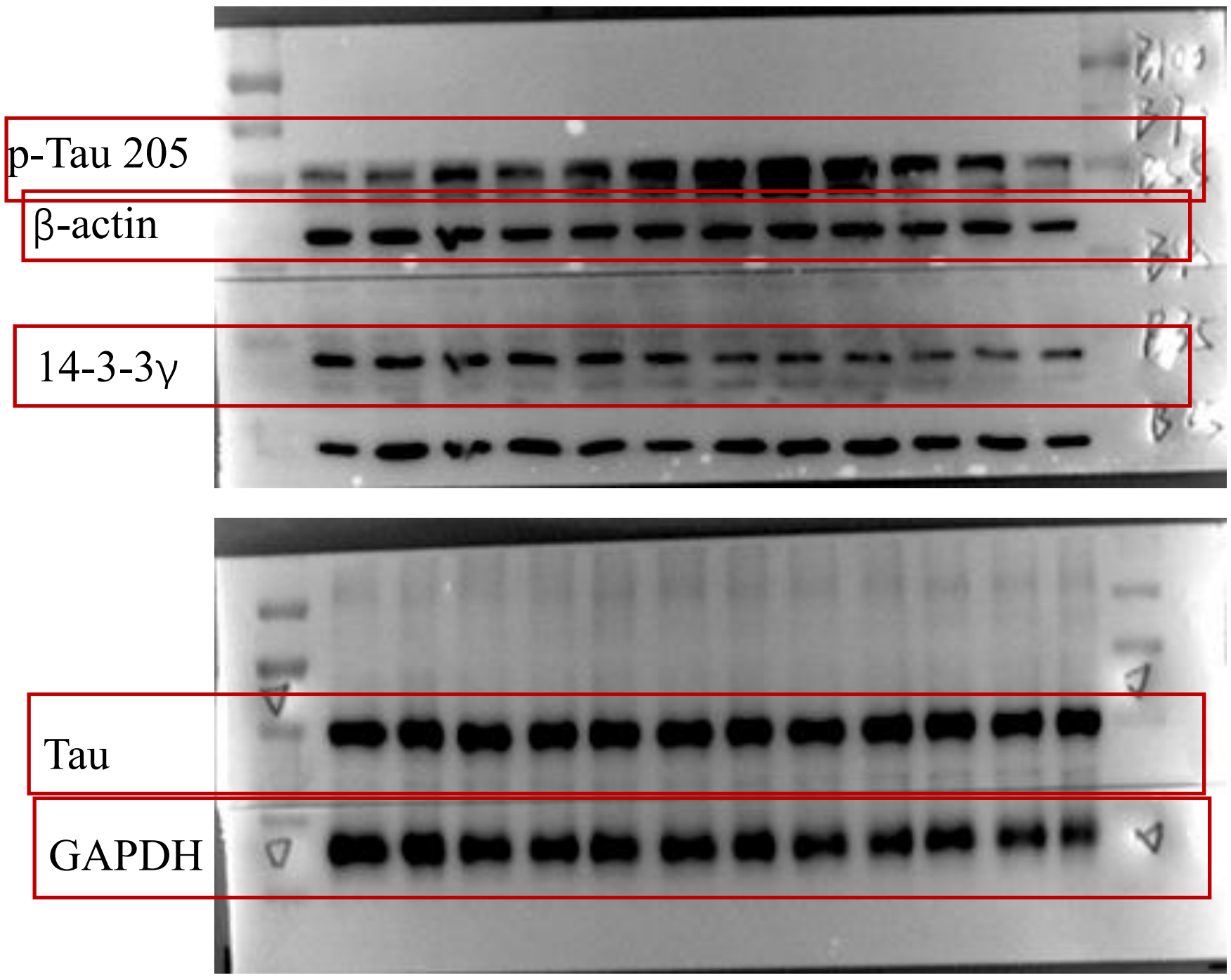

Fig. 6C-roscovitine

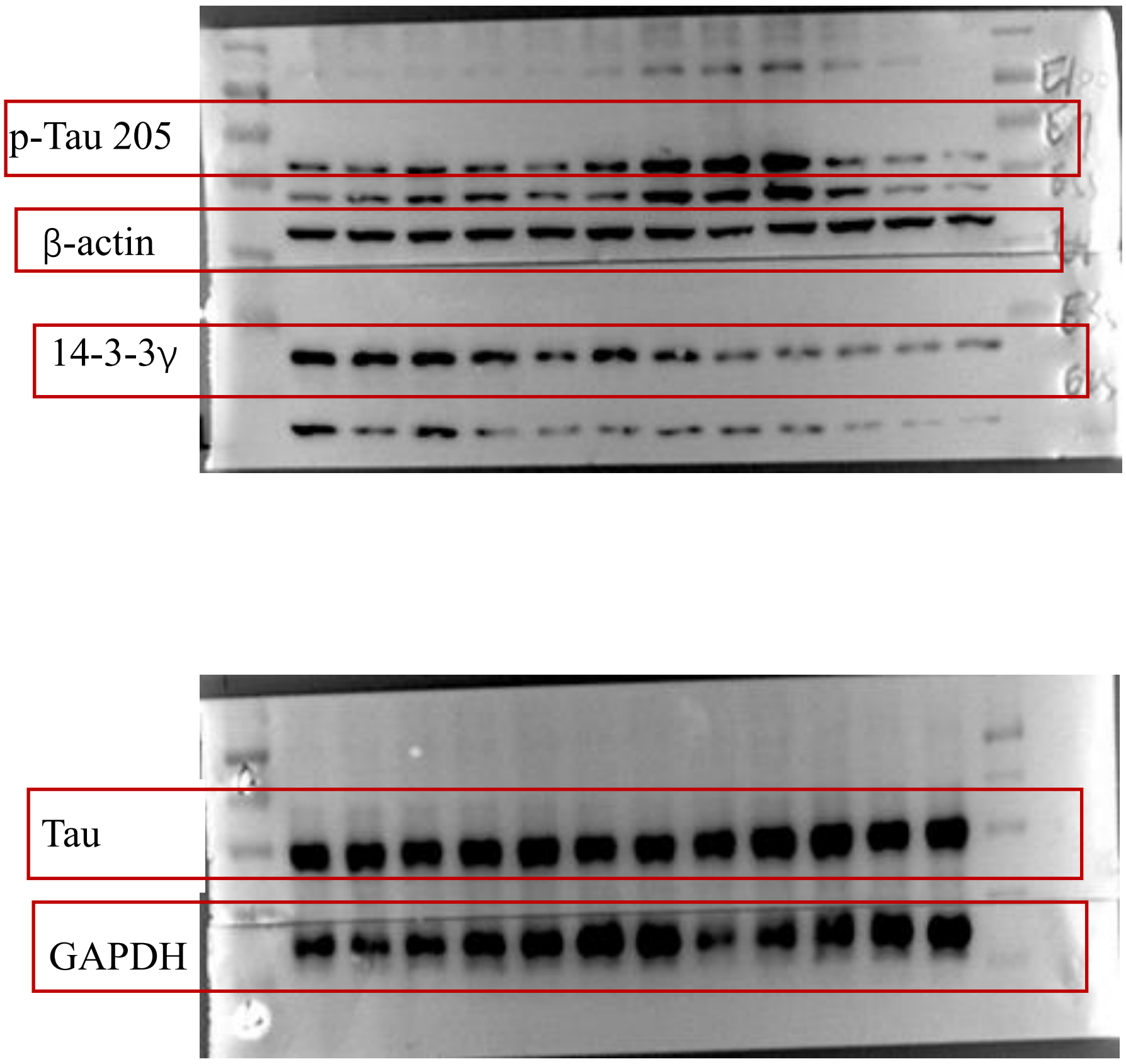

Fig. 6E

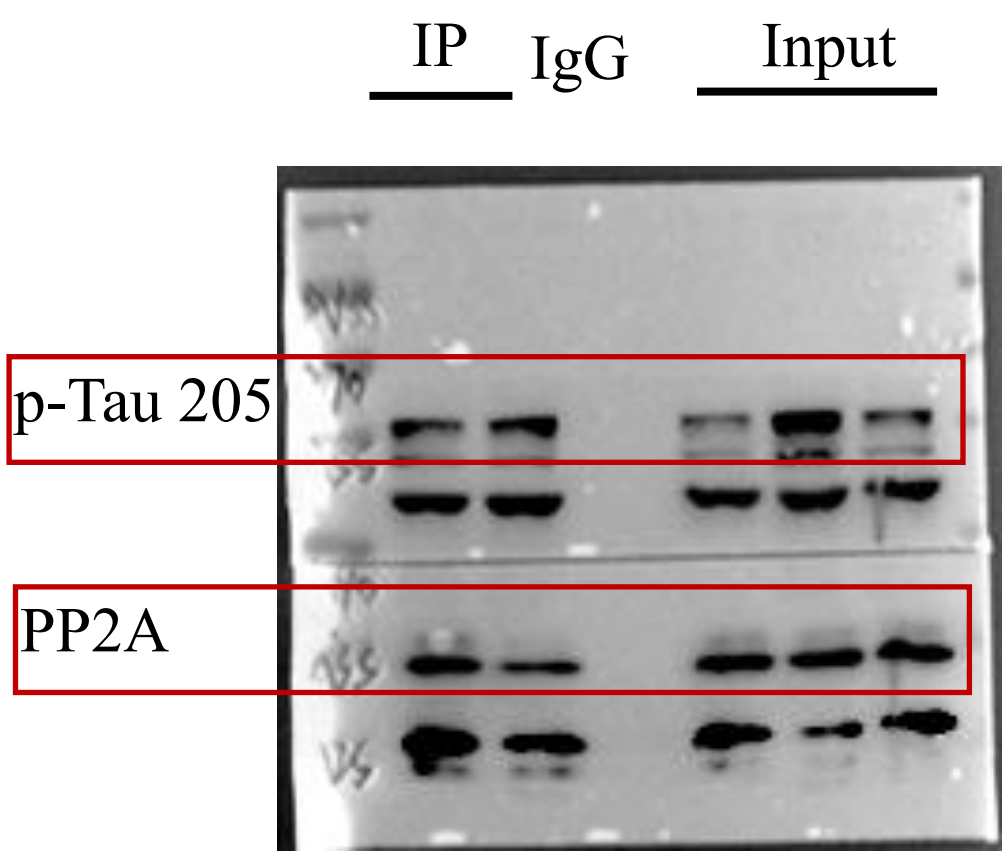

Fig. 6F

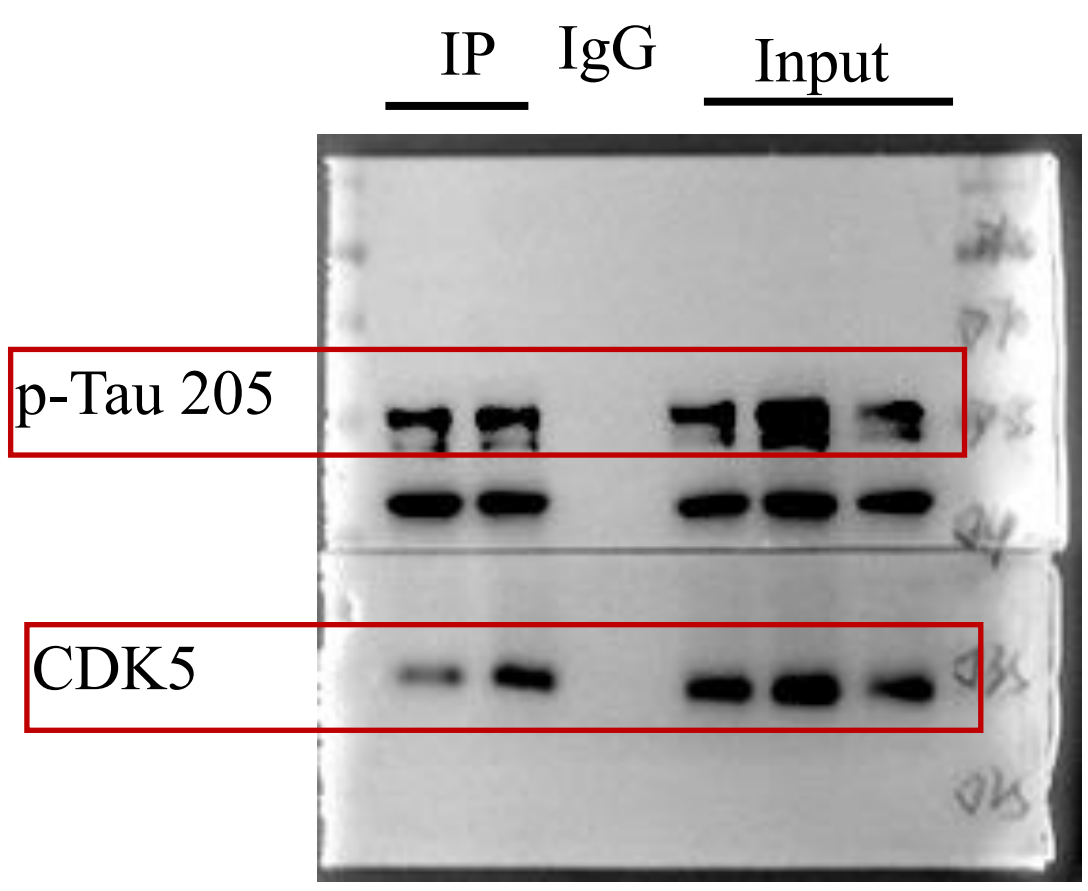

Fig. 6G

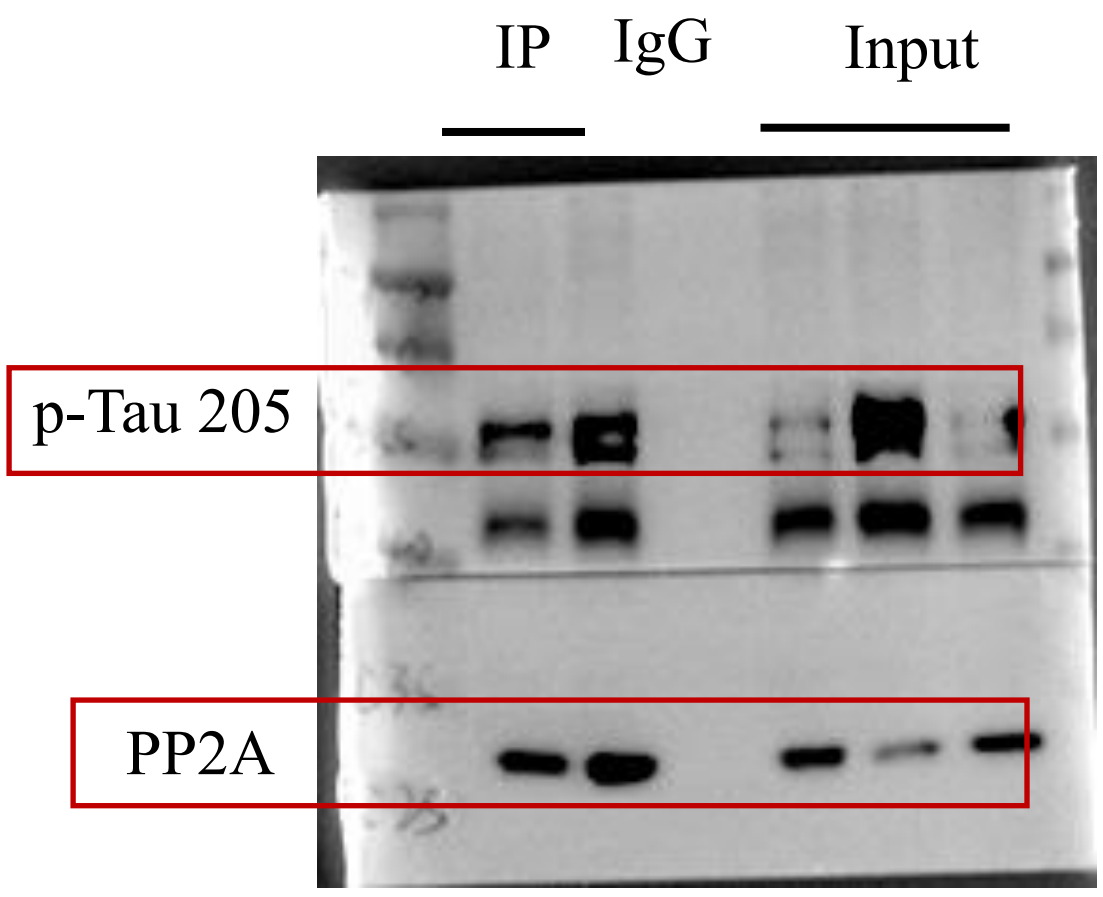
IP
IgG
Input
p-Tau 205
PP2A

Fig. 7B

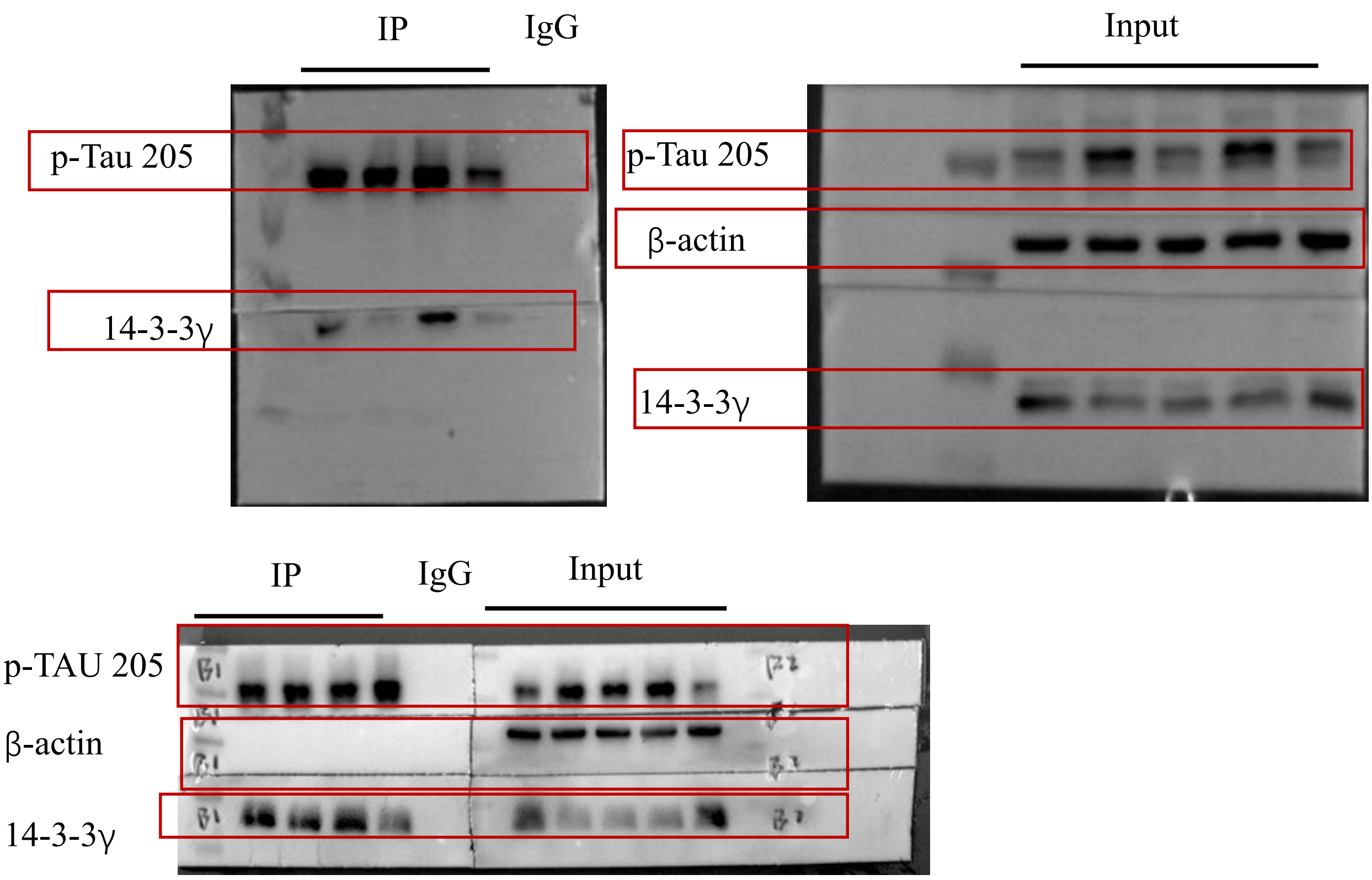
IP
IgG
Input
p-Tau 205
14-3-3γ
p-Tau 205
β-actin
14-3-3γ
IP
IgG
Input
p-TAU 205
β-actin
14-3-3γ

Fig. 7J

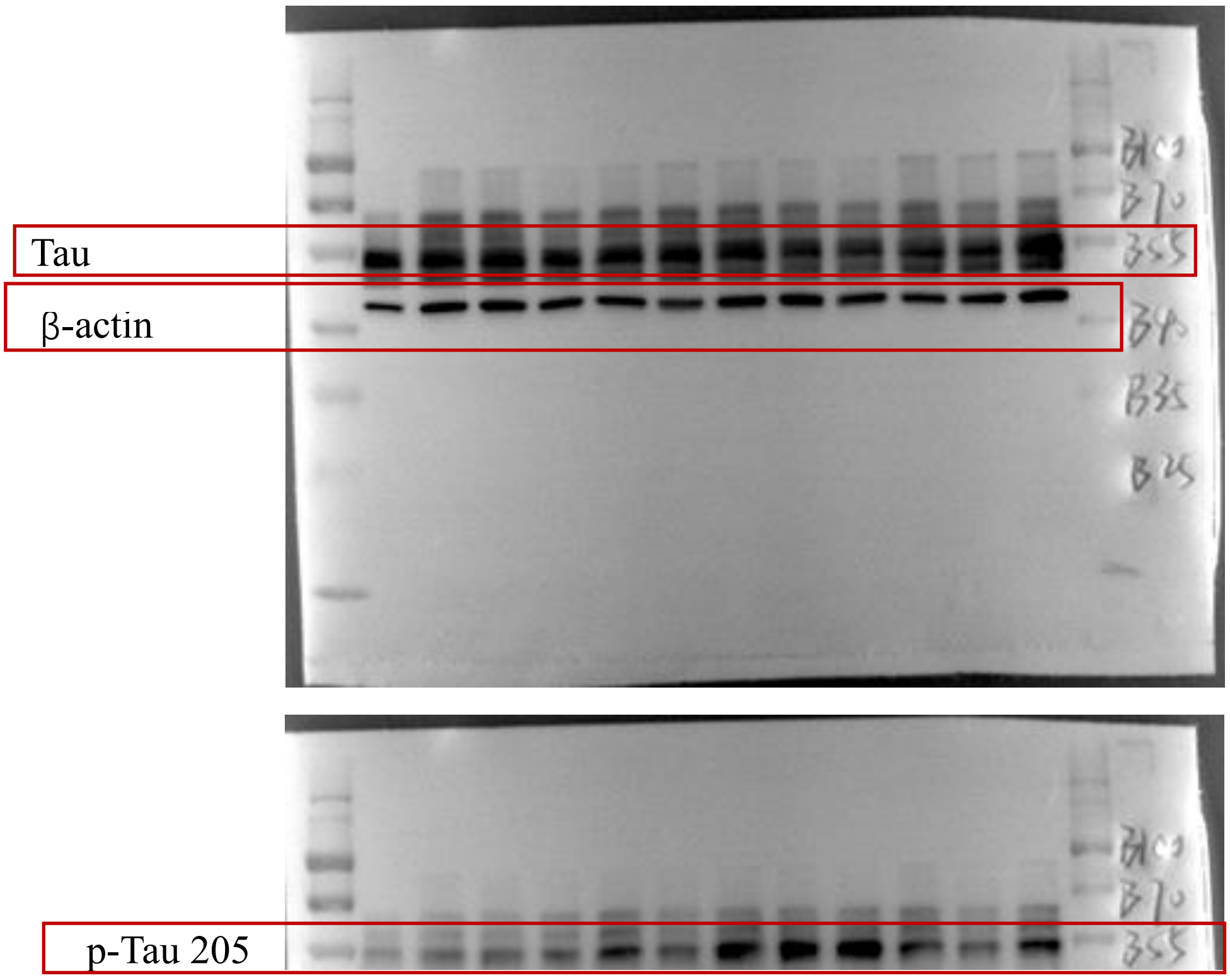

Fig. 7G

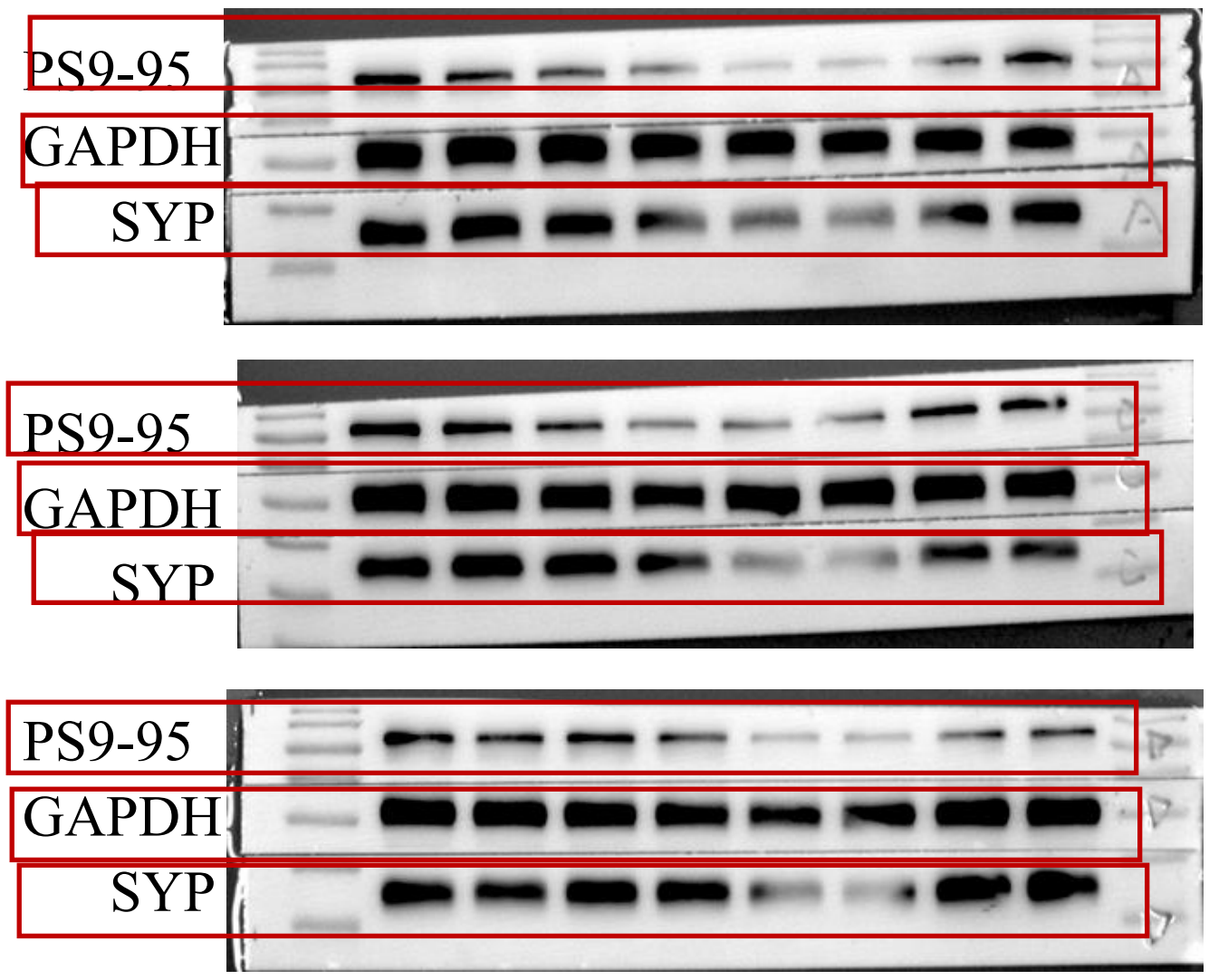

Supplement: Supplementary file 1 [file ADVS-9999-e77480-s001.pdf]
