# Supplementary material for: 14‐3‐3γ Protects Against Postoperative Cognitive Dysfunction by Regulating Tau Thr205 Phosphorylation and Synaptic Integrity
Source: Adv Sci (Weinh). 2026 Sep 27:e77480. Online ahead of print. doi: 10.1002/advs.77480 (PMC13616388; doi:10.1002/advs.77480)
Supplement: Supplementary file 2 [file ADVS-9999-e77480-s002.pdf]

Supplementary Materials

Table S1. Summary of study cohorts and their contributions to each analysis.

Cohort	N	Sample type	Assay/platform	Sampling time	Main purpose	Figure panels
ADNI proteomic discovery cohort	237	CSF	SomaScan 7K proteomics	Baseline	Protein discovery and candidate selection	Fig.1A-D
ADNI autopsy cohort	13	CSF	SomaScan 7K proteomics	Baseline/autopsy	Neuropathological validation	Fig.1E
PPMI external validation cohort	810	CSF	SomaScan 7K proteomics	Baseline	Independent validation of CSF 14-3-3γ	Fig.1F
ADNI longitudinal cohort	237	CSF + clinical/imaging data	CSF biomarkers, PET, cognitive assessments	Longitudinal follow-up	Association with cognitive decline and AD progression	Fig.1G-J
Prospective surgical cohort (Nanjing Drum Tower Hospital)	213	Plasma	ELISA (YWHAG/14-3-3γ)	Postoperative day 1	Clinical association and predictive modelling for POD/POCD	Fig.2A-L

Table S2. Cross-sectional associations between CSF 14-3-3γ levels and regional cortical amyloid burden (AV45 PET SUVR)

	Cross-sectional associations			
**ROI**	**beta**	**SE**	**t**	**FDR-adjusted *P***
L_bankssts	0.514	0.093	5.544	<0.001
L_caudalanteriorcingulate	0.511	0.109	4.668	<0.001
L_caudalmiddlefrontal	0.544	0.107	5.091	<0.001
L_cuneus	0.466	0.124	3.751	<0.001
L_entorhinal	0.347	0.112	3.096	0.003
L_frontalpole	0.577	0.107	5.416	<0.001
L_fusiform	0.586	0.102	5.735	<0.001
L_inferiorparietal	0.537	0.100	5.377	<0.001
L_inferiortemporal	0.552	0.100	5.543	<0.001
L_insula	0.513	0.102	5.027	<0.001
L_isthmuscingulate	0.537	0.102	5.293	<0.001
L_lateraloccipital	0.434	0.117	3.723	<0.001
L_lateralorbitofrontal	0.590	0.096	6.166	<0.001
L_lingual	0.498	0.117	4.271	<0.001
L_medialorbitofrontal	0.622	0.102	6.111	<0.001
L_middletemporal	0.514	0.104	4.918	<0.001
L_paracentral	0.528	0.102	5.151	<0.001
L_parahippocampal	0.443	0.110	4.031	<0.001
L_parsopercularis	0.579	0.103	5.604	<0.001
L_parsorbitalis	0.606	0.096	6.317	<0.001
L_parstriangularis	0.583	0.096	6.048	<0.001
L_pericalcarine	0.442	0.124	3.562	0.001
L_postcentral	0.543	0.108	5.031	<0.001
L_posteriorcingulate	0.572	0.097	5.902	<0.001
L_precentral	0.522	0.116	4.498	<0.001
L_precuneus	0.609	0.095	6.379	<0.001
L_rostralanteriorcingulate	0.576	0.100	5.742	<0.001
L_rostralmiddlefrontal	0.587	0.097	6.058	<0.001
L_superiorfrontal	0.570	0.101	5.672	<0.001
L_superiorparietal	0.536	0.103	5.191	<0.001
L_superiortemporal	0.552	0.100	5.539	<0.001
L_supramarginal	0.543	0.101	5.386	<0.001
L_temporalpole	0.484	0.125	3.868	<0.001
L_transversetemporal	0.503	0.104	4.836	<0.001
R_bankssts	0.545	0.101	5.413	<0.001
R_caudalanteriorcingulate	0.573	0.109	5.273	<0.001
R_caudalmiddlefrontal	0.574	0.104	5.495	<0.001
R_cuneus	0.498	0.135	3.687	0.001

R_entorhinal	0.339	0.125	2.713	0.009
R_frontalpole	0.623	0.103	6.025	<0.001
R_fusiform	0.578	0.112	5.150	<0.001
R_inferiorparietal	0.576	0.104	5.550	<0.001
R_inferiortemporal	0.562	0.107	5.261	<0.001
R_insula	0.551	0.107	5.132	<0.001
R_isthmuscingulate	0.495	0.106	4.680	<0.001
R_lateraloccipital	0.537	0.123	4.362	<0.001
R_lateralorbitofrontal	0.586	0.100	5.861	<0.001
R_lingual	0.595	0.121	4.905	<0.001
R_medialorbitofrontal	0.619	0.102	6.065	<0.001
R_middletemporal	0.568	0.108	5.259	<0.001
R_paracentral	0.545	0.112	4.866	<0.001
R_parahippocampal	0.420	0.125	3.363	0.001
R_parsopercularis	0.597	0.110	5.443	<0.001
R_parsorbitalis	0.554	0.096	5.799	<0.001
R_parstriangularis	0.555	0.103	5.404	<0.001
R_pericalcarine	0.589	0.125	4.697	<0.001
R_postcentral	0.538	0.114	4.720	<0.001
R_posteriorcingulate	0.583	0.107	5.448	<0.001
R_precentral	0.526	0.115	4.561	<0.001
R_precuneus	0.615	0.101	6.120	<0.001
R_rostralanteriorcingulate	0.564	0.104	5.445	<0.001
R_rostralmiddlefrontal	0.603	0.098	6.177	<0.001
R_superiorfrontal	0.606	0.102	5.953	<0.001
R_superiorparietal	0.596	0.105	5.687	<0.001
R_superiortemporal	0.543	0.103	5.271	<0.001
R_supramarginal	0.552	0.105	5.238	<0.001
R_temporalpole	0.430	0.126	3.400	0.001
R_transversetemporal	0.545	0.113	4.844	<0.001

Note: β values represent standardized regression coefficients derived from linear models adjusted for age, sex, education, and APOE ε4 carrier status. AV45 PET SUVR indicates cortical amyloid burden measured by florbetapir PET imaging. Multiple comparisons across regions of interest (ROIs) were corrected using the Bonferroni method. Numerical values were rounded to an appropriate number of decimal places. P values <0.001 are presented as <0.001.

Table S3. Longitudinal associations of CSF 14-3-3γ with core Alzheimer's disease biomarkers and cognitive performance

Longitudinal associations				
**ROI**	**beta**	**SE**	**t**	**FDR-adjusted *P***
L_bankssts	8.90E-05	8.82E-05	1.009	0.314
L_caudalanteriorcingulate	4.50E-05	9.88E-05	0.455	0.649
L_caudalmiddlefrontal	1.16E-04	8.61E-05	1.344	0.180
L_cuneus	1.29E-04	9.93E-05	1.300	0.195
L_entorhinal	3.45E-04	1.51E-04	2.279	0.023
L_frontalpole	1.89E-04	9.81E-05	1.931	0.054
L_fusiform	1.59E-04	9.22E-05	1.722	0.086
L_inferiorparietal	1.55E-04	8.82E-05	1.763	0.079
L_inferiortemporal	1.22E-04	9.34E-05	1.305	0.193
L_insula	9.00E-05	9.87E-05	0.912	0.362
L_isthmuscingulate	1.51E-04	9.69E-05	1.559	0.120
L_lateraloccipital	2.13E-04	1.11E-04	1.910	0.057
L_lateralorbitofrontal	1.45E-04	9.39E-05	1.542	0.124
L_lingual	1.91E-04	1.02E-04	1.876	0.062
L_medialorbitofrontal	8.62E-05	8.90E-05	0.969	0.333
L_middletemporal	1.08E-04	9.06E-05	1.189	0.236
L_paracentral	2.06E-04	1.04E-04	1.989	0.048
L_parahippocampal	1.84E-04	1.16E-04	1.590	0.113
L_parsopercularis	1.01E-04	8.91E-05	1.136	0.257
L_parsorbitalis	9.73E-05	1.02E-04	0.958	0.339
L_parstriangularis	6.81E-05	9.25E-05	0.736	0.462
L_pericalcarine	1.97E-04	1.13E-04	1.749	0.081
L_postcentral	1.49E-04	9.58E-05	1.558	0.120
L_posteriorcingulate	1.28E-04	8.96E-05	1.426	0.155
L_precentral	1.23E-04	1.01E-04	1.213	0.226
L_precuneus	1.36E-04	7.88E-05	1.727	0.085
L_rostralanteriorcingulate	2.70E-05	8.56E-05	0.315	0.753
L_rostralmiddlefrontal	1.46E-04	8.04E-05	1.811	0.071
L_superiorfrontal	1.19E-04	7.76E-05	1.528	0.128
L_superiorparietal	1.38E-04	8.89E-05	1.555	0.121
L_superiortemporal	6.49E-05	8.84E-05	0.734	0.463
L_supramarginal	1.42E-04	8.72E-05	1.627	0.105
L_temporalpole	2.12E-04	1.14E-04	1.855	0.065
L_transversetemporal	-2.36E-05	9.55E-05	-0.247	0.805
R_bankssts	7.58E-05	9.32E-05	0.814	0.416
R_caudalanteriorcingulate	3.56E-05	9.50E-05	0.374	0.709
R_caudalmiddlefrontal	6.75E-05	8.72E-05	0.774	0.439
R_cuneus	1.10E-04	1.02E-04	1.073	0.284

R_entorhinal	1.91E-04	1.60E-04	1.188	0.236
R_frontalpole	1.63E-04	1.01E-04	1.613	0.108
R_fusiform	1.80E-04	1.01E-04	1.787	0.075
R_inferiorparietal	1.30E-04	9.01E-05	1.439	0.151
R_inferiortemporal	1.32E-04	9.57E-05	1.379	0.169
R_insula	7.40E-05	9.77E-05	0.757	0.450
R_isthmuscingulate	1.01E-04	1.02E-04	0.986	0.325
R_lateraloccipital	2.10E-04	1.02E-04	2.050	0.041
R_lateralorbitofrontal	5.23E-05	1.00E-04	0.521	0.603
R_lingual	1.64E-04	1.02E-04	1.614	0.108
R_medialorbitofrontal	5.99E-05	8.34E-05	0.719	0.473
R_middletemporal	8.29E-05	9.36E-05	0.885	0.377
R_paracentral	1.99E-04	1.09E-04	1.826	0.069
R_parahippocampal	1.42E-04	1.18E-04	1.202	0.230
R_parsopercularis	7.42E-05	9.08E-05	0.817	0.415
R_parsorbitalis	6.47E-05	9.87E-05	0.655	0.513
R_parstriangularis	4.86E-05	9.36E-05	0.519	0.604
R_pericalcarine	8.33E-05	1.08E-04	0.769	0.443
R_postcentral	1.42E-04	9.65E-05	1.472	0.142
R_posteriorcingulate	1.14E-04	8.77E-05	1.296	0.196
R_precentral	1.24E-04	1.01E-04	1.223	0.222
R_precuneus	1.29E-04	8.77E-05	1.471	0.143
R_rostralanteriorcingulate	4.49E-06	8.62E-05	0.052	0.958
R_rostralmiddlefrontal	1.37E-04	8.21E-05	1.665	0.097
R_superiorfrontal	9.82E-05	7.96E-05	1.234	0.218
R_superiorparietal	1.21E-04	8.85E-05	1.364	0.174
R_superiortemporal	1.03E-04	9.05E-05	1.135	0.257
R_supramarginal	1.49E-04	9.33E-05	1.592	0.112
R_temporalpole	1.49E-04	1.15E-04	1.295	0.196
R_transversetemporal	-3.64E-05	1.03E-04	-0.352	0.725

Note: β values represent standardized regression coefficients derived from linear models adjusted for age, sex, education, and APOE ε4 carrier status. AV45 PET SUVR indicates cortical amyloid burden measured by florbetapir PET imaging. Multiple comparisons across regions of interest (ROIs) were corrected using the Bonferroni method. Numerical values were rounded to an appropriate number of decimal places. P values <0.001 are presented as <0.001.

Table S4. Cross-sectional and longitudinal associations of CSF 14-3-3γ with core Alzheimer's disease biomarkers and cognitive trajectories

Cognition endophenotypes	Cross-sectional associations			
	Beta	SE	t value	P value
CSF Aβ42	-0.451	0.054	-8.336	<0.001
CSF p-tau181	0.861	0.033	25.888	<0.001
CSF t-tau	0.885	0.033	26.906	<0.001
AV45 PET SUVR	0.536	0.063	8.523	<0.001
Hippocampal volume	-0.565	0.103	-5.489	<0.001
FDG PET SUVR	-0.444	0.077	-5.777	<0.001
Executive function	-0.232	0.101	-2.303	0.004
Memory	-0.336	0.102	-3.293	<0.001
Global cognition	-0.450	0.067	-6.721	<0.001
**Cognition endophenotypes**	**Longitudinal associations**			
	**Beta**	**SE**	**t value**	**P value**
CSF Aβ42	-1.91E-05	2.02E-04	-9.44E-02	0.154
CSF p-tau181	1.91E-05	5.39E-06	3.54E+00	<0.001
CSF t-tau	2.26E-04	5.21E-05	4.33E+00	<0.001
AV45 PET SUVR	2.76E-07	6.65E-08	4.147	<0.001
Hippocampal volume	-1.71E-03	1.59E-03	-1.07E+00	0.048
FDG PET SUVR	-4.90E-07	1.01E-07	-4.84E+00	<0.001
Executive function	-2.87E-06	3.15E-07	-9.112	<0.001
Memory	-3.68E-06	3.02E-07	-1.22E+01	<0.001
Global cognition	-2.18E-05	1.49E-06	-1.46E+01	<0.001

Notes: Cross-sectional associations were assessed using multivariable linear regression models, and longitudinal associations were evaluated using linear mixed-effects models with random intercepts. β values represent standardized effect sizes. All models were adjusted for age, sex, education level and APOE ε4 carrier status. CSF biomarkers include amyloid-β 42 (Aβ42), phosphorylated tau (p-tau181), and total tau (t-tau). AV45 PET SUVR reflects cortical amyloid burden, whereas FDG PET SUVR represents cerebral glucose metabolism. Hippocampal volume was derived from structural MRI. Cognitive domains include executive function, memory, and global cognition. For longitudinal analyses, β values represent the association between baseline CSF 14-3-3γ and the rate of change in each outcome over time. All p values are two-sided. Numerical values were rounded to an appropriate number of decimal places. P values <0.001 are presented as <0.001.

Table S5. Detailed information of antibodies, pharmacological agents, and key reagents used in this study

Category	Item/Target	Application	Species	Dilution/Concentration	Vendor	Catalog Number
Primary antibody	14-3-3γ	WB / IF / Co-IP	Rabbit/Mouse	1:1000/1:200/1:200	Affinity	DF3493/BF9438
Primary antibody	p-Tau (Thr205)	WB / IF / Co-IP	Rabbit	1:1000/1:200/1:200	CST	49561
Primary antibody	Total Tau	WB	Rabbit	1:1000	Proteintech	10274-1-AP
Primary antibody	AT8 (Ser202/Thr205)	WB	Rabbit	1:1000	Proteintech	82568-1-RR
Primary antibody	p-Tau (Thr231)	WB	Rabbit	1:1000	ABclonal	AP0053
Primary antibody	p-Tau (Ser396)	WB	Mouse	1:1000	CST	9632
Primary antibody	p-Tau (Ser262)	WB	Rabbit	1:1000	ABclonal	AP0397
Primary antibody	PSD95	WB / IF	Mouse	1:1000	CST	36233
Primary antibody	Synaptophysin (SYP)	WB / IF	Rabbit	1:1000	Proteintech	17785-1-AP
Primary antibody	β-actin	WB	Mouse	1:50000	Proteintech	66009-1-Ig
Primary antibody	β-tubulin	WB	Rabbit	1:2000	Proteintech	10094-1-AP
Primary antibody	GAPDH	WB	Rabbit	1:50000	Proteintech	60004-1-Ig
Primary antibody	NeuN	IF	Mouse	1:1000	Proteintech	26975-1-AP
Primary antibody	CDK5	WB	Rabbit	1:1000	ABclonal	A26254
Primary antibody	p25/p35	WB	Rabbit	1:1000	ABclonal	A24395
Primary antibody	GSK-3β	WB	Rabbit	1:1000	Proteintech	82061-1-RR
Primary antibody	p-GSK-3β (Ser9)	WB	Rabbit	1:1000	Proteintech	67558-1-lg
Primary antibody	PP2A	WB	Rabbit	1:1000	ABclonal	A6702
Primary antibody	p-PP2A (Tyr307)	WB	Rabbit	1:1000	ABclonal	AP1043

Secondary antibody	Anti-rabbit IgG (HRP)	WB	/	1:5000	beyotime	A0408
Secondary antibody	Anti-mouse IgG (HRP)	WB	/	1:5000	beyotime	A0412
Secondary antibody	Fluorophore-conjugated secondary antibodies	IF	/	1:500	Proteintech	SA00013-2
Small molecule	FC-A	Treatment	/	5mg/kg	Merck	20108-30-9
Small molecule	BV02	Treatment	/	10mg/kg	MCE	HY-101985
Small molecule	Roscovitine (CDK5 inhibitor)	Cell treatment	/	0.2uM	MCE	HY-30237
Small molecule	LiCl (GSK-3β inhibitor)	Cell treatment	/	5mM	MCE	HY-Y0649
Small molecule	DT061 (PP2A activator)	Cell treatment	/	14.3uM	MCE	HY-112929
Vector	AAV-hSyn-Ywhag-3xFLAG-P2A-EGFP	AAV overexpression		500nl	OBiO	/
Vector	AAV-hSyn-3xFLAG-P2A-EGFP	Control vector	/	500nl	OBiO	/
siRNA	si14-3-3γ	Gene knockdown	/	10nM	RIBOBIO	sibdm0001-5
siRNA	siNC	Control	/	10nM	RIBOBIO	sibdm0001-5
Staining reagent	DAPI	Nuclear staining	/	20uL	beyotime	C1006
Staining method	Golgi staining	Dendritic spine analysis		/	FD NeuroTechnologies	PK401
Other reagents	Transfection reagent	Cell transfection	/	2uL	ThermoFisher	11668027
Other reagents	Lysis buffer	Protein extraction		/	beyotime	P0013B

Other reagents	Protease/phosphatase inhibitors	Protein protection		50×	beyotime	P1045
Other reagents	ECL substrate	WB detection	/	8mL	vazyme	E432-02

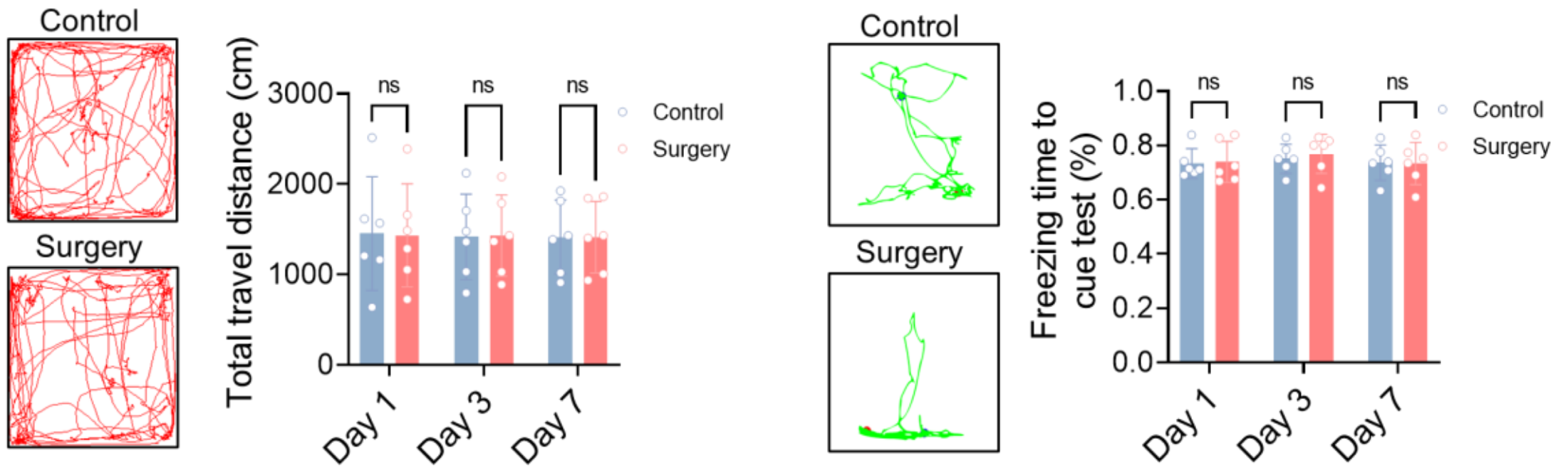

Figure S1. Surgery does not impair locomotor activity or cued fear memory in mice.

Evaluation of locomotor activity and cued associative memory at postoperative days 1, 3, and 7 (n = 6 per group). Left panels: Open Field Test (OFT) results, including representative movement trajectories and quantitative analysis of the total distance traveled (cm). No significant differences were observed between the Control and Surgery groups, indicating that tibial fracture with intramedullary nailing did not induce persistent motor deficits or exploratory behavioral changes. Right panels: Cued Fear Conditioning (FC) test results, including representative activity tracks and the percentage of freezing time during the cue test phase. There were no significant differences in freezing levels between groups across all time points, suggesting that the surgical procedure did not affect non-hippocampal-dependent associative memory or basic sensory perception. Data are presented as mean ± SD. Statistical analysis was performed using two-way ANOVA followed by Sidak's multiple comparisons test. ns, non-significant ($P > 0.05$).

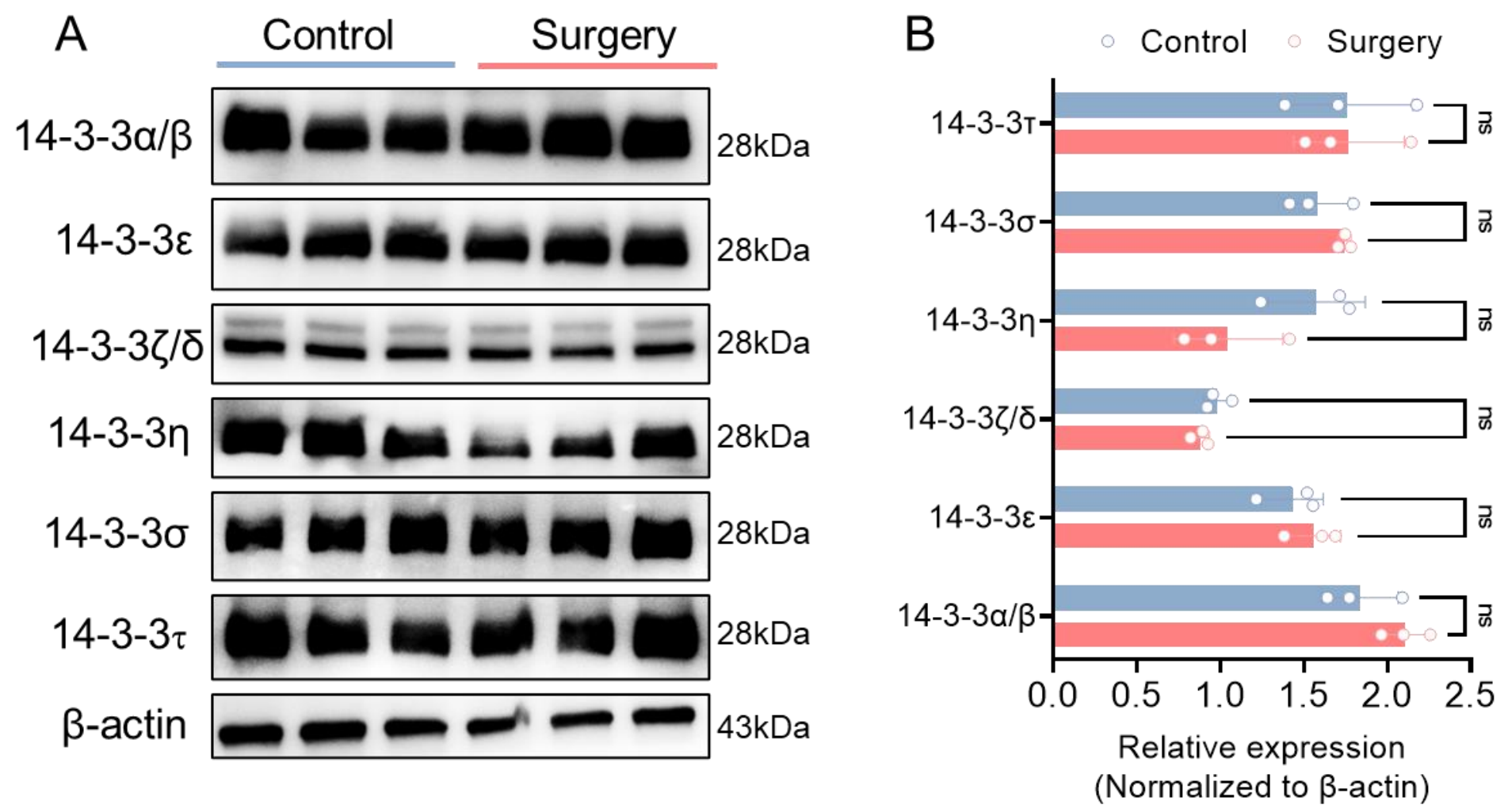

Figure S2. Differential expression of 14-3-3 isoforms in response to surgery

(A) Representative western blot images showing the expression of multiple 14-3-3 isoforms (τ, σ, η, ζ/δ, ε, and α/β) in control and surgery groups. β-actin was used as a loading control. (B) Quantification of protein levels normalized to β-actin. Among the 14-3-3 family members examined, selective alterations were observed following surgery, with 14-3-3γ showing a distinct expression pattern compared to other isoforms. Data are presented as mean ± SEM (n = 3 per group). Statistical significance was determined using two-sided unpaired *t*-tests; ns, not significant.

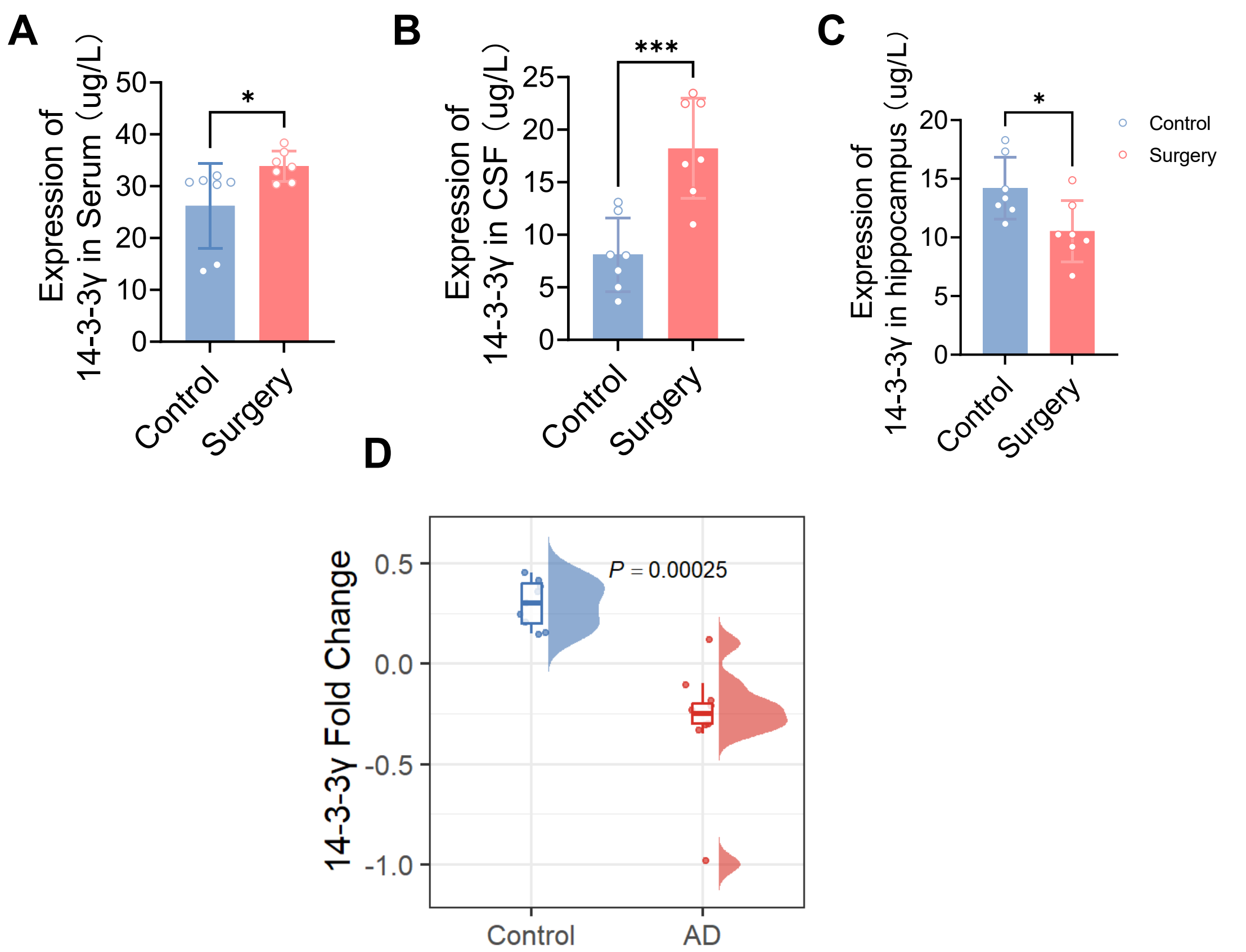

Figure S3. Divergent expression patterns of 14-3-3γ in biofluids and brain tissues across murine and human cognitive impairment models.

(A–C) 14-3-3γ levels in a three-month-old C57BL/6 mouse model of postoperative cognitive dysfunction (POCD) induced by tibial fracture with intramedullary nailing under general anesthesia (n = 7 per group). Protein expression was quantified by ELISA, showing a significant postoperative increase in both (A) serum and (B) cerebrospinal fluid (CSF), contrasted by a significant downregulation in (C) hippocampal lysates. (D–F) Validation of 14-3-3γ expression patterns in clinical cohorts and proteomic databases. (D) Spatial proteomics of human post-mortem brain tissue (adapted from Xu et al., Commun Biol, 2019) showing a significant reduction (negative fold change) in 14-3-3γ expression in Alzheimer's Disease (AD) brains compared to healthy controls (P = 0.00025). Data in (A–C) are expressed as mean ± SD. Data in D are presented as box-and-whisker plots superimposed with violin plots to illustrate data distribution. Statistical significance was determined using a two-tailed Student's t-test. *$P < 0.05$, ***$P < 0.001$.
